# Structural Interconversions and Guest Binding Properties of Tetrakis(formylpyridine)-Based *Pseudo*-Cubic and Trigonal-Prismatic Metal–Organic Capsules

**DOI:** 10.1021/jacs.5c16191

**Published:** 2025-11-17

**Authors:** Yuyin Du, Tanya K. Ronson, Yuchong Yang, Jonathan R. Nitschke

**Affiliations:** Yusuf Hamied Department of Chemistry, 2152 University of Cambridge, Cambridge CB2 1EW, U.K.

## Abstract

Here, we report a new tetrakis­(formylpyridine) subcomponent that was designed to assemble with anilines and Zn^II^ to afford a set of structurally distinct metal–organic cage structure types. By modulating the metal-to-ligand stoichiometry, we obtained a *pseudo*-cubic Zn_8_L_6_ cage and an open Zn_6_L_3_ trigonal prism, the former featuring a diastereomeric configuration of faces and vertices that had not been previously observed. Addition of a tritopic subcomponent yielded a Zn_6_L_3_L′_2_ heteroleptic capped trigonal prism, which could also be prepared via a combination of the homoleptic cages formed by the two individual ligands. The capped trigonal prism encapsulated the pollutant perfluorobutanesulfonate and the oxidant tetracyanoquinodimethane, both technologically relevant guests.

## Graphical abstract

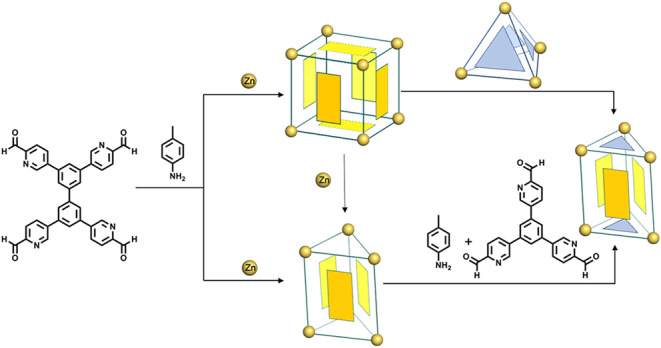

## Introduction

Metal–organic cages (MOCs) are a versatile class of discrete supramolecular architectures, prepared via the self-assembly of metal ions and organic ligands.^1^ By careful design of their building blocks, MOCs can be prepared with different polyhedral geometries, which encapsulate guest species and exhibit diverse physicochemical properties. The directional and predictable nature of metal–ligand coordination bonds enables control over the shape, size, and functionality of these assemblies. Due to their tunable cavities and host–guest chemistry, MOCs have found uses in fields that include catalysis,^2^ drug delivery,^3^ chemical separations,^4^ and the stabilization of sensitive guests through protective encapsulation.^5^

Beyond the static structures of MOCs, their ability to undergo dynamic structural transformations can enable the design of responsive and adaptive molecular systems.^6^ These transformations can be triggered by various external stimuli, including addition of metal ions^7^ or ligands,^8^ guest binding,^9^ solvent conditions,^10^ or light irradiation.^11^ Such adaptive behaviors pave the way for developing novel stimuli-responsive materials. Of particular interest for this study, stoichiometric control in self-assembly processes can provide a rational approach to access different cage structures from the same ligand,^12^ and post-assembly modifications^13^ can allow conversion between structures through the incorporation of additional components.

Among the various geometries that MOCs can adopt, M^II^_8_L_6_ *pseudo*-cube cages formed from octahedral metal ions and rectangular ligands are of particular interest due to their stereochemical diversity. Such M_8_^II^L_6_ architectures can in principle adopt any of 2048 isomeric conformations.^14^ This complexity arises from combinations of the relative orientations of the rectangular faces, together with the Δ or Λ stereochemistries of the metal centers. Despite this rich configurational space, few of these discrete diastereomers have been observed experimentally.^14a,15^ Different diastereomeric configurations possess different arrangements of panels around the central cavity, with correspondingly different guest binding abilities.^16^ The *pseudo*-cubes prepared so far incorporate tetramine subcomponents as the cage faces,^14a,15^ whereas the introduction of tetrakis­(formylpyridine)­s may favor new diastereomeric conformations owing to their distinct coordination vectors and conformational preferences, as compared with tetramine subcomponents.

Here we report the synthesis of tetrakis­(formylpyridine) subcomponent **A**, and its self-assembly with anilines and zinc­(II). By controlling the metal:ligand stoichiometry, a Zn_8_ **L** ^**A**^_6_ *pseudo*-cube cage and a Zn_6_ **L** ^**A**^_3_ open trigonal prism were formed. *Pseudo*-cube **1** adopted novel *D*_2_-symmetric diasteromeric configurations. The addition of tris­(formylpyridine) subcomponent **C** resulted in the formation of heteroleptic capped trigonal prism **3**. Capped trigonal prism **3** was also formed through reaction of the homoleptic Zn_8_ **L** ^**A**^_6_ cage **1** and Zn_4_ **L** ^**C**^_4_ tetrahedral cage **4**.

## Results and Discussion

Subcomponent **A** was synthesized as shown in Scheme S2 (Figures S2–S6). As illustrated in Figure 1a, subcomponent **A** (3 equiv), *p*-toluidine (Subcomponent **B**, 12 equiv), and zinc­(II) trifluoromethanesulfonimide (triflimide or ^–^NTf_2_, 4 equiv) reacted under microwave (MW) conditions (120 °C) in acetonitrile to form Zn_8_ **L** ^**A**^_6_ *pseudo*-cube **1**. Electrospray ionization-mass spectrometry (ESI-MS) confirmed its Zn_8_L^**A**^_6_ composition (Figures S15 and S16). The ^1^H NMR spectrum of **1** (Figure S7) was complex with many signals, which we ascribe to the presence of multiple diastereoisomers. ^1^H NMR diffusion-ordered spectrum (DOSY) measurements (Figure S13) showed that all peaks attributed to **1** have the same diffusion coefficient, consistent with the formation of diastereomeric species with similar hydrodynamic radii rather than a mixture of assemblies with fundamentally different frameworks. The ^1^H–^13^C heteronuclear single-quantum correlation (HSQC) NMR spectrum revealed 12 major imine peaks, consistent with the presence of 12 distinct proton environments in the solution state (Figure 1b). The complex ^1^H NMR spectrum was assigned by using different two-dimensional NMR techniques (Figures S8–S12). Zn_8_ **L** ^**A**^_6_ *pseudo*-cube **1′** was also obtained when *p*-anisidine was used in place of *p*-toluidine. The ^1^H NMR spectrum of **1′** is similar to that of **1** (Figures S18–S23), suggesting that **1** and **1′** may adopt the same diastereomeric conformation in the solution state.

**Figure 1:**
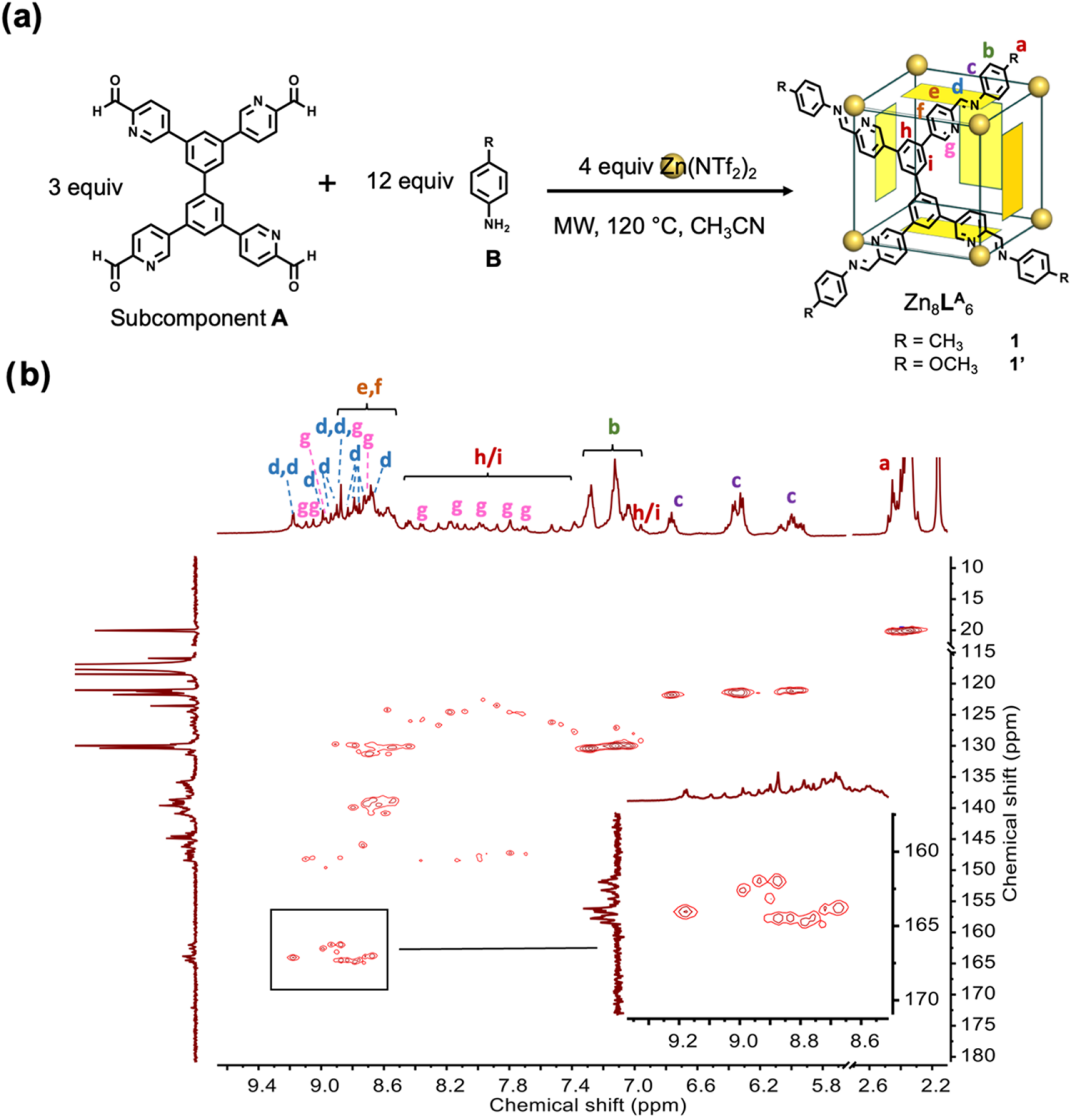
(a) Subcomponent self-assembly of Zn^II^_8_ **L** ^**A**^_6_ *pseudo*-cubes **1** and **1′**. (b) ^1^H–^13^C HSQC spectrum (500 MHz, 298 K, CD_3_CN) of cage **1**, with a blow-up of the imine region.

X-ray quality crystals for **1** and **1′** were obtained as detailed in Supporting Information Section 5. Single-crystal X-ray diffraction using synchrotron radiation at the Diamond Light Source revealed the solid-state structures of **1** and **1′**. Both consist of a Zn_8_ **L** ^**A**^_6_ assembly with a *pseudo*-cubic geometry. Eight Zn^II^ centers describe the vertices of a *pseudo*-cube, adopting a homochiral configuration within each cage. Six ligands define the *pseudo*-cube faces. However, distinct diastereomeric configurations were observed in these two structures, differing in the relative orientations of the central ligand panels. As illustrated in Figure 2a, the crystal structure of **1** showed a *pseudo*-cube where the long axis of each **A** residue is oriented parallel to the long axis of the moiety paneling the opposite face, parallel to the long axes of the residues on two of the adjacent faces, and perpendicular to the long axes of residues paneling the remaining two adjacent faces. This arrangement gives rise to *D*_2_ point symmetry, with three orthogonal *C*_2_ axes. This conformation gives six magnetically distinct proton environments (Figure S24). The internal cavity volume for the crystal structure was calculated to be 748 Å^3^.^17^ The distance between the farthest atoms is 33.5 Å, consistent with DOSY NMR measurements (Figure S13), which provided a hydrodynamic radius of 17.1 Å. The average Zn^II^···Zn^II^ distance is 11.5 ± 0.3 Å. The mean difference in *N*_imine_···*N*_imine_ separations between the two **A** residues meeting at a given edge (Δ­(*N*_imine_···*N*_imine_)) is 0.13 ± 0.07 Å (Figure S58). This distance is consistent with our previous finding that when Δ­(*N*_imine_···*N*_imine_) is less than 2 Å, a *pseudo*-cube edge forms from a pair of Zn^II^ centers with the same handedness.^14a^

**Figure 2:**
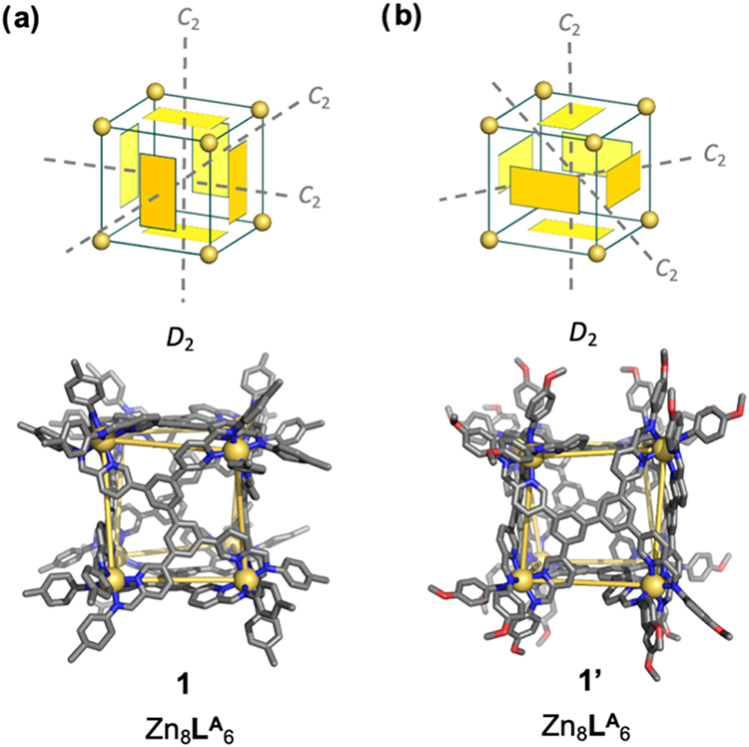
Crystal structures and cartoon illustrations of the face configurations of (a) **1** and (b) **1′**. Hydrogen atoms, solvents, anions, and disorder are omitted for clarity. CCDC number 2488117 was assigned to **1**, and 2488118 to **1′**.

The crystal structure of **1′** revealed a *pseudo*-cubic conformation where the orientations of the rectangular ligands are different from those in **1** (Figure 2b). In four of the rectangular faces of **1′**, the long axis of each **A** residue is oriented parallel to the long axes of the residues on three of the adjacent faces and an opposite face, and perpendicular to the long axes of the **A** residue paneling the remaining adjacent face. For the other two rectangular faces, the long axis of each **A** residue is oriented parallel to the long axes of the **A** residues defining two of the adjacent faces and perpendicular to those of the remaining two adjacent faces and the opposite face. Although this configuration is a diastereomer of the configuration adopted by **1**, this structure also gives overall *D*_2_ symmetry, again with three perpendicular *C*_2_ axes, resulting in six magnetically distinct proton environments (Figure S24). The diastereomeric configuration and face orientation adopted by **1′** has not previously been observed experimentally (Figure S25). Its internal cavity volume was calculated to be 781 Å^3^.^17^ The average Zn^II^···Zn^II^ distance is 11.4 ± 0.3 Å. The mean difference in imine nitrogen separations (Δ­(*N*_imine_···*N*_imine_)) is 0.1 ± 0.02 Å, again predicted to favor Zn^II^ centers with the same handedness.^14a^

Although crystallography revealed two different diastereomeric configurations for **1** and **1′**, solution-state ^1^H NMR showed 12 imine peaks in both cases, consistent with the simultaneous formation of these two diastereomers during self-assembly. Variable-temperature (VT) NMR was carried out to probe the interconversion of the two diastereomers. As shown in Figure S17, only changes attributable to the decoalescence of peaks corresponding to the freezing out of molecular motions were observed, as opposed to the appearance of new sets of product peaks or an increase in the intensity of one set of peaks, indicating that no significant interconversion occurs between the two major diastereomers over the temperature range studied, consistent with comparable thermodynamic stability of the two species. The formation of these two diastereomers as majority products was also supported by the ^19^F NMR spectrum of **1** (Figure S14), which shows two major resonances, assigned to ^–^NTf_2_ encapsulated within the two major diastereomers, along with several minor peaks, attributable to encapsulated ^–^NTf_2_ in less abundant species. We infer that the two diastereomers coexist in comparable amounts in solution, but that one configuration was selectively obtained in each crystal structure due to favorable crystal packing effects under the different crystallization conditions employed.

Subcomponent **A** (3 equiv) also reacted with *p*-toluidine **B** (12 equiv) and Zn­(NTf_2_)_2_ (6 equiv) in acetonitrile under MW conditions (120 °C) to produce Zn_6_ **L** ^**A**^_3_ open-ended trigonal prism **2** (Figure 3a). ESI–MS revealed a sharp set of signals, corresponding to charge states from +7 to +3, all of which corresponded to a Zn_6_ **L** ^**A**^_3_ composition (Figures S33 and S34). The ^1^H NMR and ^1^H–^13^C HSQC spectra of **2** showed two sets of peaks in a 1:1 ratio, suggesting two magnetically distinct chemical environments for the ligand protons of **2** (Figures S26 and S30). The DOSY spectrum of **2** showed that all of its signals had the same diffusion coefficient (Figure S31). All of the protons of **2** were assigned using two-dimensional NMR techniques (Figures S26 and S28–S30). Despite more than 100 crystallization attempts using a variety of solvents and counteranions, single crystals of **2** suitable for X-ray diffraction were not obtained. We attribute this to the structural flexibility and open-ended nature of **2**, which may prevent the formation of a well-ordered crystal lattice. An MM3 ^18^ model of **2** was thus constructed based on these solution experiments (Figure 3b). Three tetratopic ligands span the rectangular faces of the trigonal prism, while the top and bottom faces are empty. The structure has *D*_3_ symmetry, with two distinct ligand proton environments. The Zn^II^ centers are bound by only two pyridyl-imine arms. We infer each Zn^II^ center to be coordinated by two additional solvent molecules to saturate its preferred octahedral coordination geometry, as observed in previous reports of structures featuring Zn^II^ coordinated by two pyridyl-imine arms.^8a,19^ These labile ligands are readily lost during ESI–MS ionization, which detects only the Zn_6_L_3_ core.

**Figure 3:**
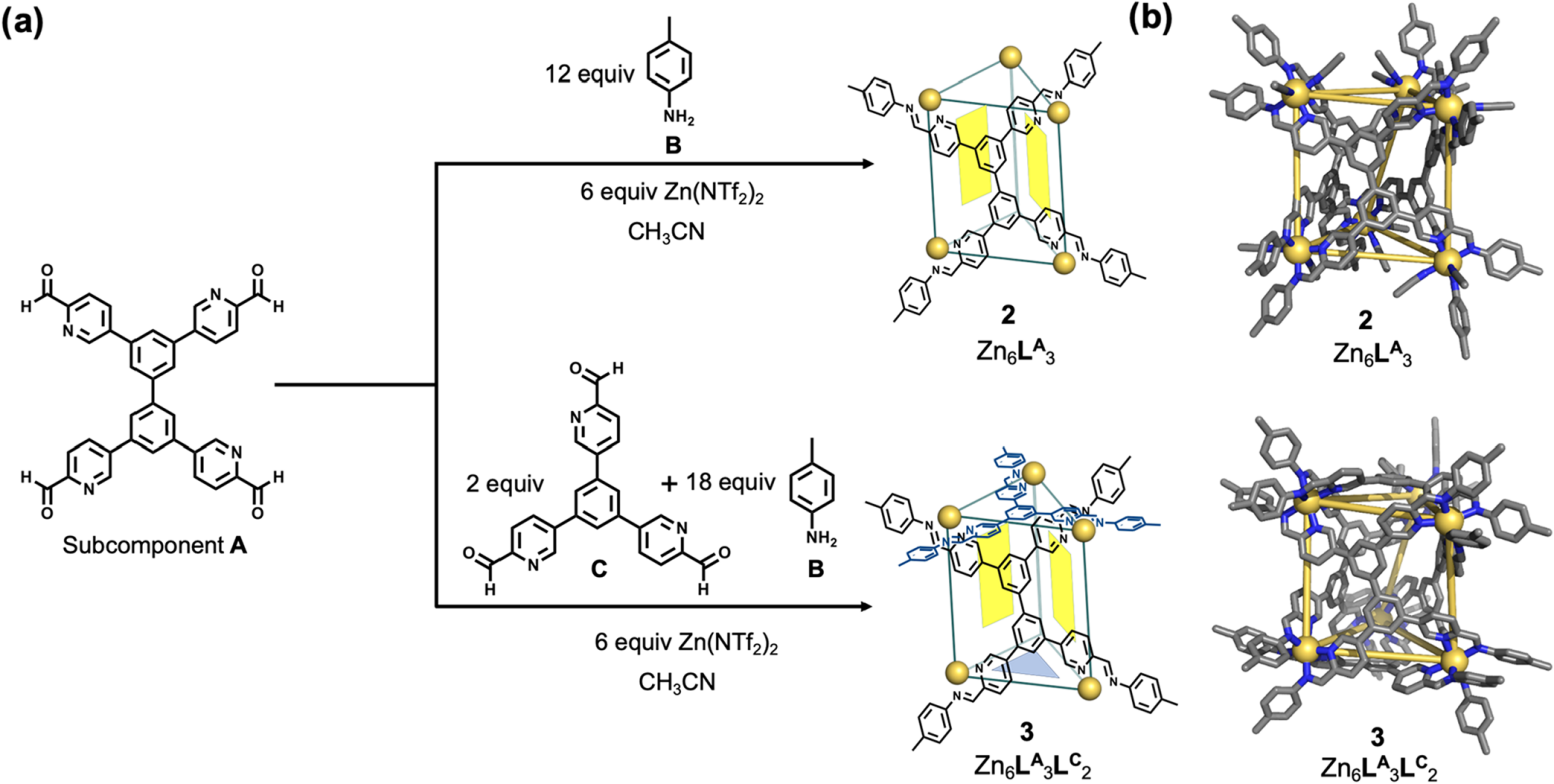
(a) Assembly of subcomponent **A** into (top) open-faced trigonal prism **2** from **B** and Zn^II^ and (bottom) closed trigonal prism **3** from **C**, **B**, and Zn^II^. (b) MM3 ^17^ model of cage **2** and the X-ray crystal structure of cage **3**. Hydrogen atoms, solvents, anions, and disorder are omitted for clarity. CCDC 2488119 was assigned to cage **3**.

We then investigated whether an additional tritopic ligand of the right size could cap the empty faces of **2**. Heteroleptic Zn_6_ **L** ^**A**^_3_ **L** ^**C**^_2_ capped trigonal prism **3** was thus prepared via self-assembly of subcomponents **A** (3 equiv), **B** (18 equiv), and **C** (2 equiv) with Zn­(NTf_2_)_2_ (6 equiv) under MW conditions at 120 °C, as shown in Figure 3a. ESI-MS confirmed the Zn_6_ **L** ^**A**^_3_ **L** ^**C**^_2_ composition of **3** (Figures S42 and S43). All of the ^1^H NMR signals of **3** were assigned using different two-dimensional NMR techniques (Figures S35–S39). Single-crystal X-ray diffraction analysis revealed the solid-state structure of **3**, as shown in Figure 3b. Six Zn^II^ centers, all with the same stereochemical configuration, form the vertices of the capped trigonal prism. Three tetratopic ligands cover the quadrilateral faces of the prism, while two tritopic ligands cover the top and bottom triangular faces, giving rise to the *D*_3_ symmetry. The average Zn^II^···Zn^II^ distance is 11.5 ± 0.1 Å, similar to those observed in homoleptic **1** and **1′**, and previously reported Zn_4_ **L** ^**C**^_4_ tetrahedral cage **4** (Figure 4), which has an average Zn^II^···Zn^II^ distance of 11.6 ± 0.1 Å.^20^ The internal cavity volume of **3** was calculated to be 392 Å^3^.^17^

**Figure 4:**
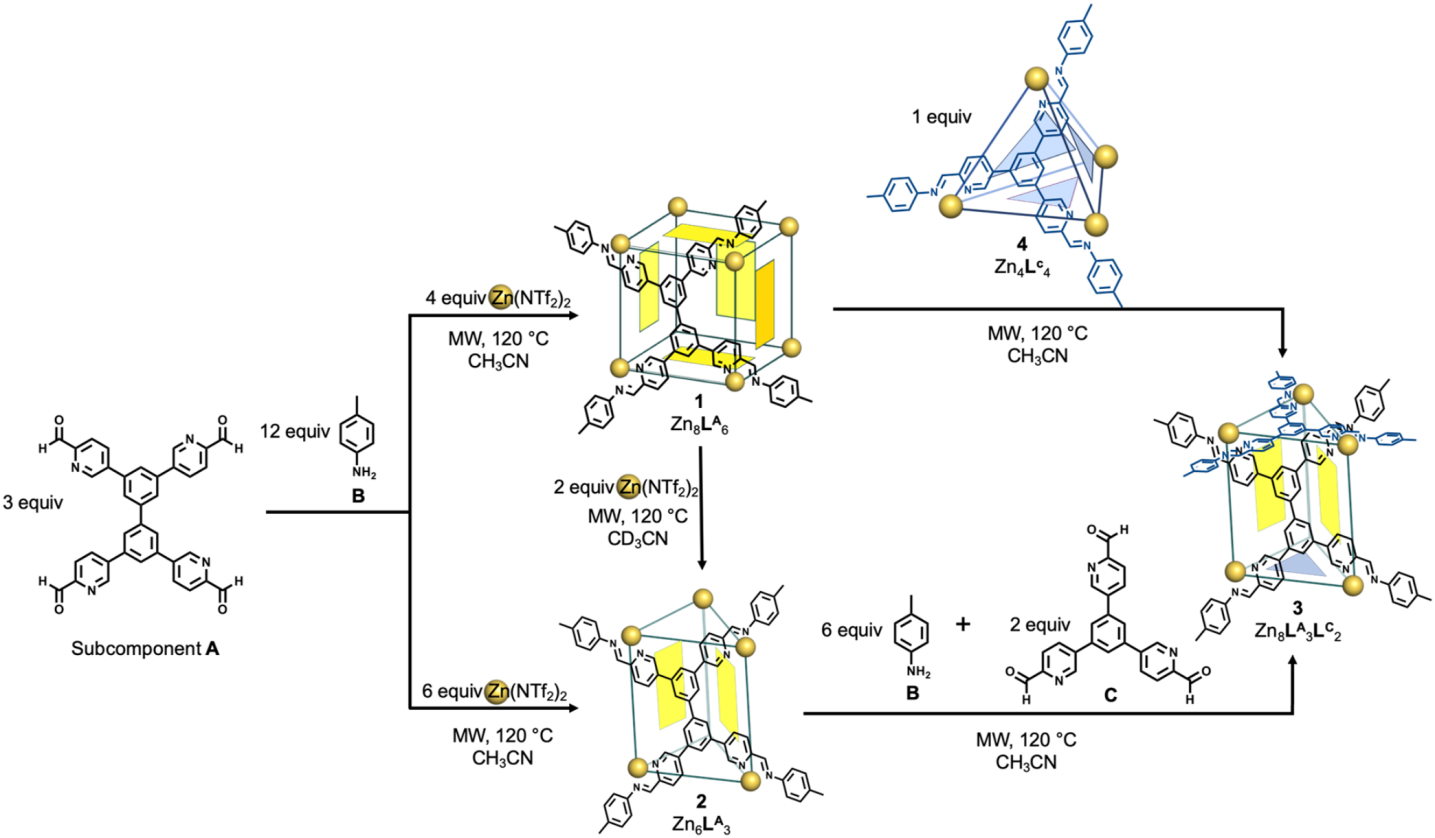
Schematic illustration of the transformation network between structures containing tetrakis­(formylpyridine) subcomponent **A**.

As shown in Figure 4, the relative stabilities of the different assemblies formed from subcomponent **A** were probed through structural transformations, which were monitored through ^1^H NMR. The addition of Zn­(NTf_2_)_2_ (4 equiv) stimulated the transformation of *pseudo*-cube **1** into open-ended trigonal prism cage **2** (Scheme S6 and Figure S44). Open-ended prism **2** transformed into capped **3** following the addition of subcomponents **B** and **C** (Scheme S7 and Figure S45). Cage **3** was also formed by mixing homoleptic cages **1** and **4** (Scheme S8 and Figure S46). The conversion of homoleptic **1** and **4** into heteroleptic **3** may be driven by entropy, as reported previously.^21^ The transformation of **1** into **3** may also relieve strain engendered by the mismatched edge lengths of the **A** residues within **1**.^22^ The relief of mismatched edge strain may also stabilize coordinatively unsaturated **2** relative to **1** in the presence of excess Zn^II^. A detailed mechanistic study was not performed, as the conversion proceeds only under MW heating at 120 °C, precluding equilibrium studies such as van ’t Hoff analysis.

The guest encapsulation properties of cages **1**–**3** were then investigated. Cage **1**, **2**, or **3** was mixed with a prospective guest in an NMR tube, and complexation was probed using ^1^H and ^19^F NMR spectroscopy. *Pseudo*-cube **1** did not show evidence for binding the neutral guests shown in Figure S55. We infer the lack of binding to be due to competitive binding of counteranions by the more highly charged cage **1**, as compared to trigonal prism **3**, as suggested by ^19^F NMR (Figure S14) and geometric mismatches between the cage and prospective guests. Although cage **3** was observed to bind ^–^NTf_2_ inside its cavity (Figure S41) in solution, **3** also bound anionic perfluorobutanesulfonate (**G1**) and neutral tetracyanoquinodimethane (TCNQ or **G2**). As shown in Figures S47–S49, the ^1^H NMR signals of **3** and the ^19^F NMR signals of **G1** shifted after mixing **3** and **G1**, indicating a fast-exchange host–guest interaction. The inward-pointing proton H_endo‑i_ shifted significantly, whereas the outward-pointing proton H_exo‑i_ showed little chemical shift change, consistent with binding of guest **G1** inside the cavity of cage **3** (Figure S53). The counteranion-competitive binding constant of **G1** was measured by ^1^H NMR titration, as shown in Figures S51 and 52, giving a *K*_a_ = 289 ± 6 M^–1^ for **G1⊂3**. As shown in Figure S50, the intensity of the ^19^F NMR signal for encapsulated ^–^NTf_2_ decreased significantly after the addition of **G1**, which also confirms its displacement by guest **G1**. The addition of neutral guest **G2** led to a new set of ^1^H NMR peaks for cage **3**, consistent with slow-exchange host–guest interaction (Figure S54). The competitive binding constant of **G2** was obtained by performing ^1^H NMR titrations, indicating *K*_a_ = 1117 ± 124 M^–1^. Single-crystal X-ray diffraction analysis revealed the solid-state structure of the **G2**⊂**3** complex. As shown in Figure 5b, **G2** was bound in a vertical orientation inside the cage cavity. Open trigonal prism **2** did not show binding of **G2**, which we infer to be due to the lack of an enclosed cavity.

**Figure 5:**
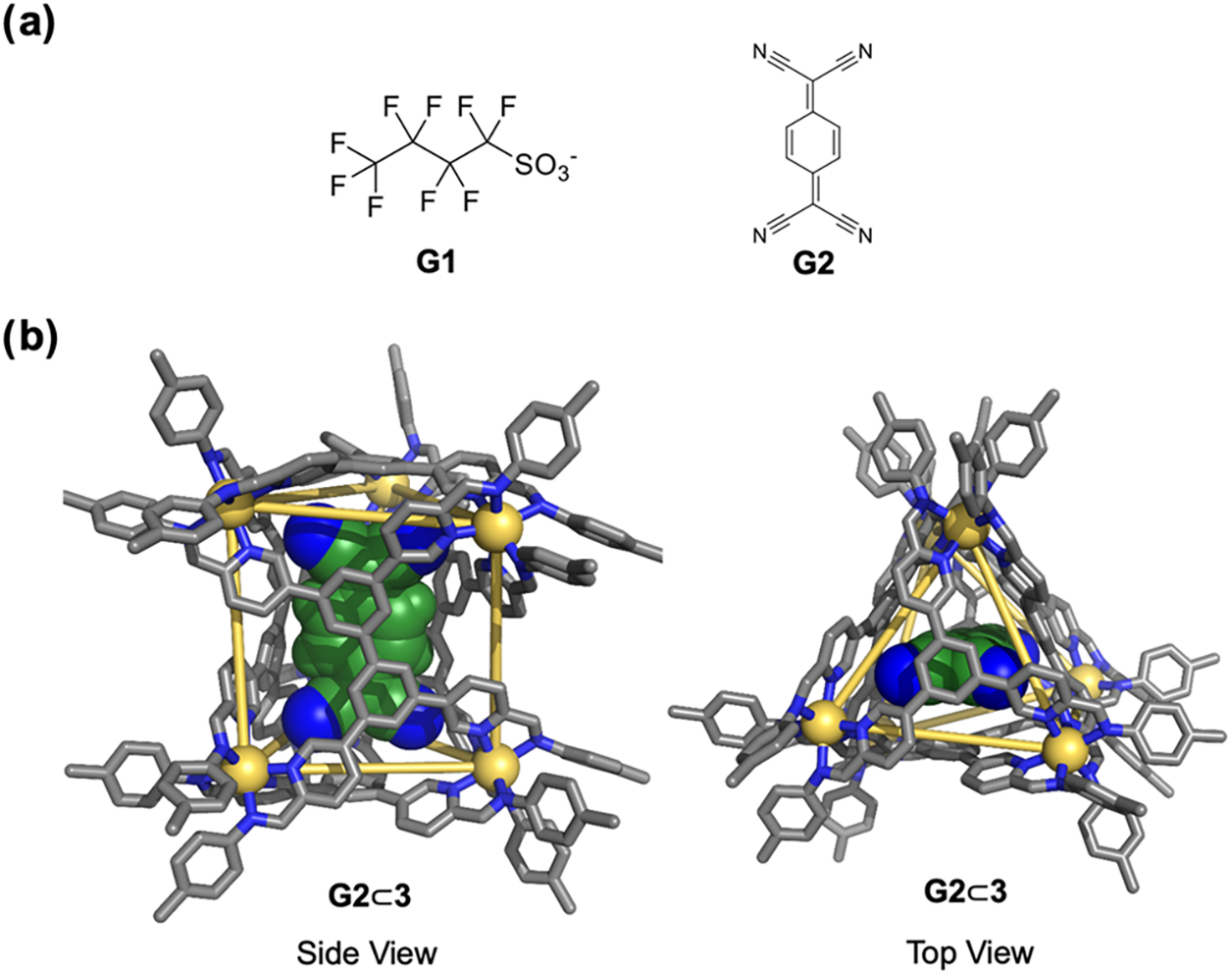
(a) Chemical structures of **G1** and **G2**, which were observed to bind in cage **3**. (b) X-ray crystal structure of **G2**⊂**3**, with **G2** shown in space-filling mode. Counterions, solvents, and disorder are omitted for clarity. CCDC 2488120 was assigned to **G2**⊂ **3**.

The electrochemical properties of **G2**, cage **3**, and **G2**⊂**3** were studied by cyclic voltammetry in acetonitrile with a tetrabutylammonium triflimide electrolyte (Figures S60 and S61). All reductions were reversible over three cycles. The first reduction wave for **G2** occurred at −0.461 V. Interestingly, the first reduction wave for **G2**⊂**3** occurred at −0.567 V. This observation indicates that reduction becomes energetically less favorable after host–guest complexation. We infer that encapsulation within cage **3** creates a protective microenvironment, stabilizing the neutral state of **G2** and increasing the energy barrier for reduction,^23^ in spite of the cationic cage framework that would be expected to stabilize the anionic form of **G2**.

## Conclusion

Tetratopic ligands that incorporate subcomponent **A** thus produced a variety of new structure types. By modulating the metal–ligand stoichiometry, either novel Zn_8_ **L** ^**A**^_6_ *pseudo*-cube **1** or Zn_6_ **L** ^**A**^_3_ open trigonal prism **2** was obtained. The new tetrakis­(formylpyrdine) ligands derived from **A** provided subtly different conformational flexibility to previously investigated tetra-anilines,^14a^ thus giving rise to a diastereomeric configuration of the Zn_8_ **L** ^**A**^_6_ *pseudo*-cube that had not previously been observed, as well as capped Zn_6_ **L** ^**A**^_3_ **L** ^**C**^_2_ trigonal prism **3** from trialdehyde **C**, through edge-length matching. Cage **3** demonstrates the ability to bind both a polyfluoroalkyl sulfonate, highlighting its potential utility in environmental pollutant capture, and TCNQ, demonstrating the ability to protect electron-deficient compounds against reduction. This approach thus expands the diversity of accessible stereochemical space for metal–organic cage design through judicious choice of subcomponent geometry, providing new means of predictive control over cage stereochemistry. Future investigations of polytopic and anisotropic formylpyridines promise to enable the formation of further new three-dimensional metal–organic capsules, with the ability to bind and sense, purify, or transform technologically relevant guests.

## Supplementary Material



## Data Availability

Any additional data related to this paper may be requested from the authors.

## Notes

Experimental procedures; NMR characterizations; and mass spectrometry data (PDF)
