# Supplementary material for: Structural Interconversions and Guest Binding Properties of Tetrakis(formylpyridine)-Based Pseudo-Cubic and Trigonal-Prismatic Metal–Organic Capsules
Source: J Am Chem Soc. 2025 Nov 17;147(47):43927–33. doi: 10.1021/jacs.5c16191 (PMC12673594; doi:10.1021/jacs.5c16191)
Supplement: Supplementary file 1 [file ja5c16191_si_001.pdf]

Supplementary Materials for

Structural Interconversions and Guest Binding Properties of Tetrakis(formylpyridine)-Based *Pseudo*-Cubic and Trigonal-Prismatic Metal-Organic Capsules

Yuyin Du, Tanya K. Ronson, Yuchong Yang, Jonathan R. Nitschke*

Yusuf Hamied Department of Chemistry, University of Cambridge, Cambridge, CB2 1EW, United Kingdom.

*Corresponding author: jrn34@cam.ac.uk (J.R.N.)

Table of Contents

1. General Information **3**

2. Synthesis and Characterisation **4**

2.1 Synthesis of compound **2** 4

2.2 Synthesis of subcomponent **A** 6

2.2 Self-assembly and characterization of cage **1** and **1’** 9

2.4 Self-assembly and characterization of cage **2** 21

2.5 Self-assembly and characterization of cage **3** 28

3. Structural transformations **35**

3.1 Structural transformation from cage **1** to cage **2** 35

3.2 Structural transformation from cage **2** to cage **3** 36

3.3 Structural transformation from cage **1** and cage **4** to cage **3** 37

4. Host-guest studies **38**

4.1 General procedures 38

4.2 **G1** binding within cage **3** 38

4.3 **G2** binding within cage **3** 44

4.4 Non-binding prospective guests 45

5. X-ray Crystallography **47**

5.1 Crystal structure of cage **1** 47

5.2 Crystal structure of cage **1’** 49

5.3 Crystal structure of cage **3** 51

5.4 Crystal structure of **G2⊂3** 53

5.5 Measurement of parameters in cage **1** and **1’** 56

6. Volume Calculations **57**

7. Electrochemistry **58**

8. References **60**

1. General Information

Materials and methods

Unless otherwise stated, all chemicals were obtained from commercial sources and used as received. Cage **4** was synthesized through previously reported procedures.[1]

Nuclear Magnetic Resonance (NMR)

NMR experiments employed Bruker AVANCE III and NEO (400 and 500 MHz) spectrometers. Chemical shifts for ^{1}H and ^{13}C NMR are reported in ppm with residual solvent as reference: trifluoroacetic acid-d, (11.5 ppm for ^{1}H, 116.5 and 164.4 for ^{13}C), chloroform (7.26 ppm for ^{1}H, 77.22 ppm for ^{13}C). Abbreviations for signal multiplicity of ^{1}H NMR spectra are shown as following: s: singlet, d: doublet, t: triplet, dd: doublet of doublets; dt: doublet of triplets; m: multiplet, br: broad.

Mass spectrometry (MS)

High-resolution electrospray ionization (HR-ESI) mass spectra were obtained with Waters Synapt G2-Si instrument (cone voltage 30 eV; desolvation temperature 353 K; ionization temperature 373 K) infused from a Harvard Syringe Pump at a rate of 10 μL per minute.

2. Synthesis and Characterisation

2.1 Synthesis of compound **2**

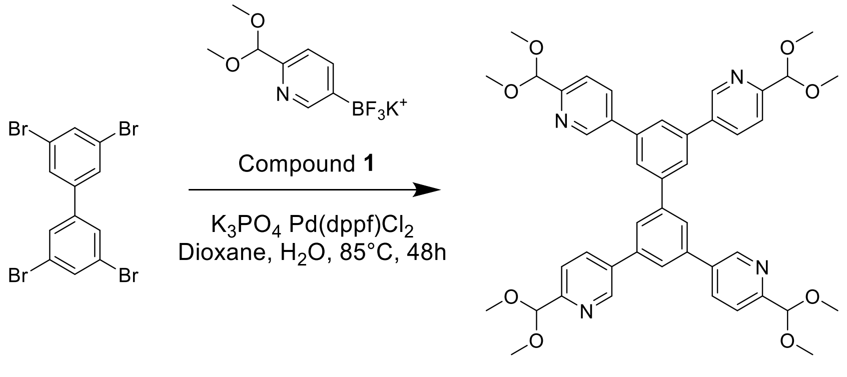

Scheme S1. Synthesis of compound **2**.

Compound **1** was synthesized according to the literature.[2]

3,3-5,5-Tetrabromo-1,1-biphenyl (500 mg, 1 equiv.), compound 1 (1.65 g, 6 equiv.), Pd(dppf)Cl_2 (172 mg, 0.2 equiv.), K_3PO_4 (2.26 g, 10 equiv.), 40 mL 1,4-dioxane and 10 mL H_2O were added to a 100 mL Schlenk flask and nitrogen was bubbled through the resulting solution for 1 hour. The reaction mixture was stirred at 85 °C for 48 hours. The solvent was removed *in vacuo* and extracted 3 times with CH_2Cl_2 (50 mL). The organic phase was collected, dried over Na_2SO_4, filtered, and concentrated *in vacuo*. The crude product was further purified by flash column chromatography on silica gel using CH_2Cl_2/MeOH = 40/1 – 20/1 as eluent affording compound **2** as a white solid (609 mg, 75%).

^{1}H NMR (500 MHz, $CDCl_3$) δ 8.95 (s, 4H), 8.04 (d, *J* = 8.0 Hz, 4H), 7.88 (s, 4H), 7.81 (s, 2H), 7.69 (d, *J* = 8.1 Hz 4H), 7.82 (m, 20H), 5.47 (s, 4H), 3.46 (s, 24H).

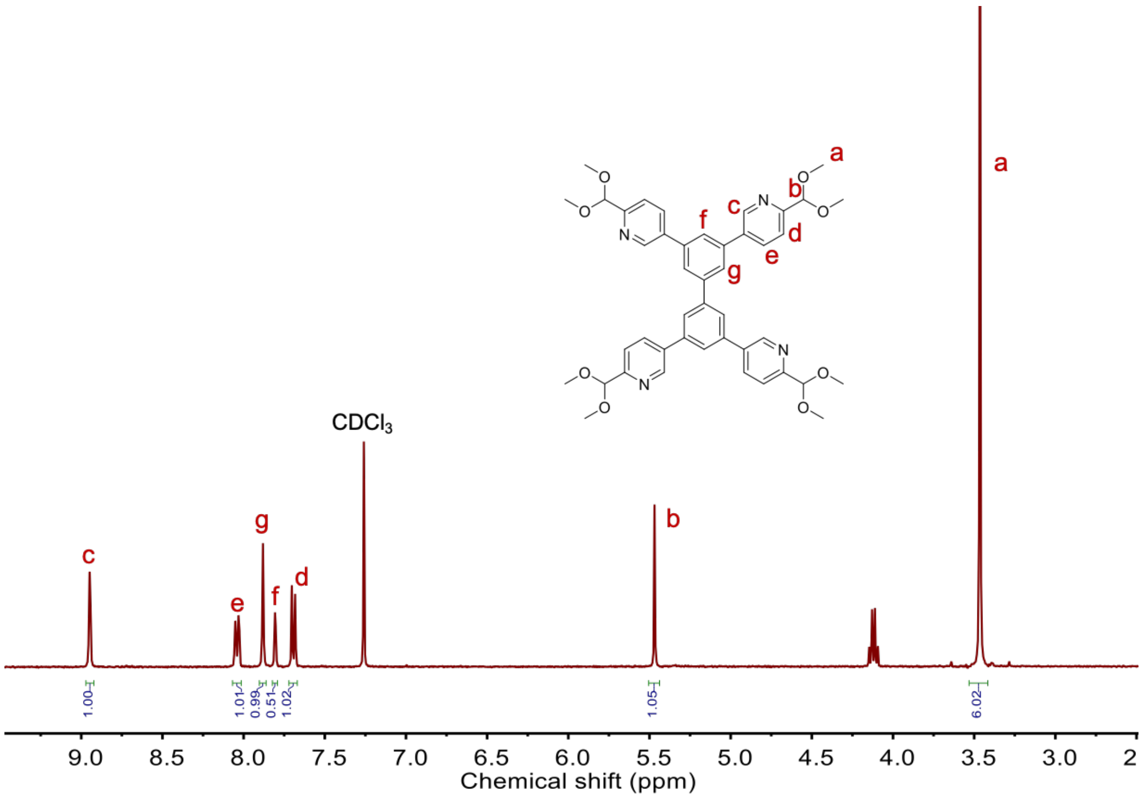

Figure S1. ^{1}H NMR spectrum (500 MHz, 298 K, $CDCl_3$) of compound **2**.

2.2 Synthesis of subcomponent **A**

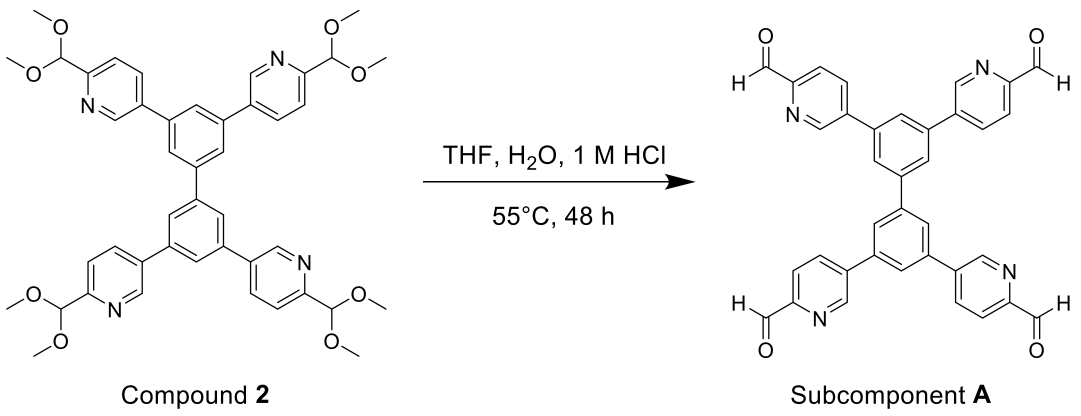

Scheme S2. Synthesis of Subcomponent **A**.

Compound **2** (300 mg, 1 equiv.), 20 mL THF, 1 M HCl and 20 mL H_2O were added to a 100 mL round bottom flask. The reaction mixture was stirred at 55 °C for 48 hours. The reaction mixture was cooled to room temperature and neutralized with saturated Na_2CO_3. The precipitate was collected and washed with 30 mL H_2O and 30 mL MeOH and then isolated by centrifugation. The precipitate was further washed twice with 30 mL CH_2Cl_2 . The precipitate was dried overnight affording subcomponent **A** as a white solid (209 mg, 92%).

^{1}H NMR (500 MHz, CF_3COOD) δ 10.07 (s, 4H), 9.41 (s, 4H), 9.21 (d, J = 8.2Hz, 4H), 8.67 (m, 6H).
^{13}C NMR (126 MHz, CF_3COOD) δ 182.6, 147.1, 143.7, 142.5, 142.0, 140.1, 135.1, 129.7, 129.4, 127.0.

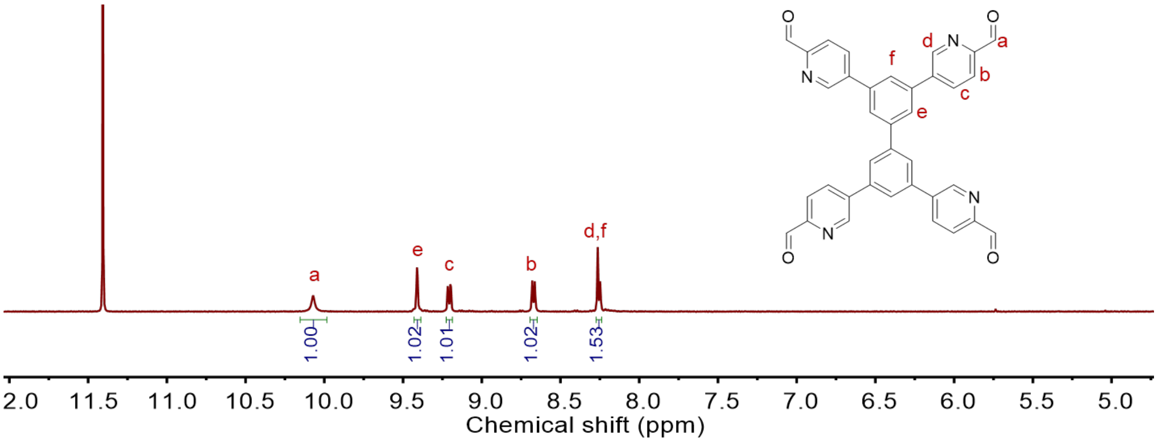

Figure S2. ^{1}H NMR spectrum (500 MHz, 298 K, CF_3COOD) of subcomponent **A**.

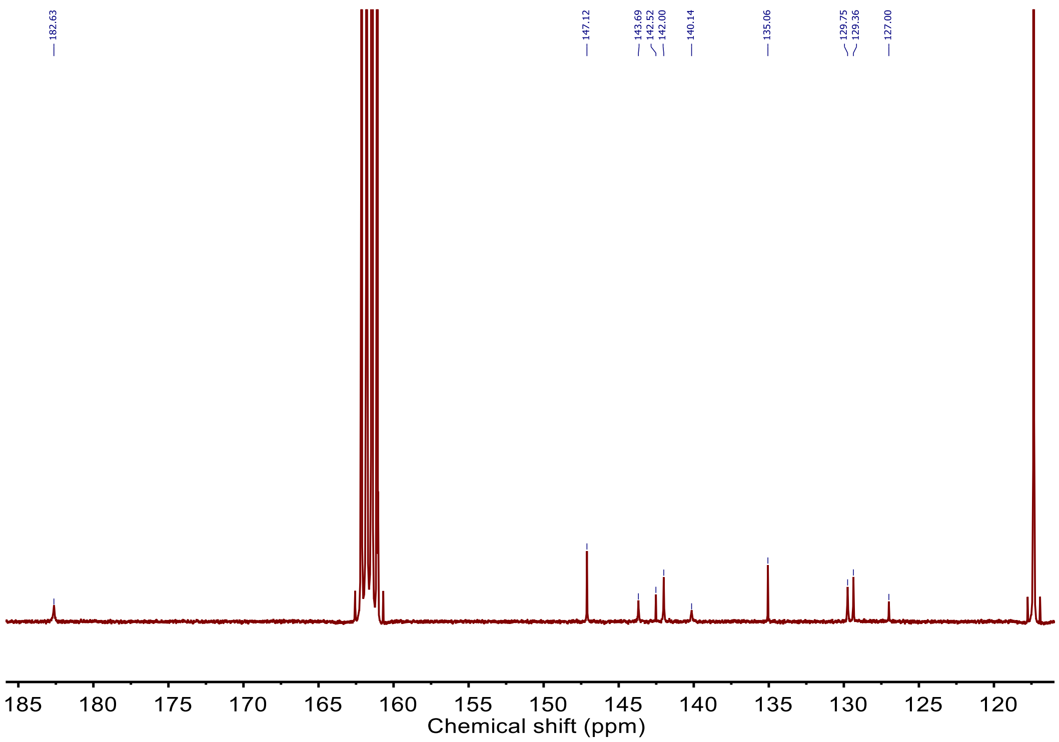

Figure S3. ^{13}C NMR spectrum (126 MHz, 298 K, CF_3COOD) of subcomponent **A**.

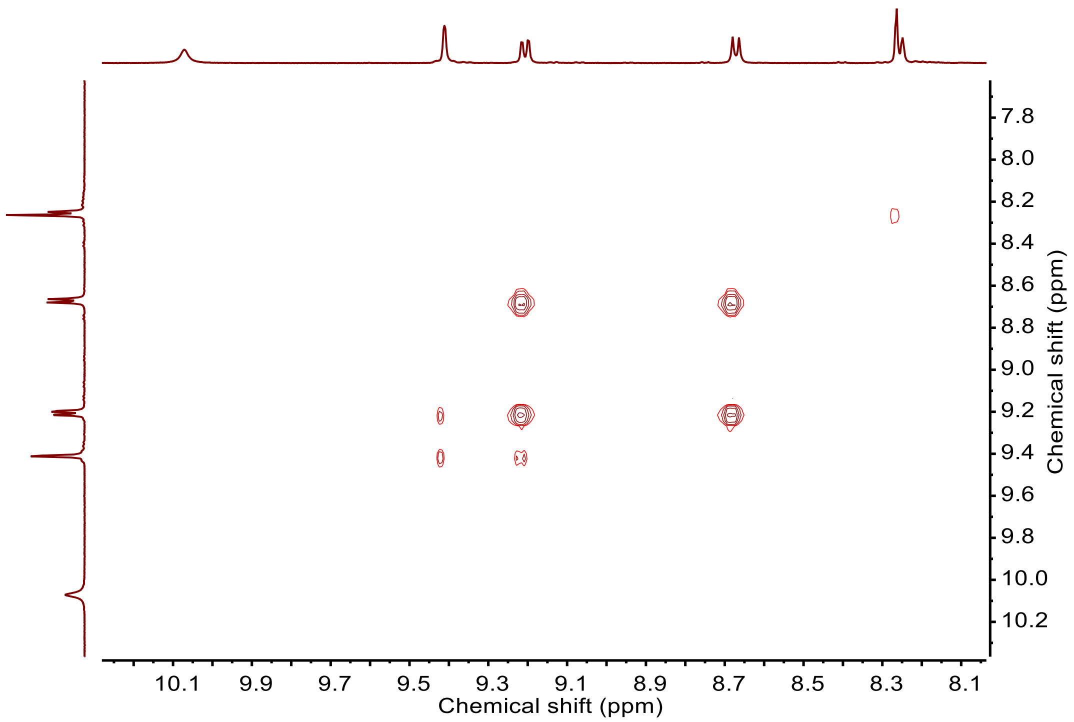

Figure S4. Partial ^{1}H-^{1}H COSY NMR spectrum (500 MHz, 298 K, CF_3COOD) of subcomponent **A**.

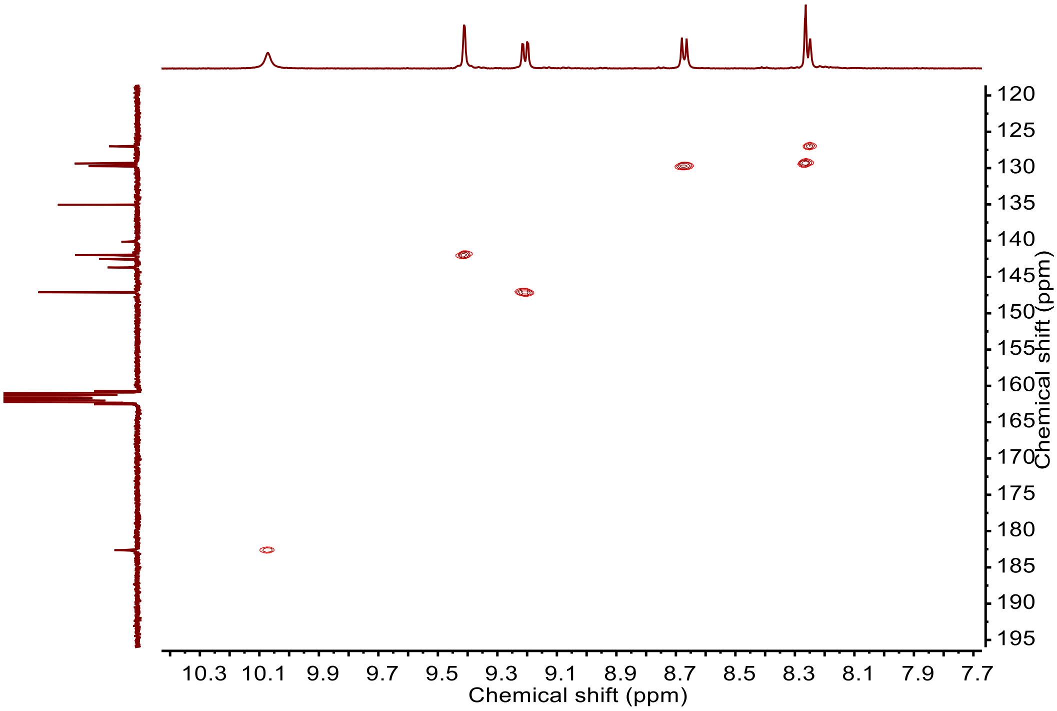

Figure S5. Partial ^{1}H-^{13}C HSQC NMR spectrum (500 MHz, 298 K, CF_3COOD) of subcomponent **A**.

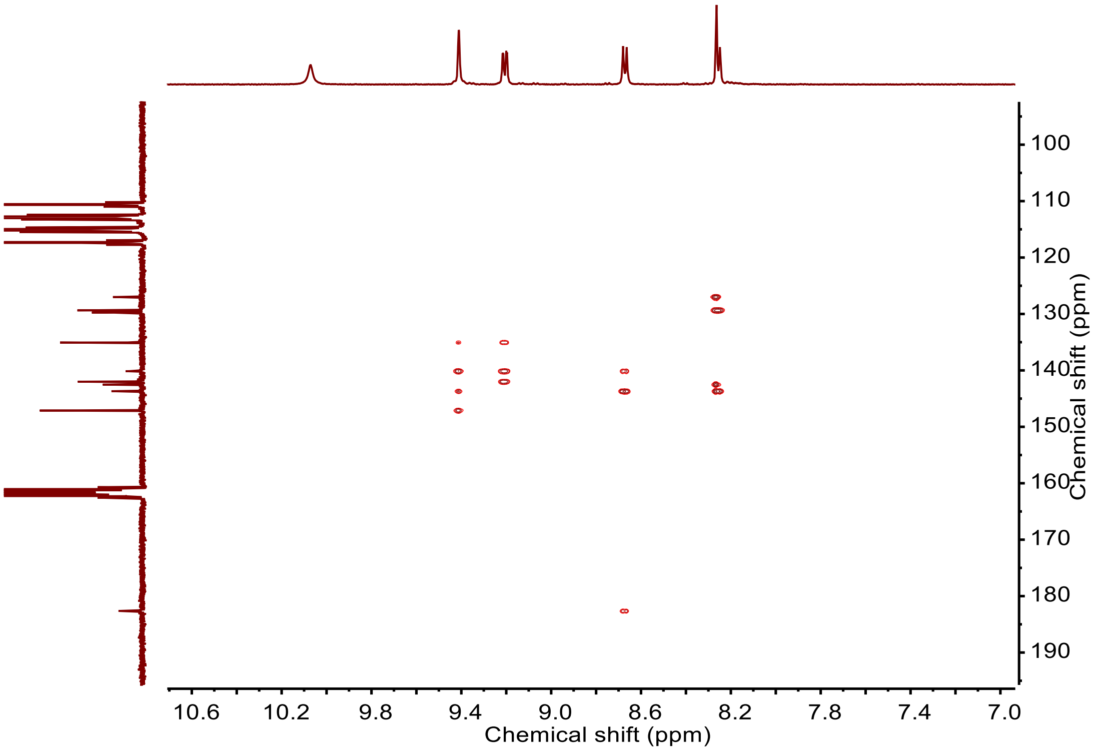

Figure S6. Partial ^{1}H-^{13}C HMBC NMR spectrum (500 MHz, 298 K, CF_3COOD) of subcomponent **A**.

2.2 Self-assembly and characterization of cage **1** and **1’**

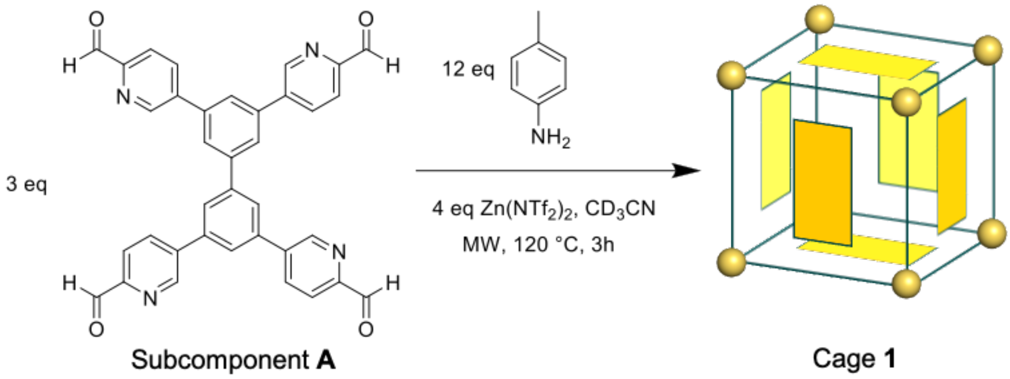

Scheme S3. Self-assembly of cage **1**.

Subcomponent **A** (10.0 mg, 17.4 µmol, 3 equiv.), *p*-toluidine (7.45 mg, 69.6 µmol, 12 equiv.), $Zn(NTf_2)_2$ (14.5 mg, 23.2 µmol, 4 equiv.) and CH_3CN (2 ml) were added to a vial, and the mixture was sonicated for 1 min. The mixture was then heated at 120 °C for 3 hours using a microwave reactor. The solvent was removed by blowing with N_2 and then Et_2O (10 ml) was added. The resulting solid was collected by centrifugation, washed three times with additional Et_2O (10 ml) and then dried under dynamic vacuum for 16 h at 298 K to give **1** as a yellow solid (22.3 mg, 2.10 µmol, yield 72.6 %).

^{1}H NMR (400 MHz, CD_3CN) δ 9.18 – 8.53 (m, 41H), 8.46 – 6.94 (m, 49H), 6.84 – 5.92 (m, 24H), 2.48 – 2.29 (m, 36H).

^{13}C NMR (126 MHz, CD_3CN) δ 164.8, 164.7, 164.5, 164.5, 164.4, 164.3, 164.1, 163.9, 163.8, 163.2, 162.7, 162.6, 162.0, 161.9, 161.7, 161.7, 161.6, 148.4, 148.4, 148.2, 148.0, 147.8, 147.6, 147.4, 147.2, 147.0, 146.9, 146.8, 146.4, 146.4, 146.3, 146.2, 145.9, 145.9, 145.6, 145.5, 144.9, 144.6, 144.5, 144.3, 144.2, 144.0, 143.8, 142.1, 141.5, 141.4, 141.2, 141.0, 140.9, 140.7, 140.5, 140.3, 140.1, 139.9, 139.7, 139.6, 139.2, 139.1, 139.0, 138.8, 138.6, 138.2, 138.1, 137.5, 137.3, 136.8, 136.5, 136.4, 136.2, 136.2, 136.1, 135.9, 135.7, 131.4, 131.3, 131.3, 131.0, 130.4, 130.1, 130.0, 130.0, 129.9, 129.8, 129.7, 128.0, 127.9, 127.8, 127.1, 126.7, 126.6, 126.2, 125.5, 124.5, 124.5, 124.2, 122.7, 122.5, 122.5, 122.2, 121.8, 121.7, 121.4, 121.3, 121.2, 119.8 (q, J = 321.3 Hz, $^{-}NTf_2$), 20.1, 20.1, 20.0.

ESI-MS (CH_3CN) m/z = 1043.65 $[\mathbf{1}(NTf_2)_8]^{8+}$, 1233.02 $[\mathbf{1}(NTf_2)_9]^{7+}$, 1485.17 $[\mathbf{1}(NTf_2)_{10}]^{6+}$, 1838.18 $[\mathbf{1}(NTf_2)_{11}]^{5}$.

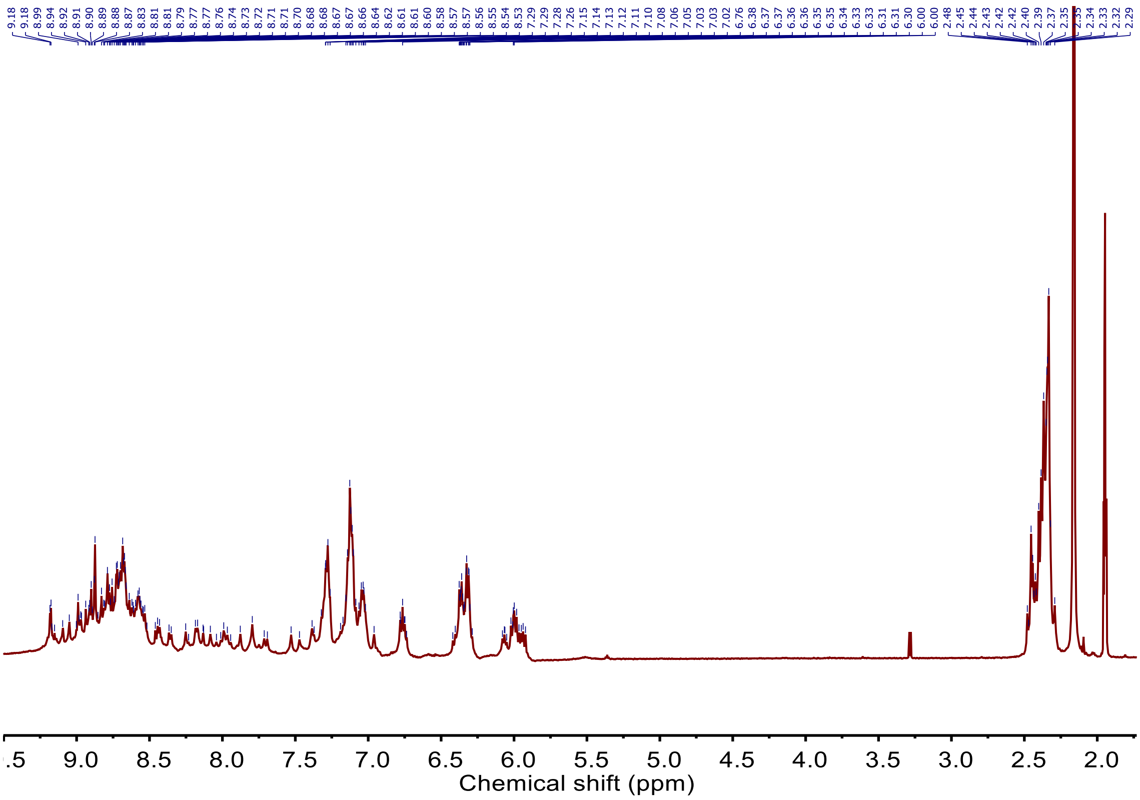

Figure S7. ^{1}H NMR spectrum (500 MHz, 298 K, CD_3CN) of cage **1**.

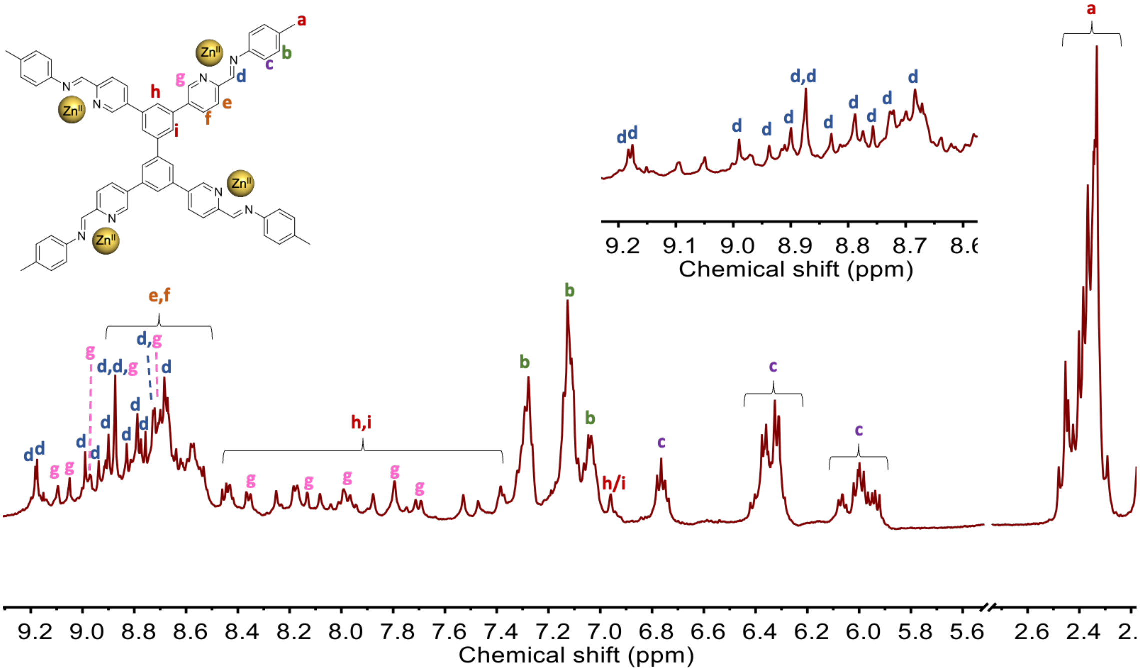

Figure S8. ^{1}H NMR spectrum (500 MHz, 298 K, CD_3CN) of cage **1** with proton assignments shown.

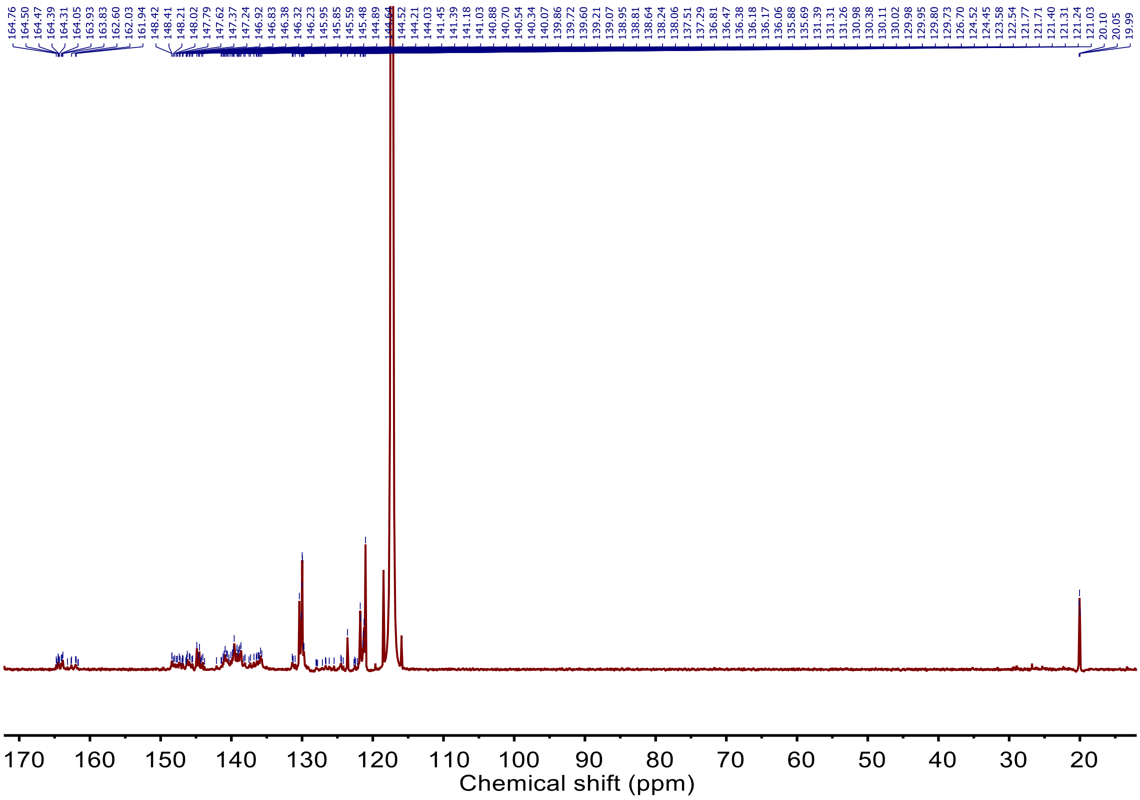

Figure S9. ^{13}C NMR spectrum (126 MHz, 298 K, CD_3CN) of cage **1**.

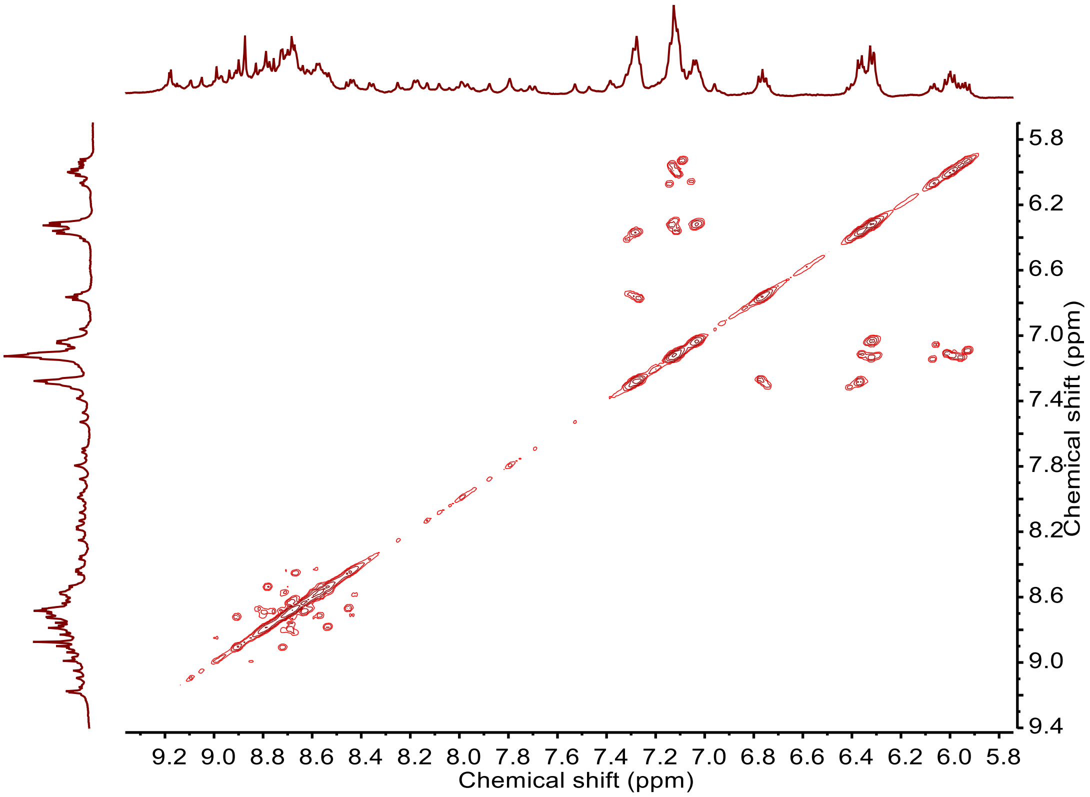

Figure S10. Partial ^1H-^1H COSY NMR spectrum (500 MHz, 298 K, CD_3CN) of cage **1**.

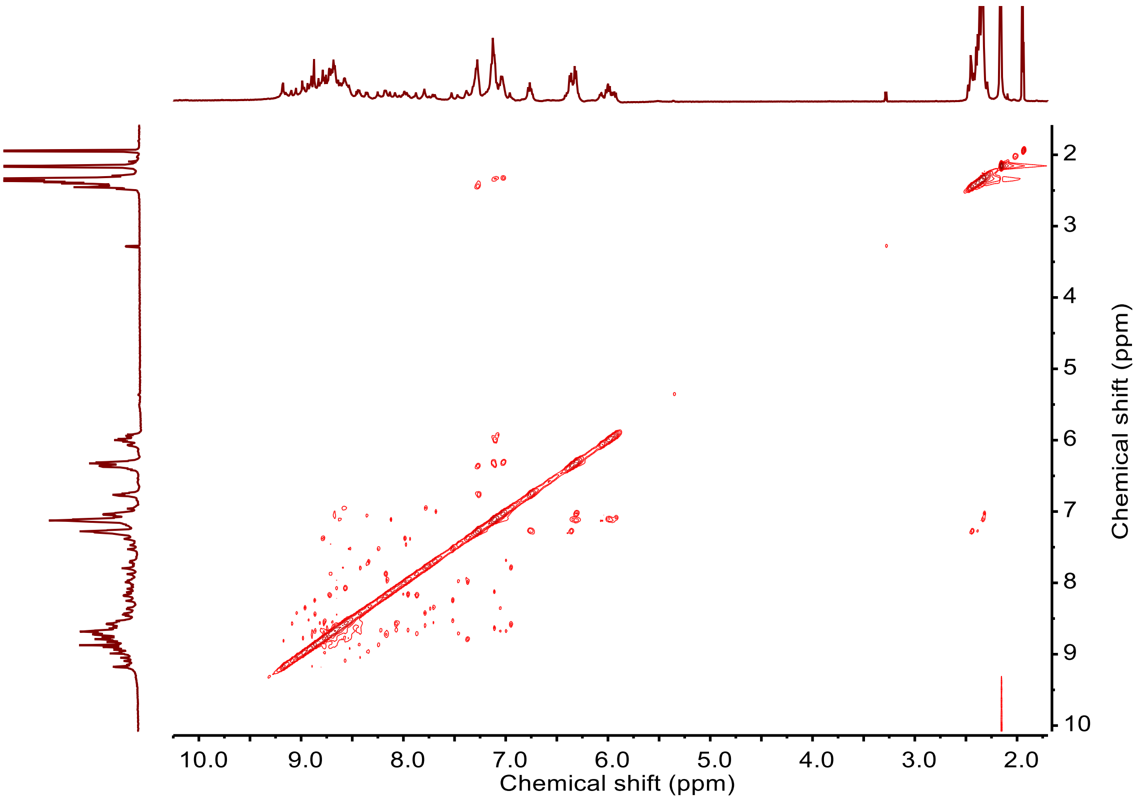

Figure S11. Partial ^{1}H-^{1}H NOESY NMR spectrum (500 MHz, 298 K, CD_3CN) of cage **1**.

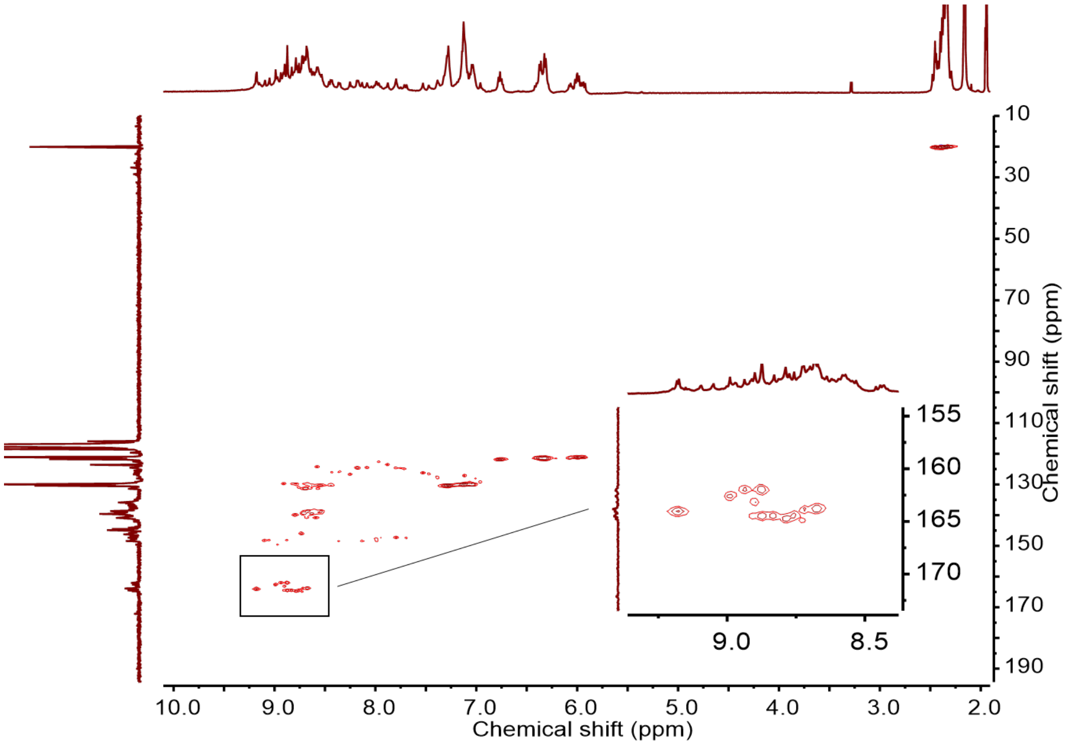

Figure S12. Partial ^{1}H-^{13}C HSQC NMR spectrum (500 MHz, 298 K, CD_3CN) of cage **1**.

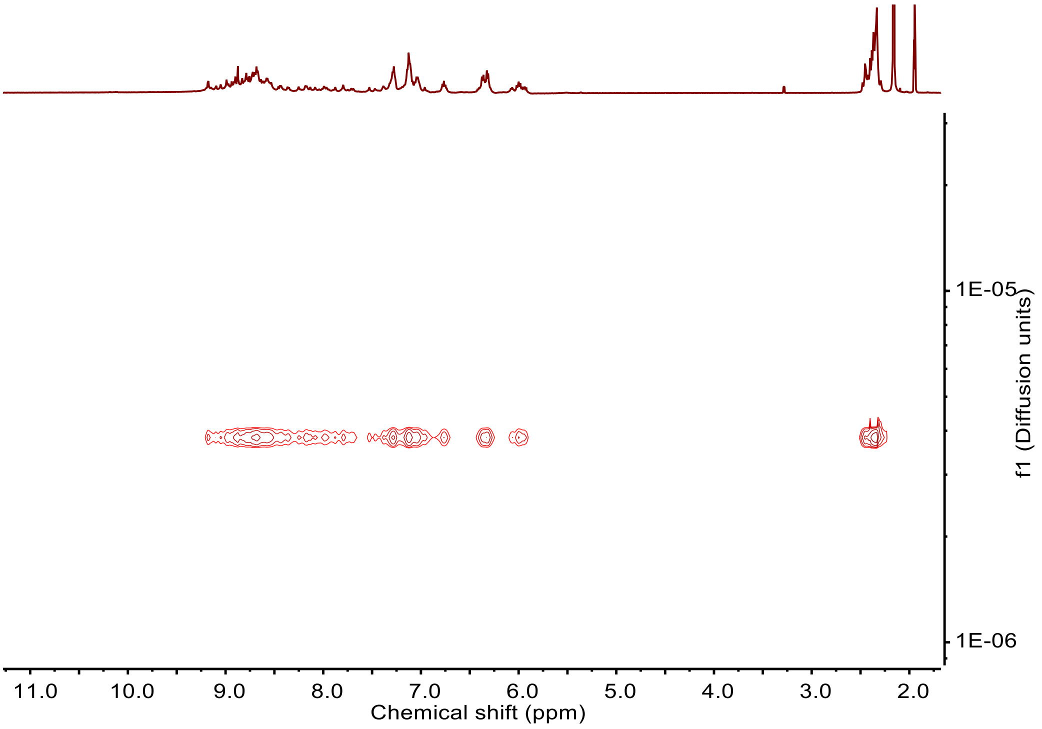

Figure S13. ^{1}H DOSY spectrum (400 MHz, 298 K, CD_3CN) of cage **1**. The diffusion coefficient was measured to be 3.8×10^{-6} cm^2 s^{-1}, and the hydrodynamic radius was calculated to be 17.1 Å according to the Stokes-Einstein equation.

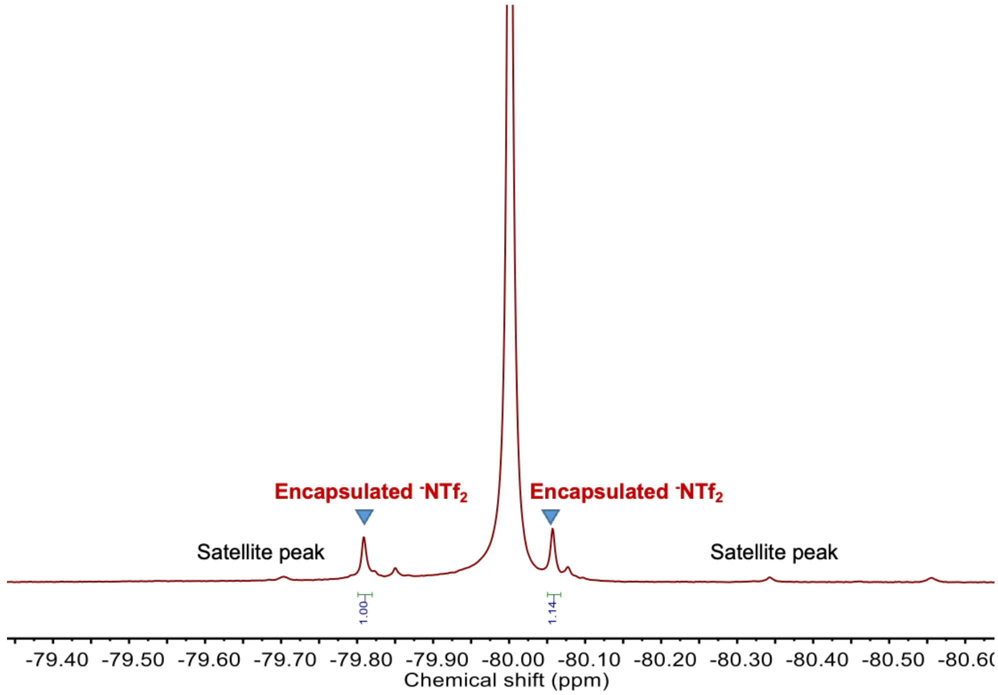

Figure S14. ^{19}F NMR spectrum (376 MHz, 298 K, CD_3CN) of cage **1**. The two encapsulated triflimide peaks are attributed to the counteranion bound within the two diastereomeric configurations shown in Main Text Figure 2.

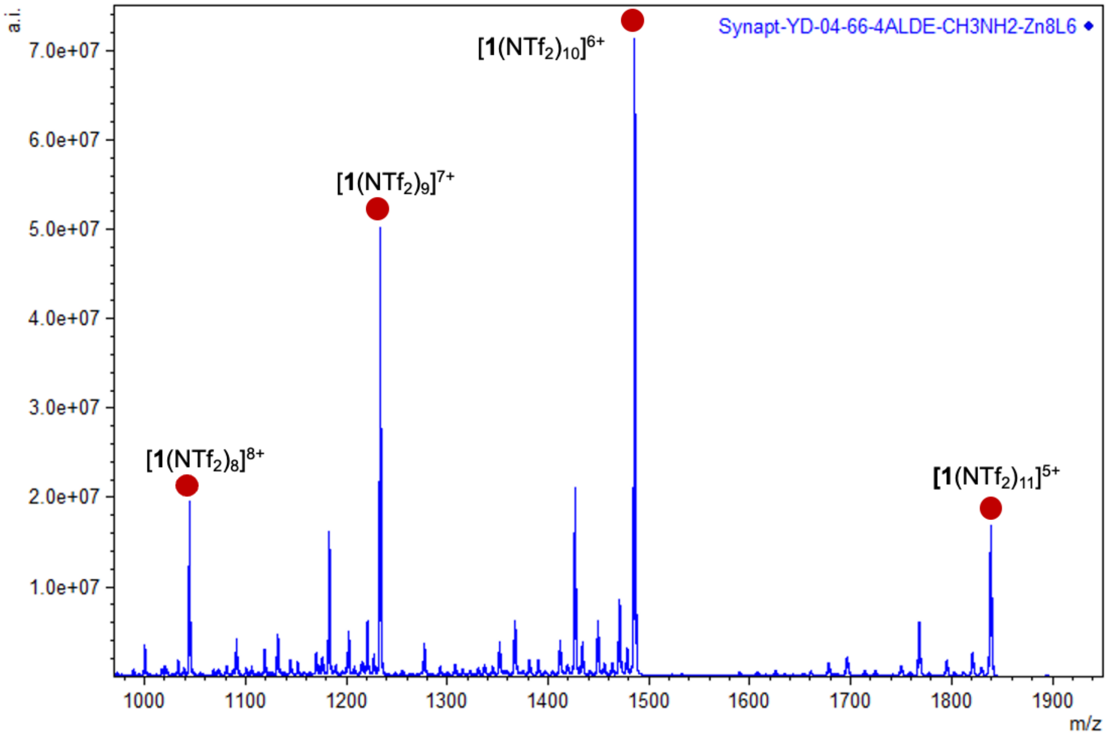

Figure S15. High-resolution ESI-mass spectrum of **1**·$[NTf_2]_{16}$. ESI-MS: Experimental results: *m/z* = 1043.65 [**1**$(NTf_2)_8]^{8+}$, 1233.02 [**1**$(NTf_2)_9]^{7+}$, 1485.17 [**1**$(NTf_2)_{10}]^{6+}$, 1838.18 [**1**$(NTf_2)_{11}]^{5}$. Calculated values: *m/z* = 1043.90 [**1**$(NTf_2)_8]^{8+}$, 1233.05 [**1**$(NTf_2)_9]^{7+}$, 1485.25 [**1**$(NTf_2)_{10}]^{6+}$, 1838.32 [**1**$(NTf_2)_{11}]^{5+}$.

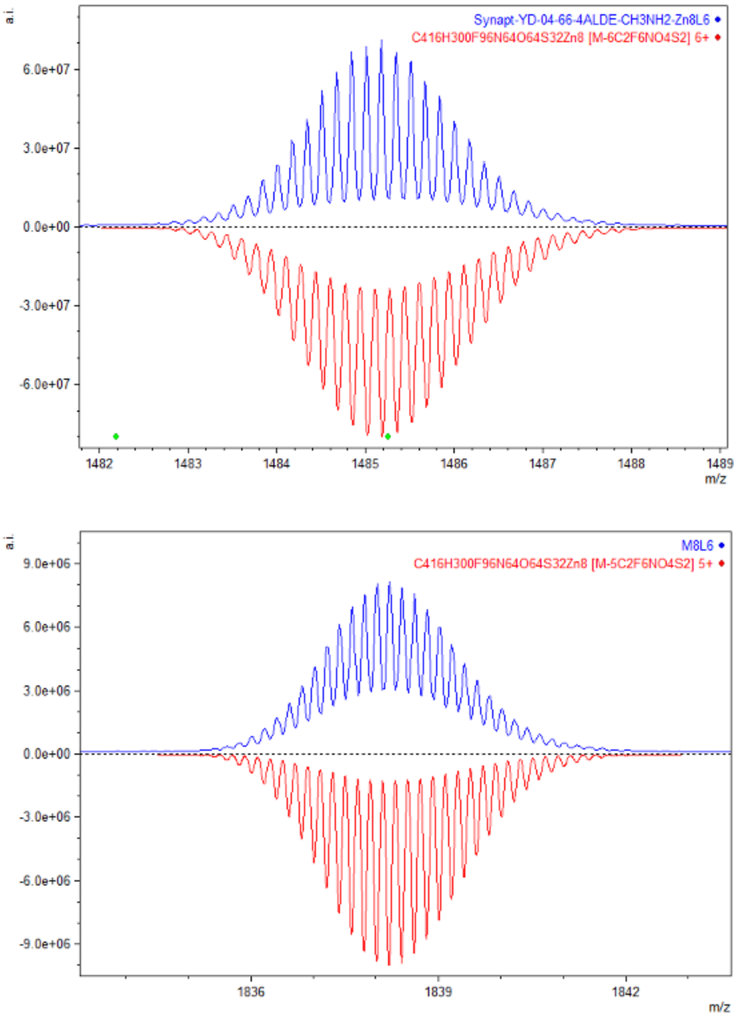

Figure S16. Signals from the high-resolution ESI-mass spectrum for **1**·$[NTf_2]_{16}$. Experimental (blue) and calculated (red) signals for $[\mathbf{1}(NTf_2)_{10}]^{6+}$ (top) and $[\mathbf{1}(NTf_2)_{11}]^{5+}$ (bottom).

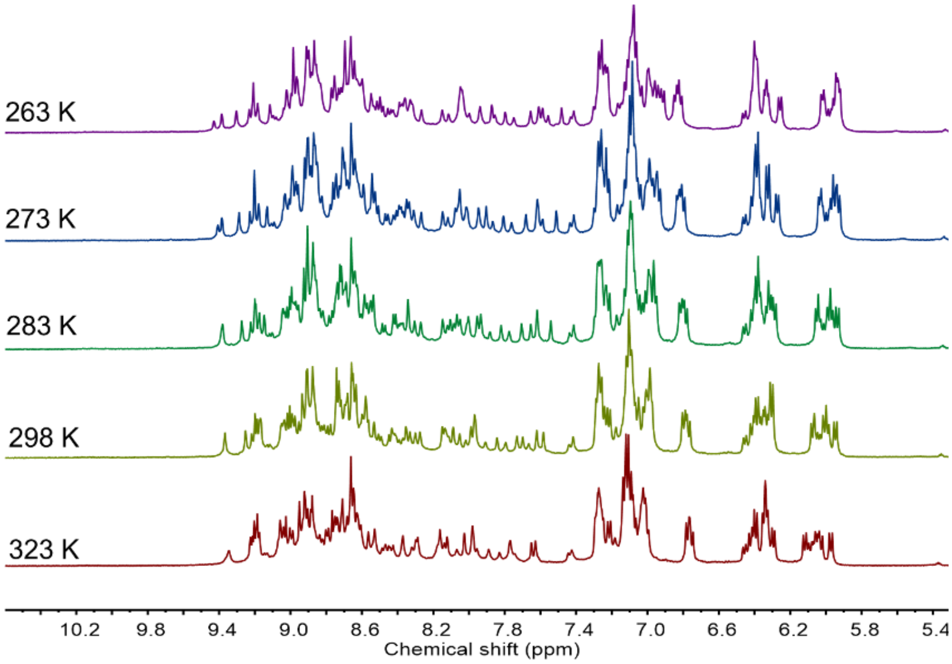

Figure S17. VT NMR (500 MHz, CD_3CN) of cage **1** from 263 K to 323 K.

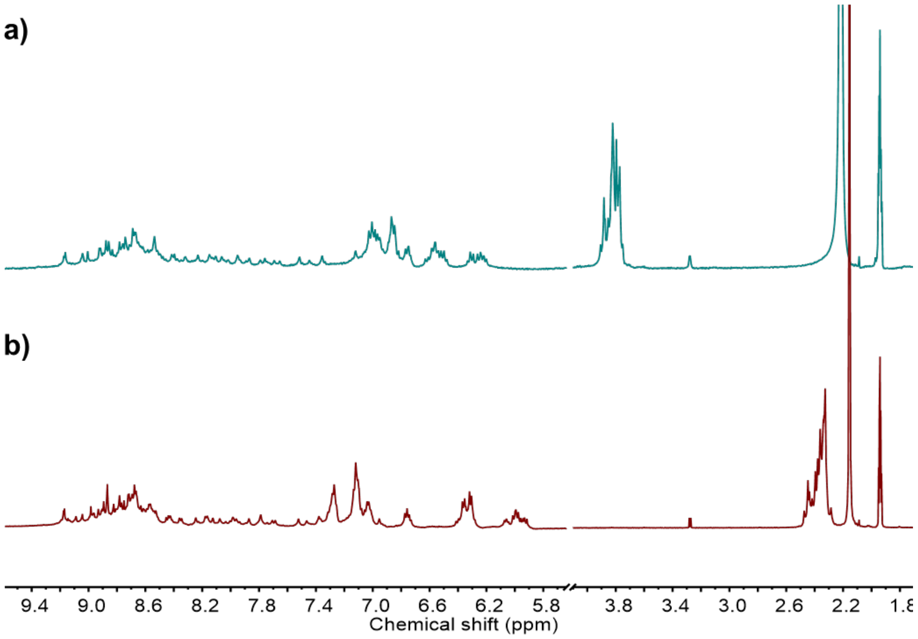

Figure S18. Comparison of ^{1}H NMR spectra (500 MHz, 298 K, CD_3CN) of **a)** cage **1'** through self-assembly of subcomponent **A**, *p*-anisidine and $Zn(NTf_2)_2$ and **b)** cage **1** through self-assembly of subcomponent **A**, *p*-toluidine and $Zn(NTf_2)_2$. Cage **1'** was synthesized via an identical synthetic procedure to that of **1** except that *p*-anisidine used in place of *p*-toluidine.

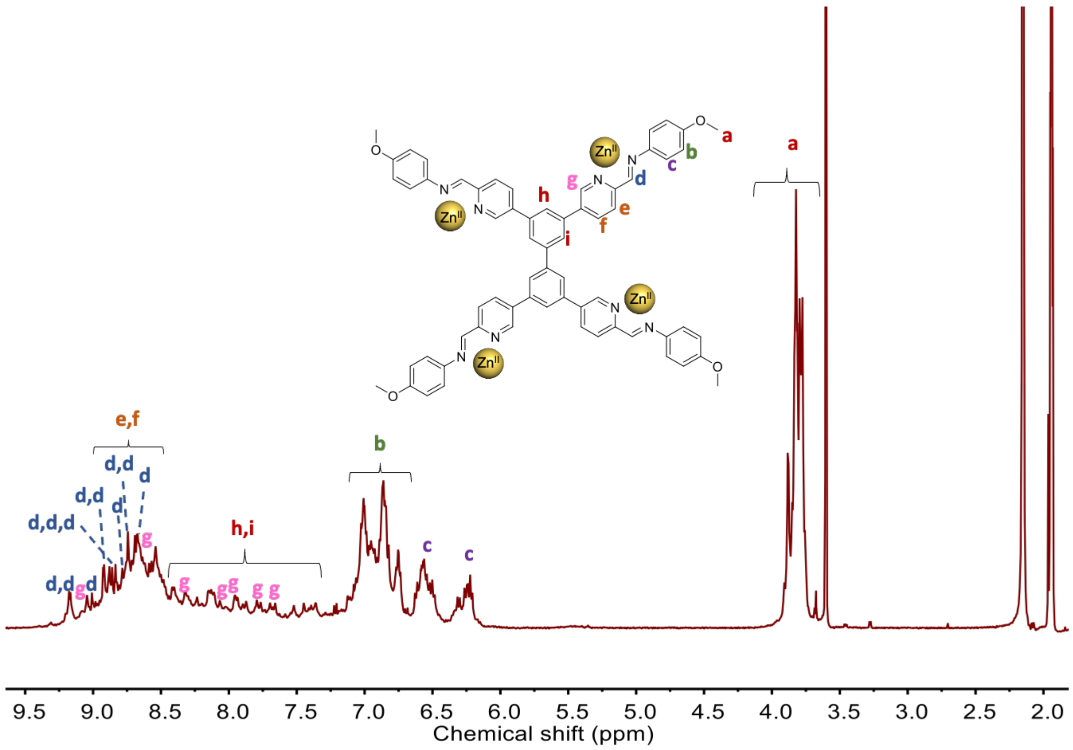

Figure S19. ^{1}H NMR spectrum (500 MHz, 298 K, CD_3CN) of cage **1’** with proton assignments shown.

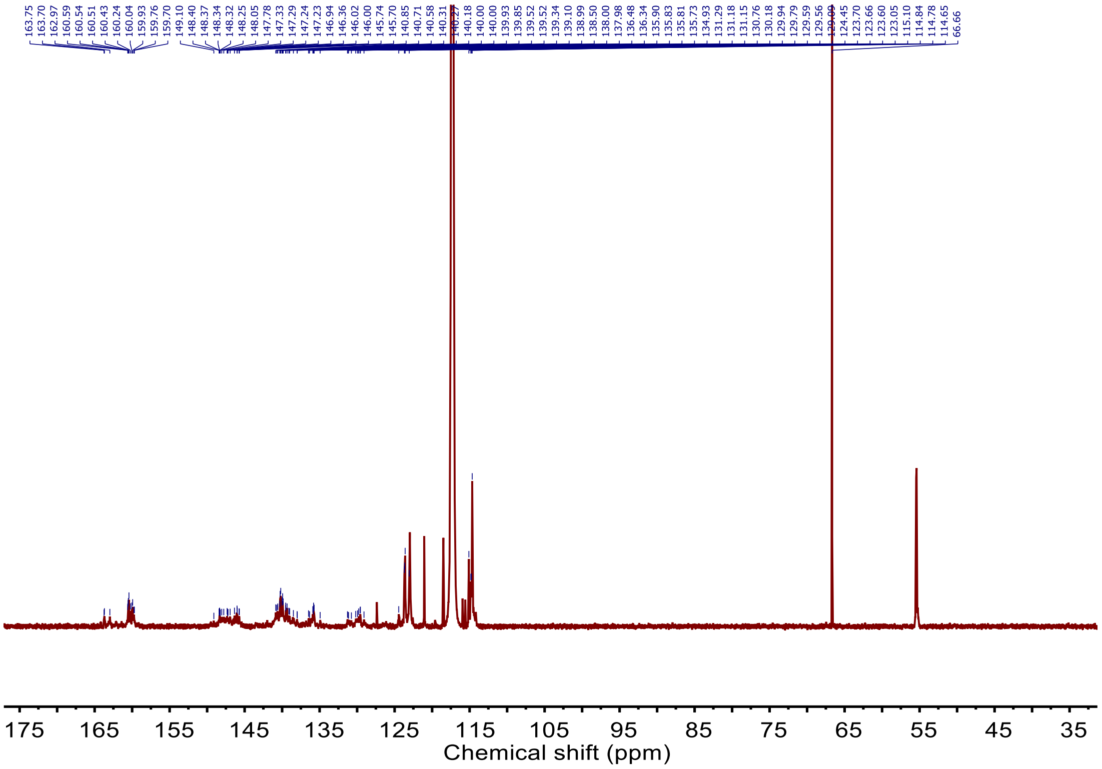

Figure S20. ^{13}C NMR spectrum (126 MHz, 298 K, CD_3CN) of cage **1’**.

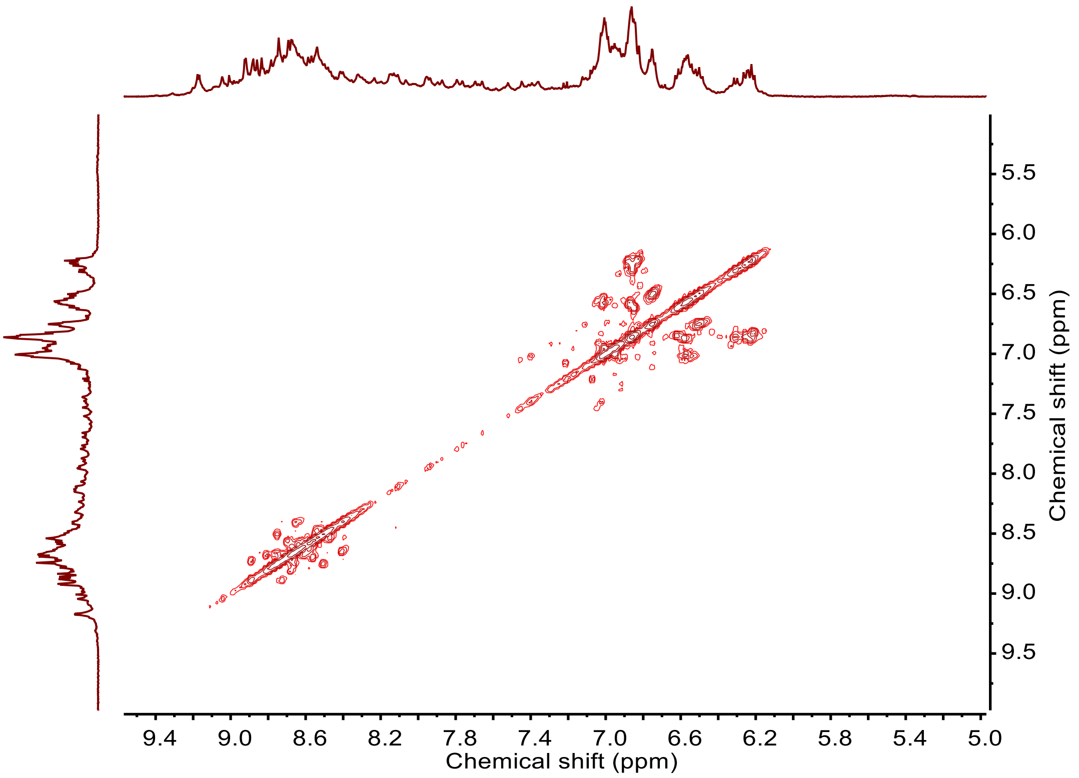

Figure S21. Partial ^{1}H-^{1}H COSY NMR spectrum (500 MHz, 298 K, CD_3CN) of cage **1'**.

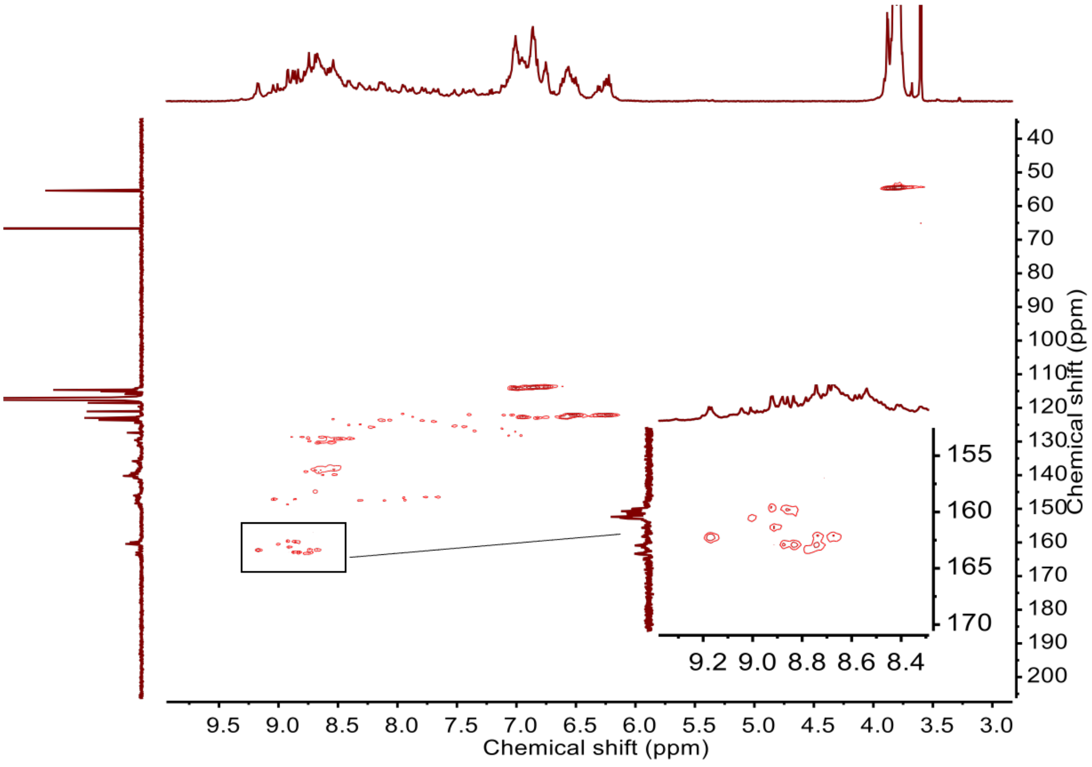

Figure S22. Partial ^{1}H-^{13}C HSQC NMR spectrum (500 MHz, 298 K, CD_3CN) of cage **1'**.

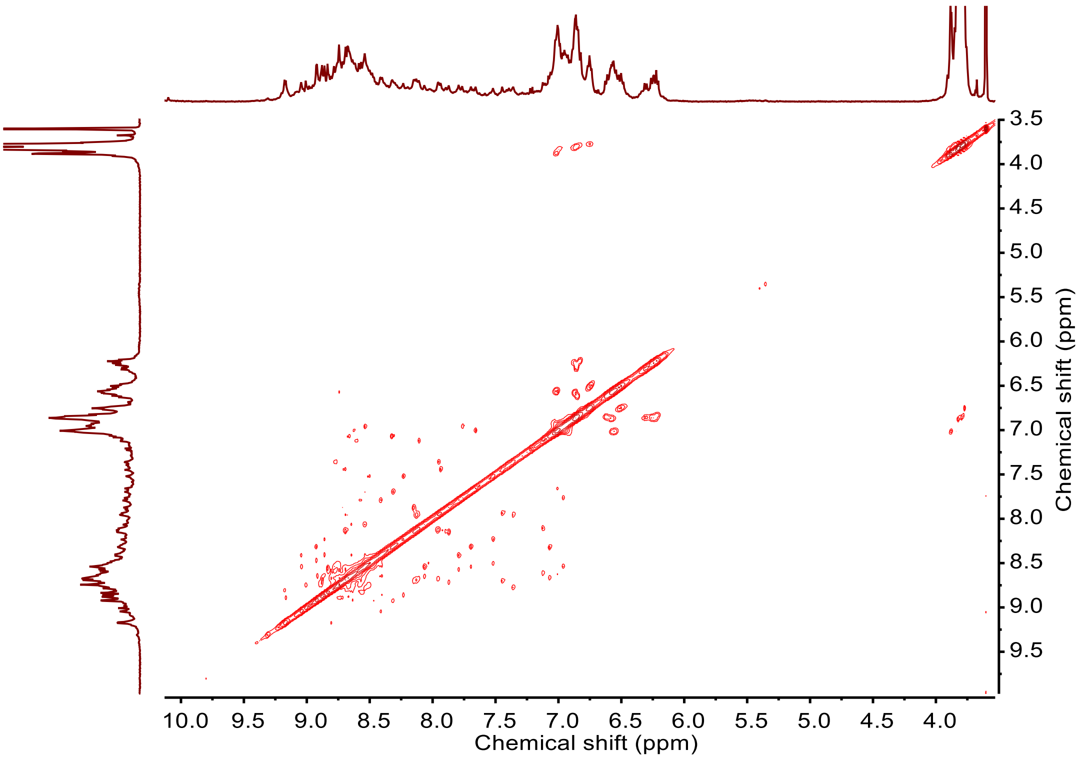

Figure S23. Partial ^{1}H-^{1}H NOESY NMR spectrum (500 MHz, 298 K, CD_3CN) of cage **1'**.

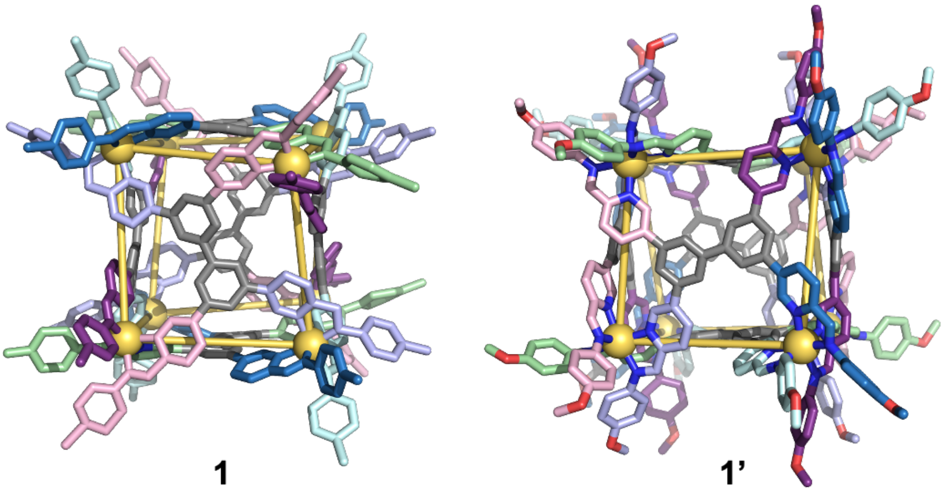

Figure S24. Illustration of magnetically distinct proton environments in cage **1** and **1'**, shown with different colors.

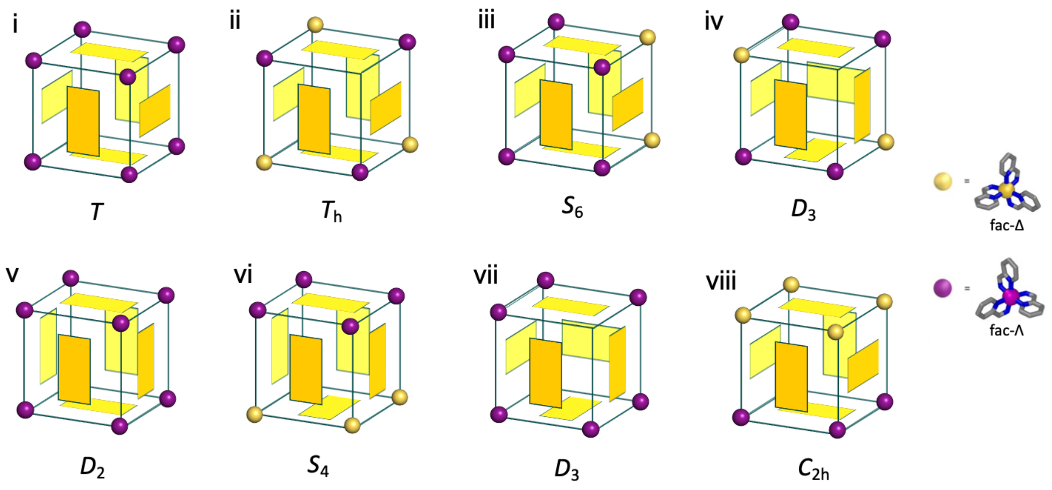

Figure S25. Illustration of the diastereomers of *pseudo*-cubes that have been observed experimentally so far. Diastereomers i–iv[3], v[4], vi[5], vii[6], viii[7] correspond to reported crystal structures.

2.4 Self-assembly and characterization of cage **2**

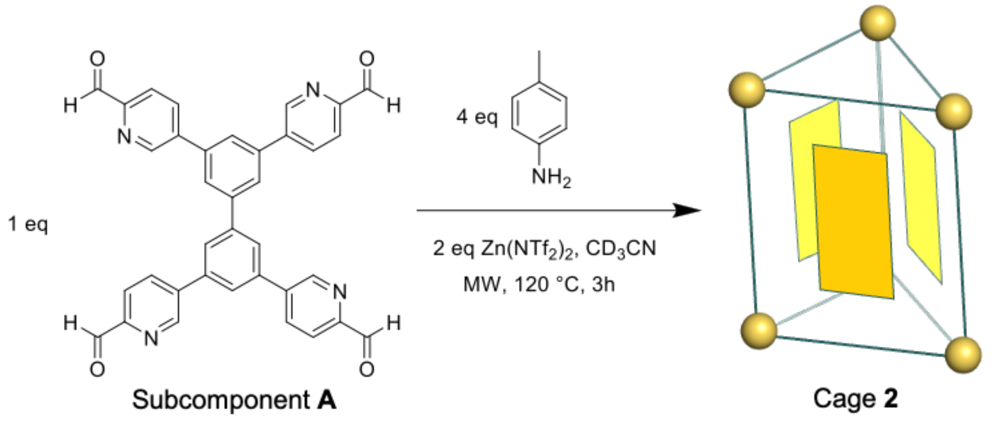

Scheme S4. Self-assembly of cage **2**.

Subcomponent **A** (10.0 mg, 17.4 μmol, 1 equiv.), *p*-toluidine (7.45 mg, 69.6 μmol, 4 equiv.), $Zn(NTf_2)_2$ (21.8 mg, 34.8 μmol, 2 equiv.) and CH_3CN (2 ml) were added to a vial, and the mixture was sonicated for 1 min. The mixture was then heated at 120 °C for 3 hours using a microwave reactor. The solvent was removed by blowing with N_2 and then Et_2O (10 ml) was added. The resulting solid was collected by centrifugation, washed three times with additional Et_2O (10 ml) and then dried under dynamic vacuum for 16 h at 298 K to give **2** as a yellow solid (29.8 mg, 4.55 μmol, yield 78.4 %).

^{1}H NMR (500 MHz, CD_3CN) δ 9.36 (s, 1H), 9.13 (s, 1H), 8.88 (s, 1H), 8.80 (d, J = 8.1 Hz, 1H), 8.72 (d, J = 8.1 Hz 1H), 8.66 (d, J = 8.1Hz, 1H), 8.29 (d, J = 8.1 Hz, 1H), 8.25 (s, 1H), 8.06 – 8.01 (d, 2H), 7.58 (s, 1H), 7.26 (m, 4H), 7.01 – 6.93 (m, 4H), 2.38 (d, J = 9.9 Hz, 6H).

^{13}C NMR (126 MHz, CD_3CN) δ 162.0, 162.0, 148.2, 147.4, 146.8, 145.2, 144.1, 143.9, 141.5, 141.2, 140.7, 139.5, 139.4, 137.6, 136.4, 130.5, 130.2, 130.0, 129.8, 126.5, 122.3, 121.9, 121.6, 119.8 (q, J = 229.7 Hz, $^-NTf_2$), 20.1, 20.1.

ESI-MS (CH_3CN) *m/z* = 655.20 $[\mathbf{2}(NTf_2)_5]^{7+}$, 811.05 $[\mathbf{2}(NTf_2)_6]^{6+}$, 1029.05 $[\mathbf{2}(NTf_2)_7]^{5+}$, 1356.54 $[\mathbf{2}(NTf_2)_8]^{4+}$, 1902.02 $[\mathbf{2}(NTf_2)_9]^{3+}$.

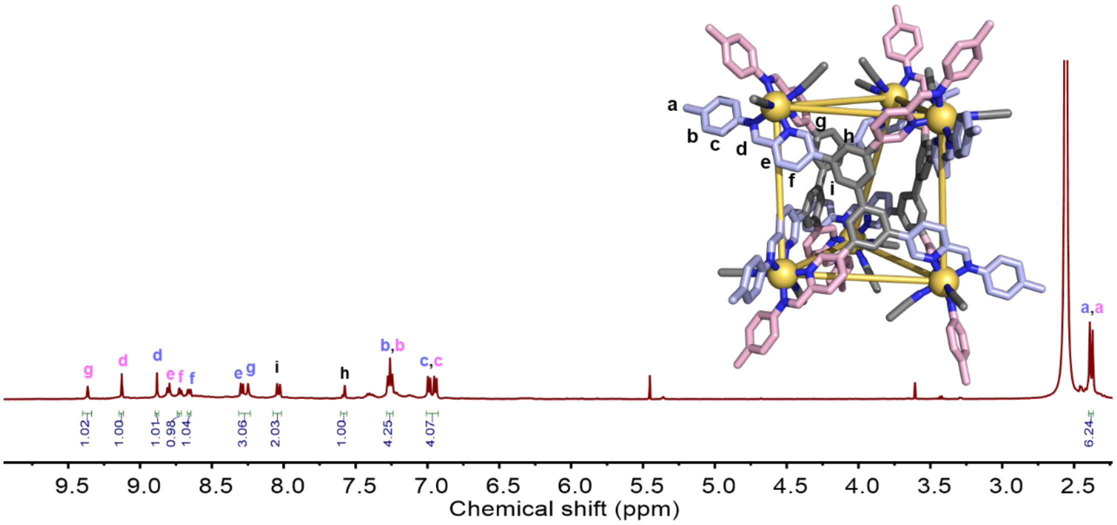

Figure S26. ^{1}H NMR spectrum (500 MHz, 298 K, CD_3CN) of cage **2**.

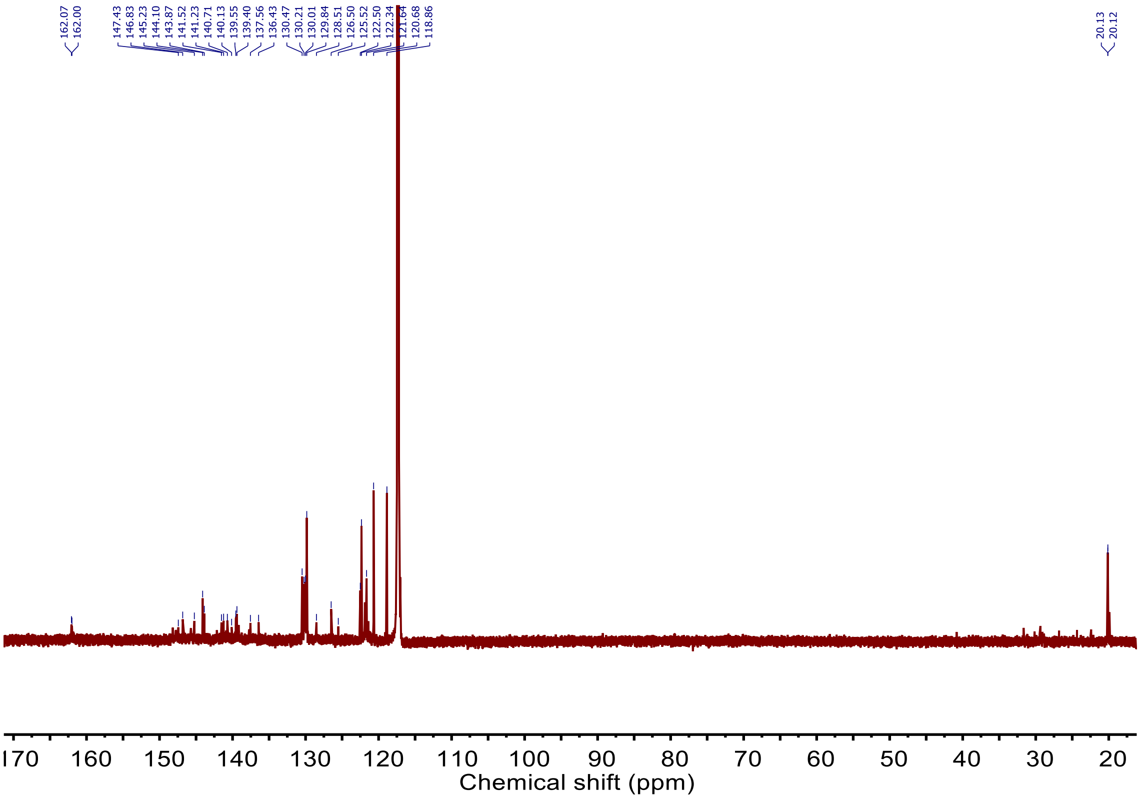

Figure S27. ^{13}C NMR spectrum (126 MHz, 298 K, CD_3CN) of cage **2**.

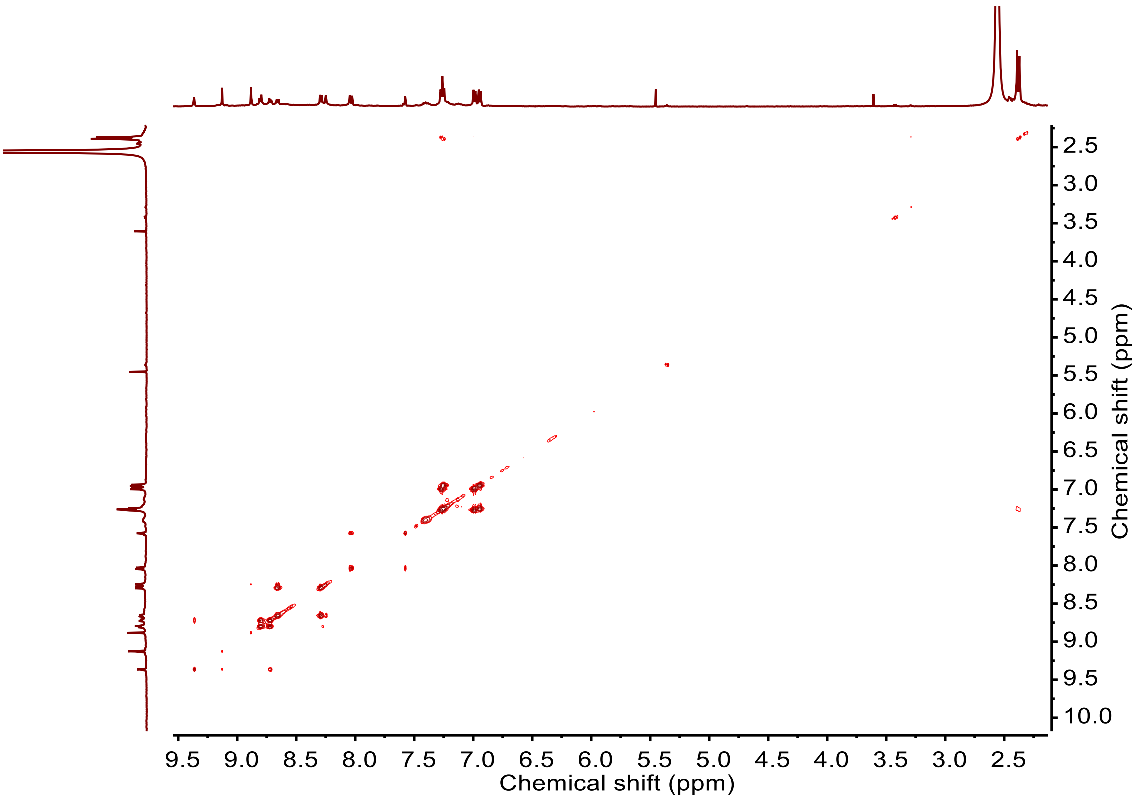

Figure S28. Partial ^{1}H-^{1}H COSY NMR spectrum (500 MHz, 298 K, CD_3CN) of cage **2**.

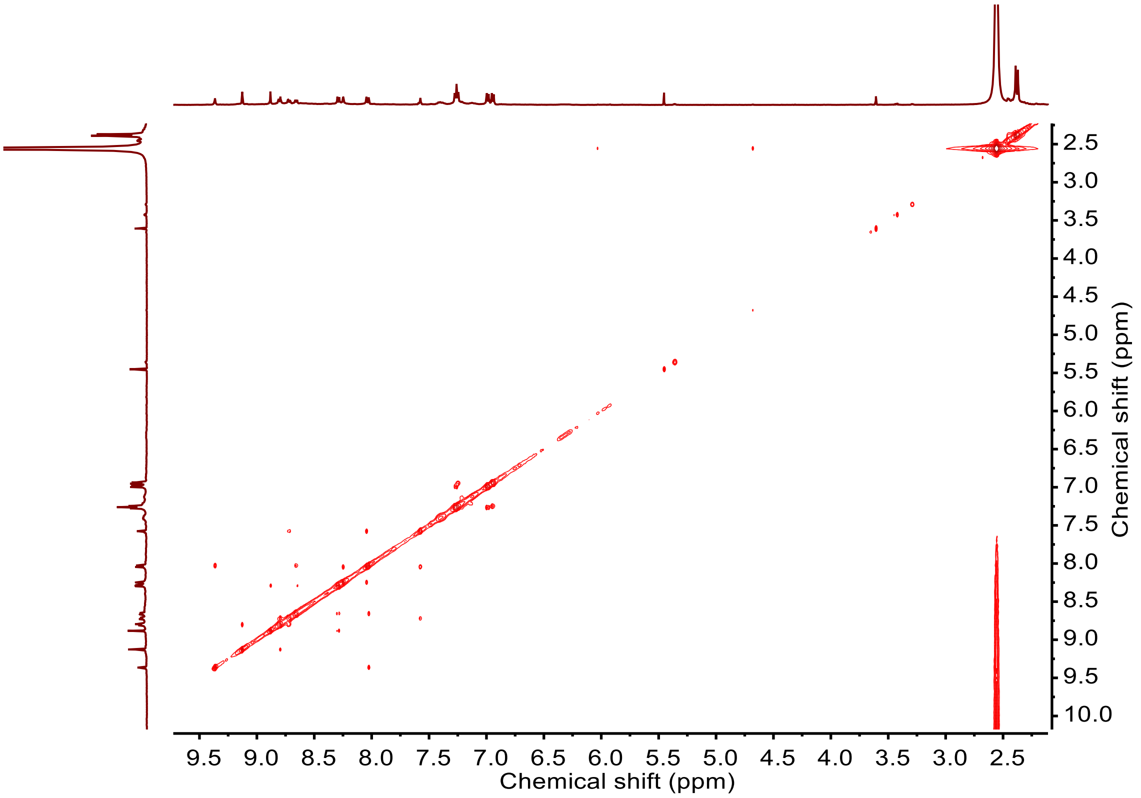

Figure S29. Partial ^{1}H-^{1}H NOESY NMR spectrum (500 MHz, 298 K, CD_3CN) of cage **2**.

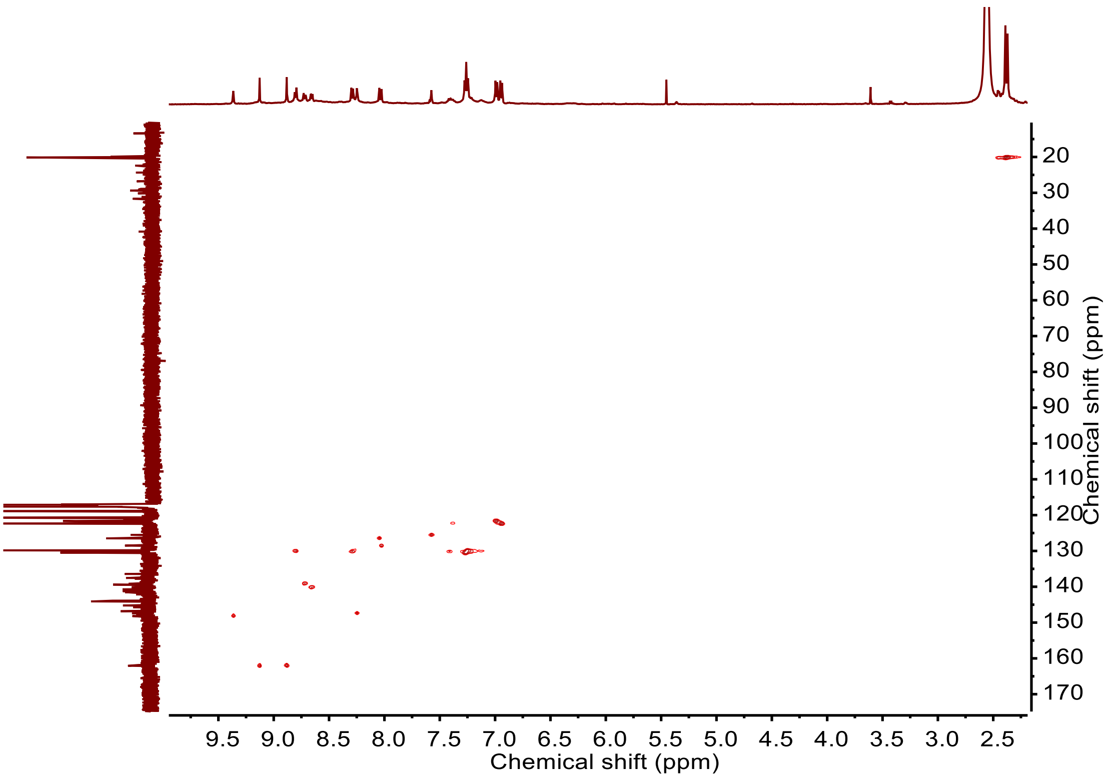

Figure S30. Partial ^{1}H-^{13}C HSQC NMR spectrum (500 MHz, 298 K, CD_3CN) of cage **2**.

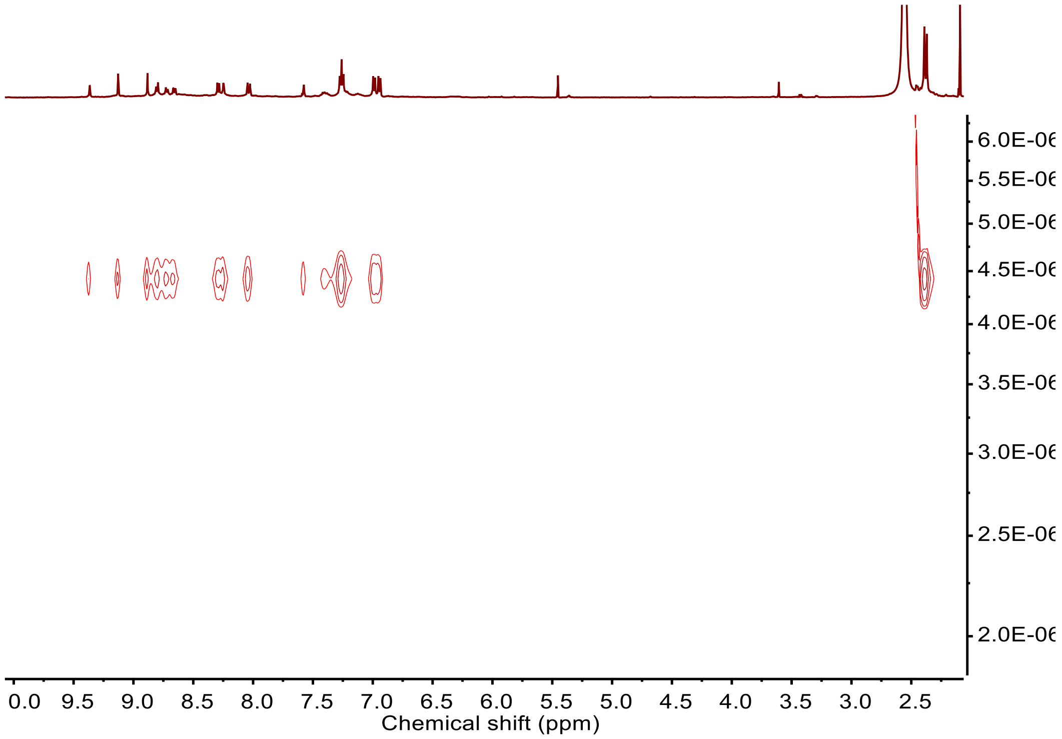

Figure S31. ^{1}H DOSY spectrum (400 MHz, 298 K, CD_3CN) of cage **2**. The diffusion coefficient was measured to be 4.4×10^{-6} cm^2 s^{-1}, and the hydrodynamic radius was calculated to be 14.8 Å according to the Stokes-Einstein equation.

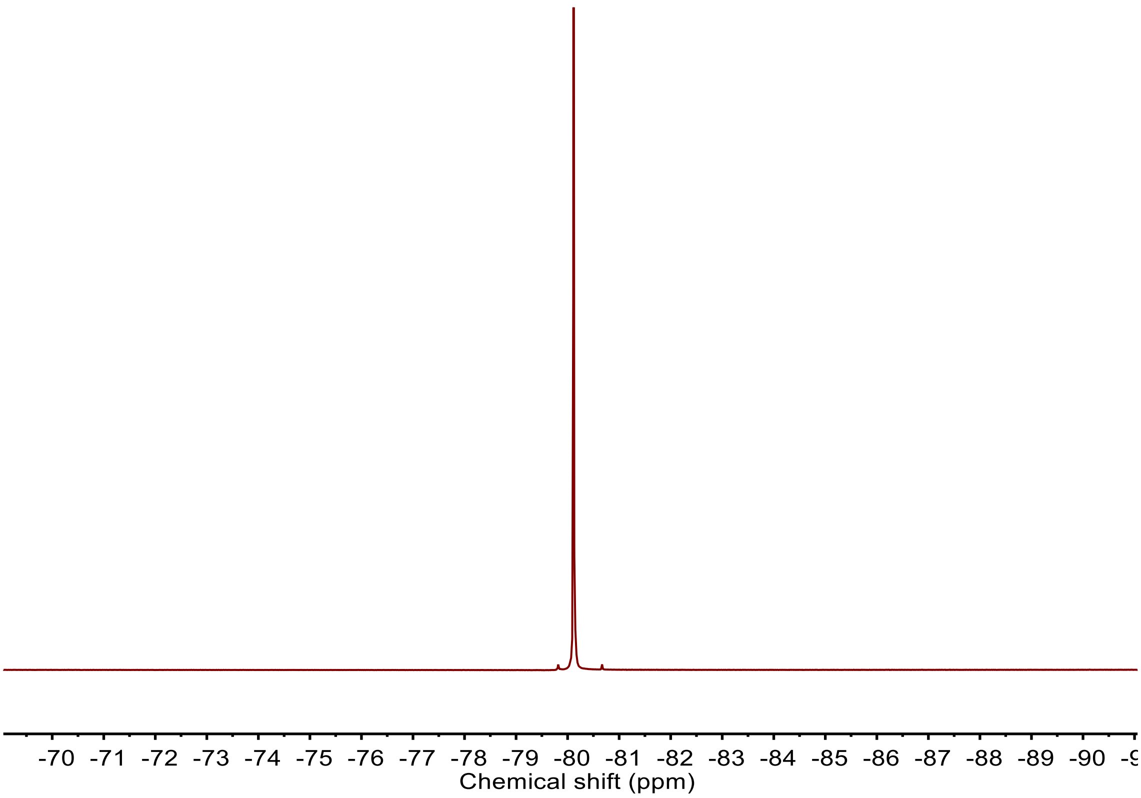

Figure S32. ^{19}F NMR spectrum (376 MHz, 298 K, CD_3CN) of cage **2**.

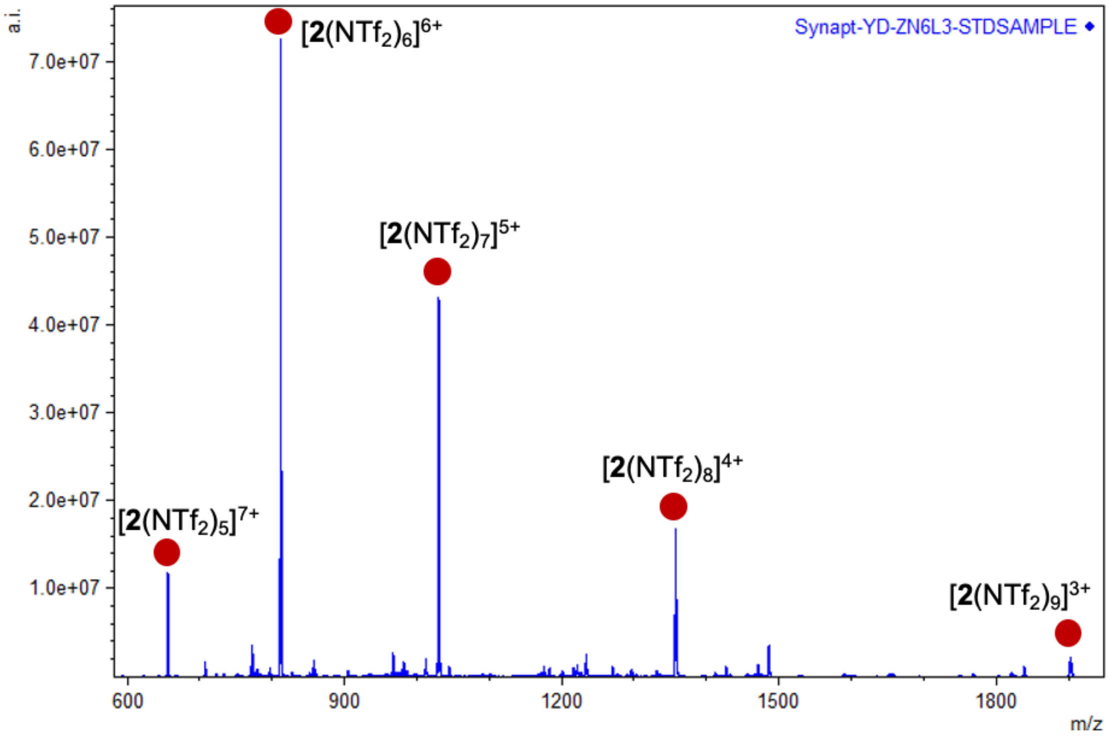

Figure S33. High-resolution ESI-mass spectrum of **2**·$[NTf_2]_{12}$. ESI-MS: Experimental results: *m/z* = 655.20 $[\mathbf{2}(NTf_2)_5]^{7+}$, 811.05 $[\mathbf{2}(NTf_2)_6]^{6+}$, 1029.05 $[\mathbf{2}(NTf_2)_7]^{5+}$, 1356.54 $[\mathbf{2}(NTf_2)_8]^{4+}$, 1902.02 $[\mathbf{2}(NTf_2)_9]^{3+}$. Calculated values: *m/z* = 655.22 $[\mathbf{2}(NTf_2)_5]^{7+}$, 811.11 $[\mathbf{2}(NTf_2)_6]^{6+}$, 1029.36 $[\mathbf{2}(NTf_2)_7]^{5+}$, 1356.74 $[\mathbf{2}(NTf_2)_8]^{4+}$, 1902.37 $[\mathbf{2}(NTf_2)_9]^{3+}$.

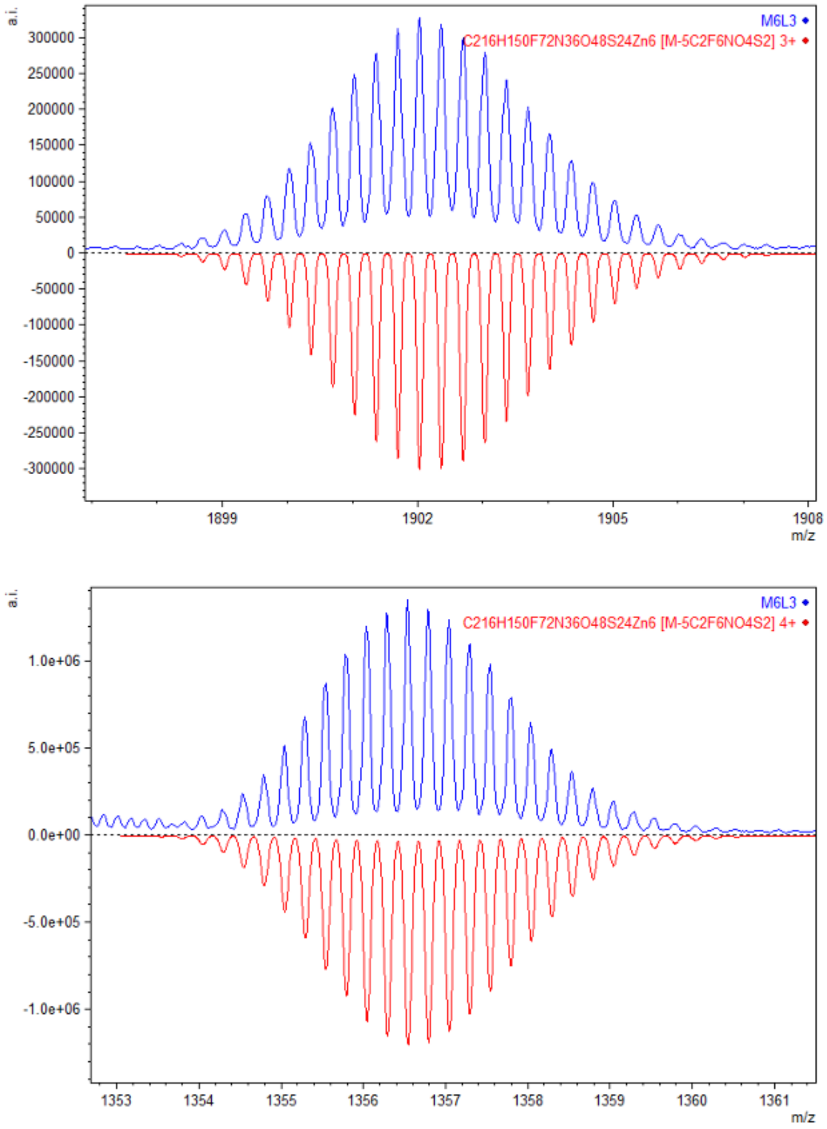

Figure S34. Signals from the high-resolution ESI-mass spectrum for **2**·[NTf$_2$]$_{12}$. Experimental (blue) and calculated (red) signals for [**2**(NTf$_2$)$_9$]$^{3+}$ (top) and [**2**(NTf$_2$)$_8$]$^{4+}$ (bottom).

2.5 Self-assembly and characterization of cage **3**

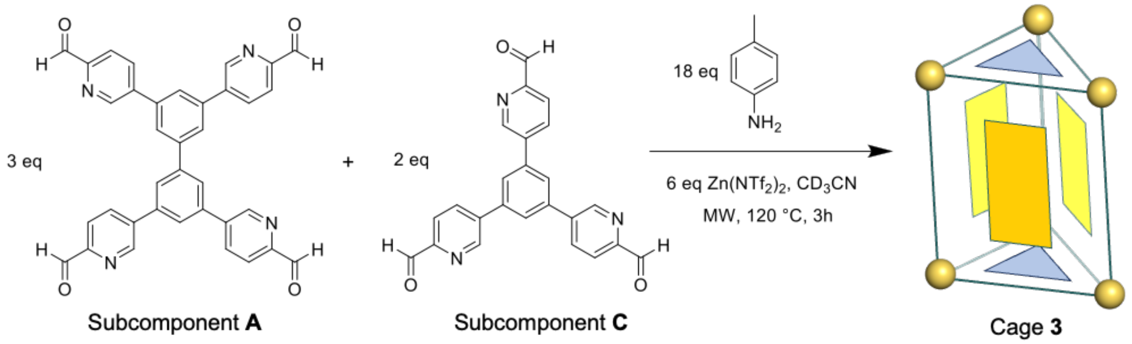

Scheme S5. Self-assembly of cage **3**.

Subcomponent **A** (10.0 mg, 17.4 μmol, 3 equiv.), subcomponent **C** (4.5 mg, 11.4 μmol, 2 equiv.), *p*-toluidine (11.1 mg, 103.6 μmol, 18 equiv.), $Zn(NTf_2)_2$ (21.8 mg, 34.8 μmol, 6 equiv.) and CH_3CN (2 ml) were added to a vial, and the mixture was sonicated for 1 min. The mixture was then heated at 120 °C for 3 hours using a microwave reactor. The solvent was removed by blowing with N_2 and then Et_2O (10 ml) was added. The resulting solid was collected by centrifugation, washed three times with additional Et_2O (10 ml) and then dried under dynamic vacuum for 16 h at 298 K to give **3** as a yellow solid (36.7 mg, 4.66 μmol, yield 80.4 %).

^{1}H NMR (400 MHz, CD_3CN) δ 9.01 (s, 1H), 8.97 (s, 1H), 8.73 – 8.51 (m, 7H), 8.37 (s, 1H), 7.98 (s, 1H), 7.69 (s, 2H), 7.62 (s, 1H), 7.30 (m, 3H), 7.10 (m, 4H), 6.96 (s, 0.5H), 6.67 (d, J = 8.1Hz, 2H), 6.40 (d, J = 8.1Hz, 2H), 6.12 (d, J = 7.9 Hz, 2H), 2.41 (s, 3H), 2.39 (s, 3H), 2.33 (s, 3H).
^{13}C NMR (126 MHz, CD_3CN) δ 164.7, 163.2, 162.9, 147.3, 146.9, 146.2, 146.2, 146.0, 146.0, 145.9, 144.9, 144.3, 144.1, 141.6, 141.1, 140.1, 139.8, 139.2, 139.1, 139.1, 138.8, 138.2, 136.5, 136.3, 135.6, 132.0, 131.6, 130.4, 130.2, 130.1, 130.0, 126.5, 122.1, 121.7, 121.5, 121.3, 119.8 (q, J = 321.7 Hz, $^-NTf_2$), 20.2, 20.1, 20.0.
ESI-MS (CH_3CN) m/z = 844.18 $[\mathbf{3}(NTf_2)_5]^{7+}$, 1031.62 $[\mathbf{3}(NTf_2)_6]^{6+}$, 1293.98 $[\mathbf{3}(NTf_2)_7]^{5+}$, 1687.58 $[\mathbf{3}(NTf_2)_8]^{4+}$.

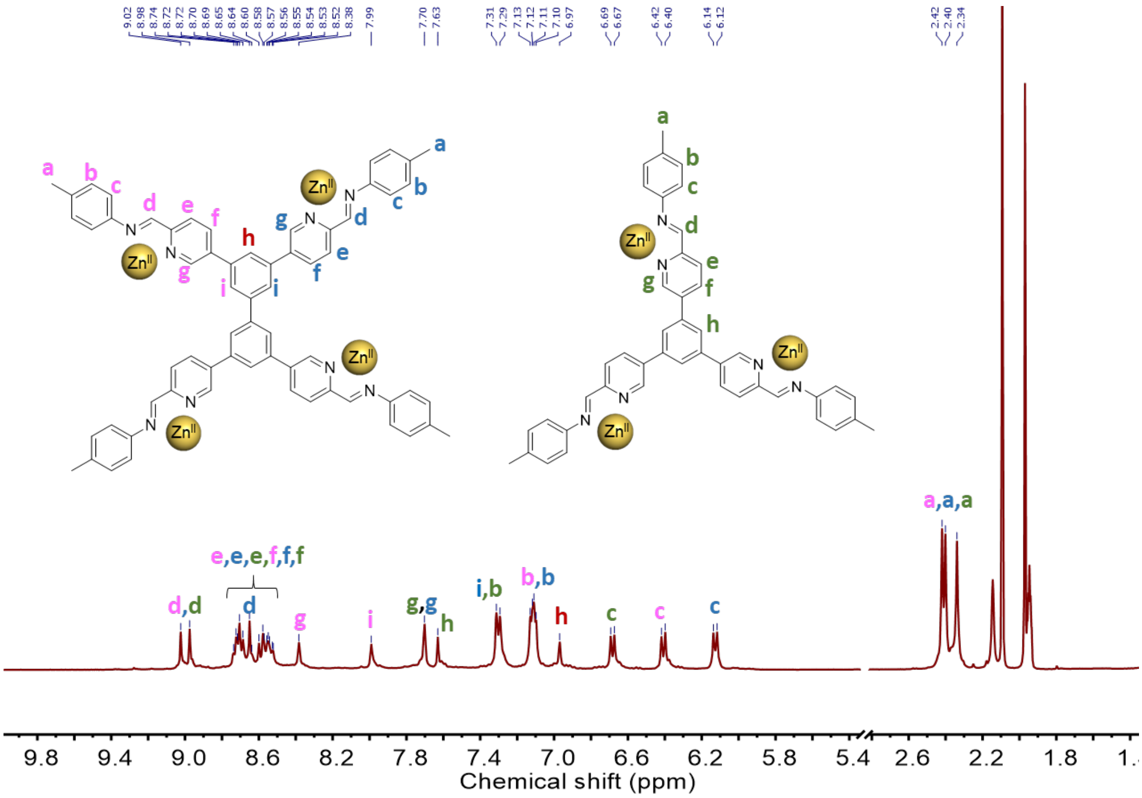

Figure S35. ^{1}H NMR spectrum (500 MHz, 298 K, CD_3CN) of cage **3**.

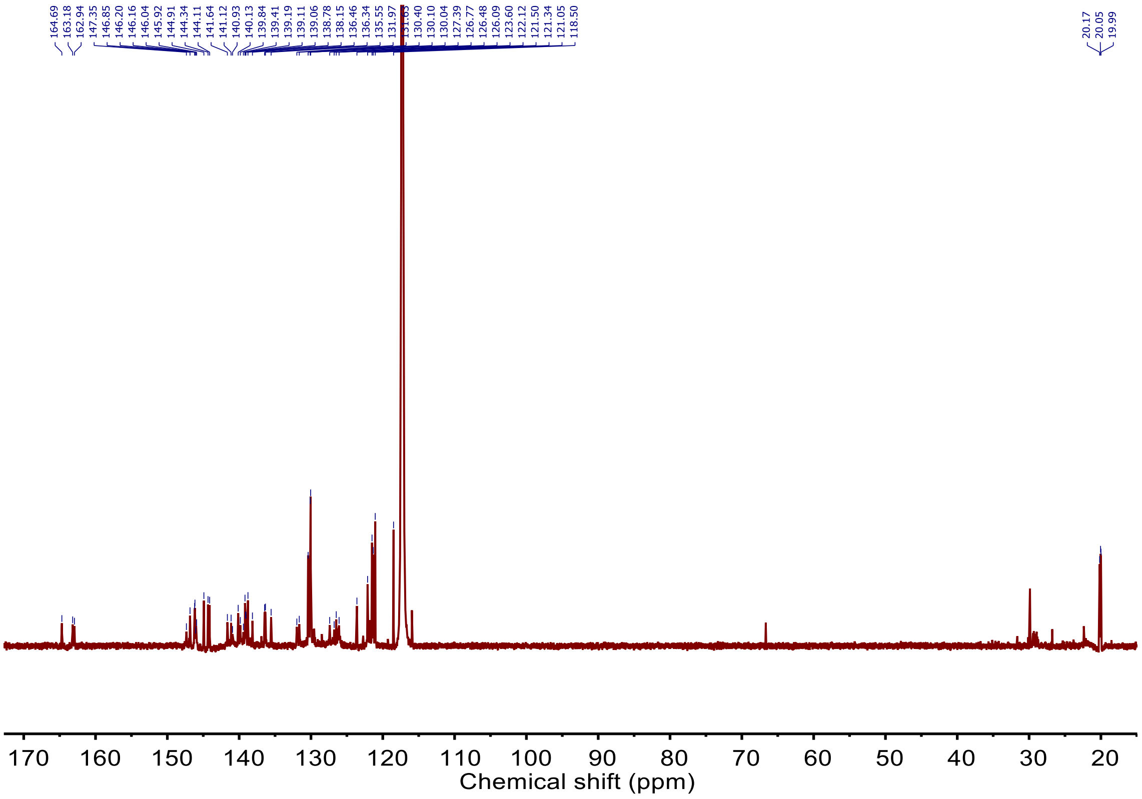

Figure S36. ^{13}C NMR spectrum (126 MHz, 298 K, CD_3CN) of cage **3**.

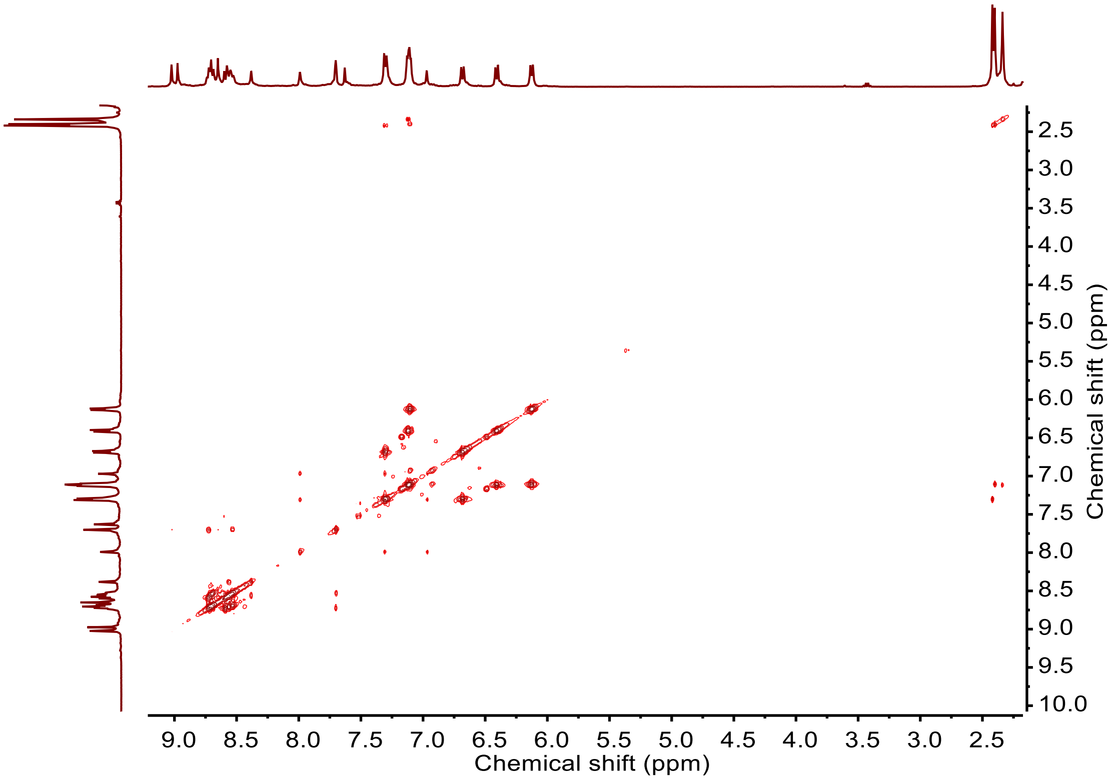

Figure S37. Partial ^{1}H-^{1}H COSY NMR spectrum (500 MHz, 298 K, CD_3CN) of cage **3**.

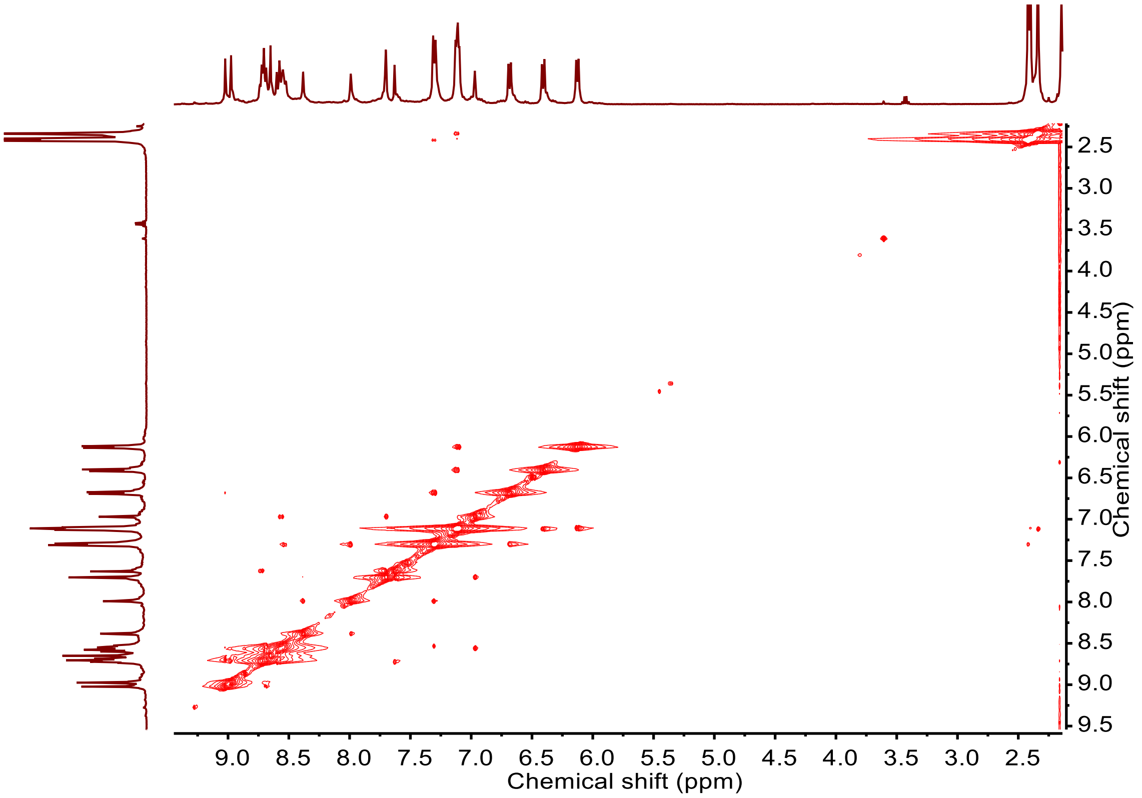

Figure S38. Partial ^{1}H-^{1}H NOESY NMR spectrum (500 MHz, 298 K, CD_3CN) of cage **3**.

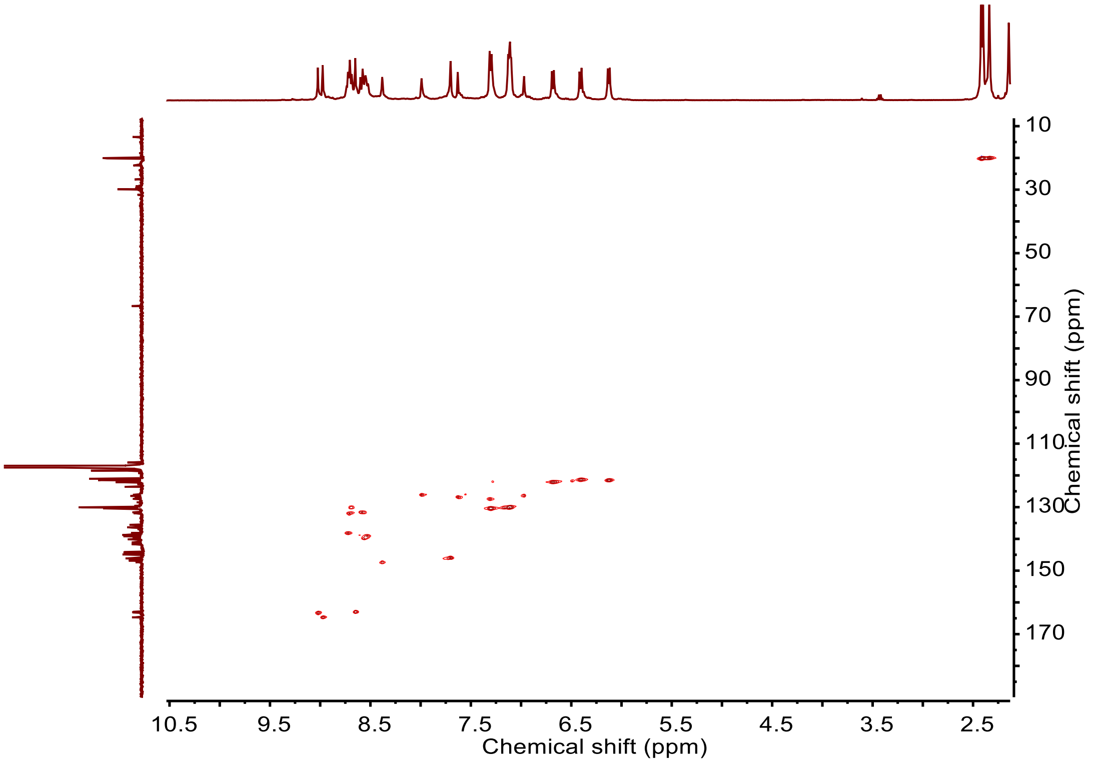

Figure S39. Partial ^{1}H-^{13}C HSQC NMR spectrum (500 MHz, 298 K, CD_3CN) of cage **3**.

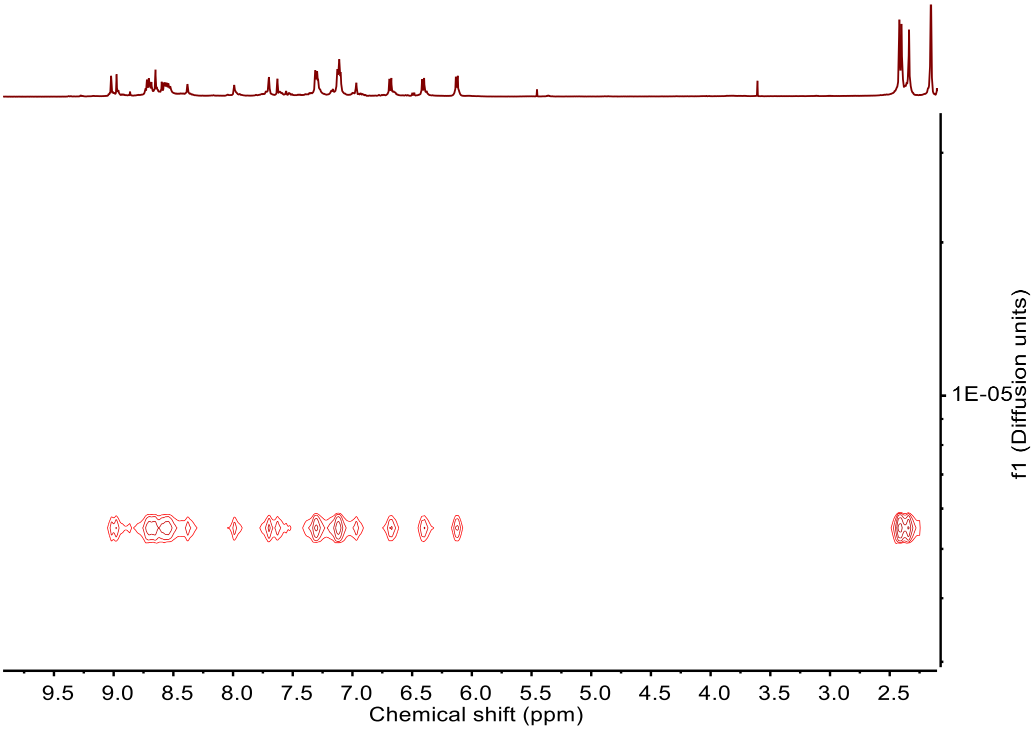

Figure S40. [1]H DOSY spectrum (400 MHz, 298 K, CD_3CN) of cage **3**. The diffusion coefficient was measured to be 4.4 × 10^{-6} cm^2 s^{-1}, and the hydrodynamic radius was calculated to be 14.8 Å according to the Stokes-Einstein equation.

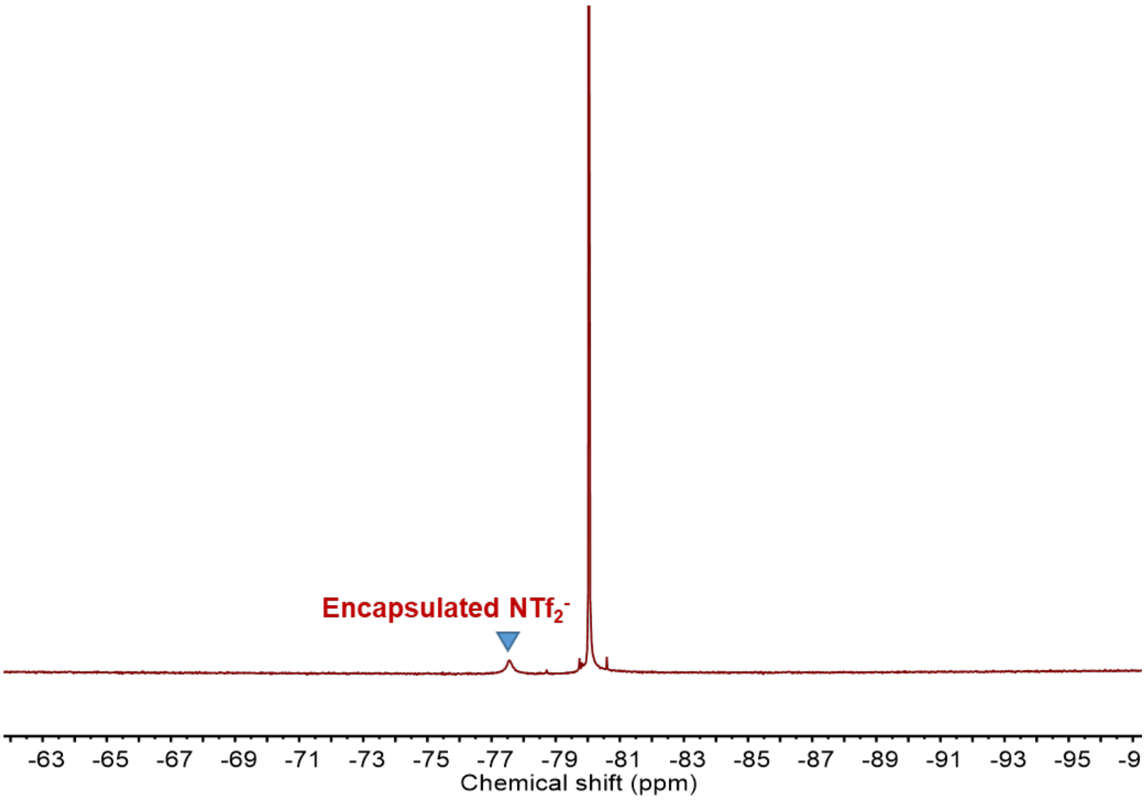

Figure S41. ^{19}F NMR spectrum (376 MHz, 298 K, CD_3CN) of cage **3**.

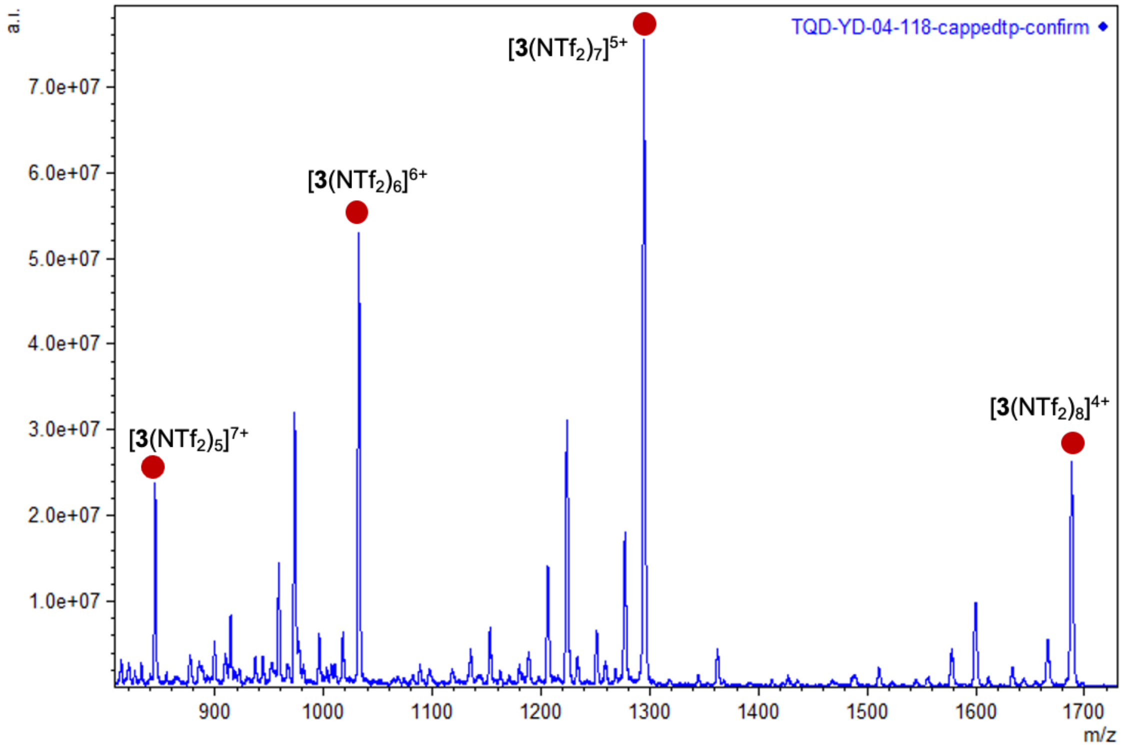

Figure S42. High-resolution ESI-mass spectrum of **3**·[NTf$_2$]$_{12}$. ESI-MS: Experimental results: *m/z* = 844.18 [**3**(NTf$_2$)$_5$]$^{7+}$, 1031.62 [**3**(NTf$_2$)$_6$]$^{6+}$, 1293.98 [**3**(NTf$_2$)$_7$]$^{5+}$, 1687.58 [**3**(NTf$_2$)$_8$]$^{4+}$. Calculated values: *m/z* = 844.18 [**3**(NTf$_2$)$_5$]$^{7+}$, 1031.62 [**3**(NTf$_2$)$_6$]$^{6+}$, 1293.98 [**3**(NTf$_2$)$_7$]$^{5+}$, 1687.58 [**3**(NTf$_2$)$_8$]$^{4+}$.

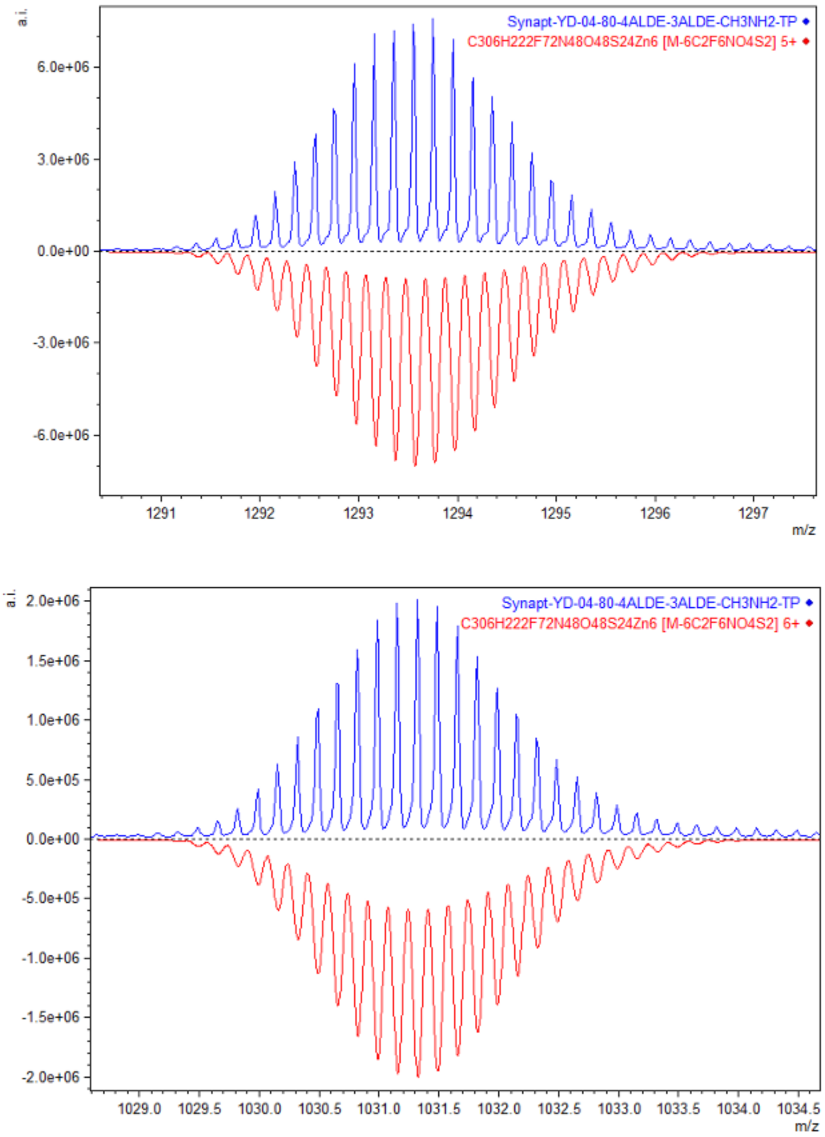

Figure S43. Signals from the high-resolution ESI-mass spectrum for **3**·$[NTf_2]_{12}$. Experimental (blue) and calculated (red) signals for $[\mathbf{3}(NTf_2)_7]^{5+}$ (top) and $[\mathbf{3}(NTf_2)_6]^{6+}$ (bottom).

3. Structural transformations

3.1 Structural transformation from cage **1** to cage **2**

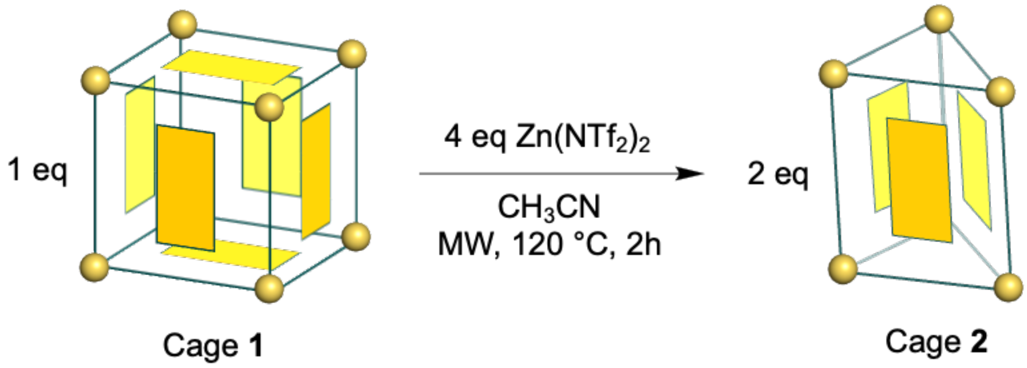

Scheme S6. Cage **1** (3.0 mg, 0.28 μmol, 1 equiv) and $Zn(NTf_2)_2$ (0.7 mg, 1.12 μmol, 4 equiv) were mixed in CD_3CN (0.5 mL) in a microwave react tube. The reaction mixture was stirred at 120 °C for 2 h in a microwave reactor. After cooling to room temperature, cage **2** was formed.

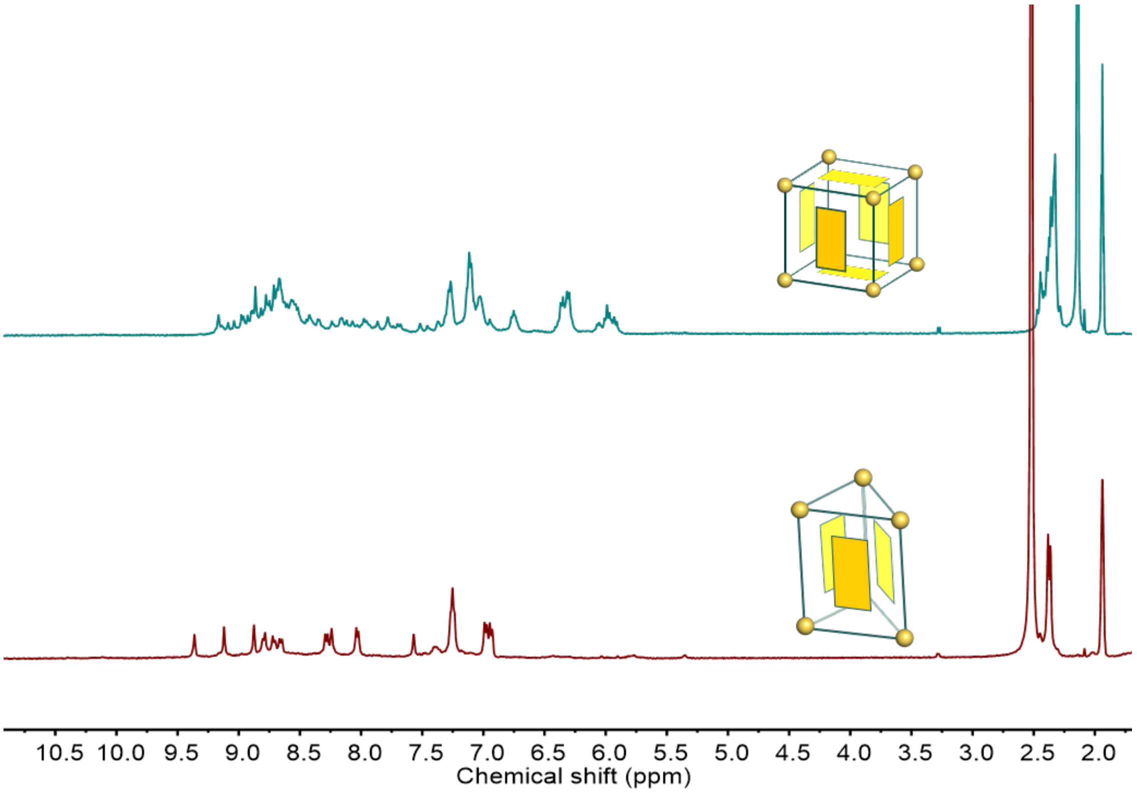

Figure S44. Comparison of ^{1}H NMR spectra (500 MHz, 298 K, CD_3CN) of cage **1** (top spectrum, green) and cage **2** formed from mixing cage **1** and $Zn(NTf_2)_2$ (bottom spectrum, red).

3.2 Structural transformation from cage **2** to cage **3**

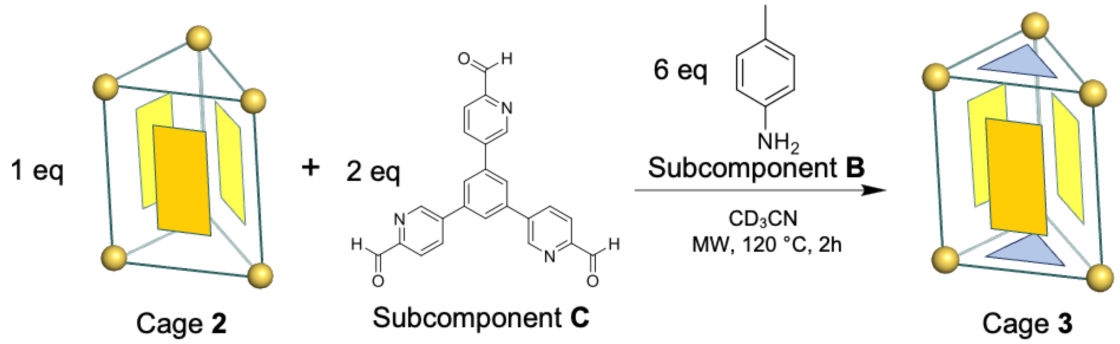

Scheme S7. Cage **2** (2.0 mg, 0.31 µmol, 1 equiv), Subcomponent **C** (0.24 mg, 0.61 µmol, 2 equiv) and Subcomponent **B** (0.20 mg, 1.83 µmol, 6 equiv) were mixed in CD_3CN (0.5 mL) in a microwave react tube. The reaction mixture was stirred at 120 °C for 2 h in a microwave reactor. After cooling to room temperature, cage **3** was formed.

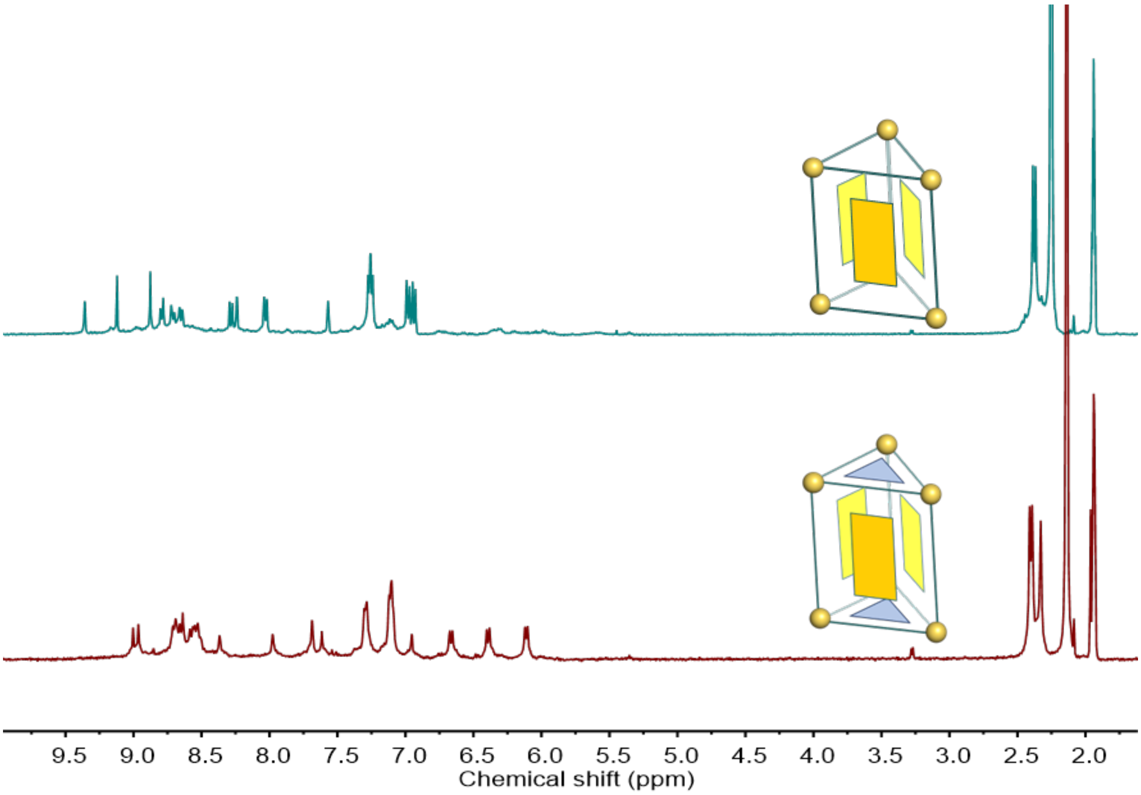

Figure S45. Comparison of ^1H NMR spectra (500 MHz, 298 K, CD_3CN) of cage **2** (top spectrum, green) and cage **3** formed from mixing cage **2**, subcomponent **B** and **C** (bottom spectrum, red).

3.3 Structural transformation from cage **1** and cage **4** to cage **3**

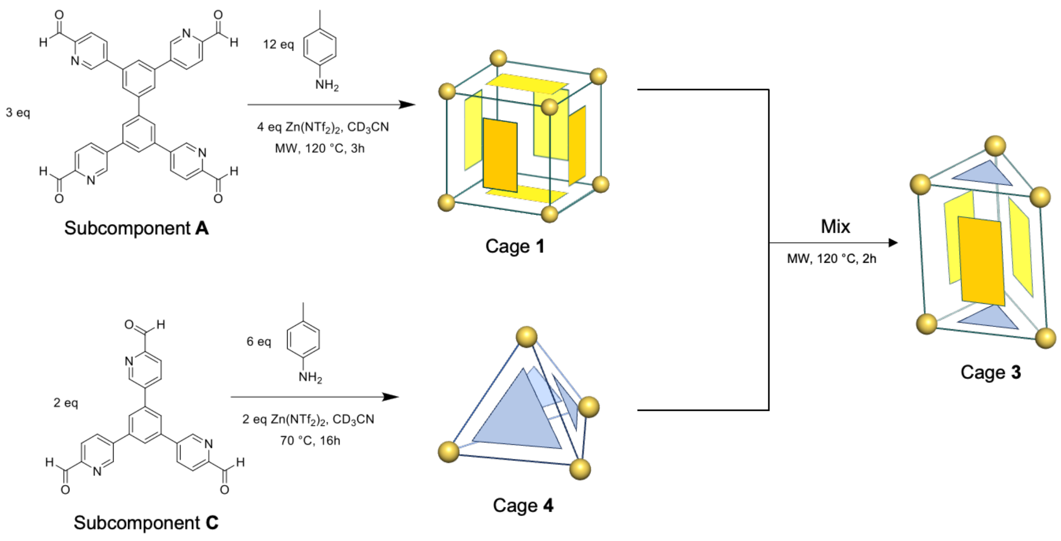

Scheme S8. Cage **1** (3.0 mg, 0.28 μmol, 1 equiv) and cage **4** (1.4 mg, 0.28 μmol, 1 equiv) were mixed in CD_3CN (0.5 mL) in a microwave react tube. The reaction mixture was stirred at 120 °C for 2 h in a microwave reactor. After cooling to room temperature, cage **3** was formed.

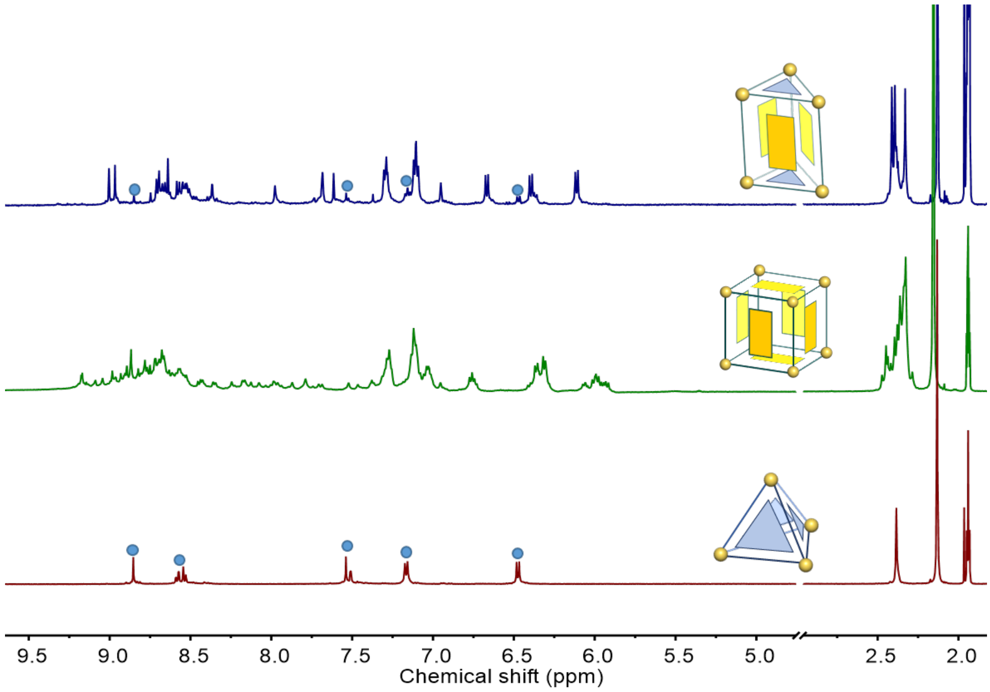

Figure S46. Comparison of ^{1}H NMR spectra (500 MHz, 298 K, CD_3CN) of cage **3** (top spectrum, blue) formed from mixing cage **1** and **4**, cage **1** (middle spectrum, green) and cage **4** (bottom spectrum, red). Trace amounts of **4** (marked with blue circles) remained after transformation.

4. Host-guest studies

4.1 General procedures

Host-guest complexes were prepared on an NMR scale and characterized by ^{1}H NMR spectroscopy. A 0.5 mM solution of cage **3** in CD_3CN was transferred to an NMR tube and 2 equivalents of guest molecules were added. The NMR tube was shaken and heated for 12 h at 50 °C after guest addition, and then the ^{1}H NMR spectrum, ^{19}F NMR spectrum were measured to demonstrate the guest encapsulation. For NMR titrations, known volumes of the guest solution were added into the host solution in an NMR tube. ^{1}H NMR spectra were collected after shaking and sonicating the host-guest mixture for 1-5 minutes.

4.2 **G1** binding within cage **3**

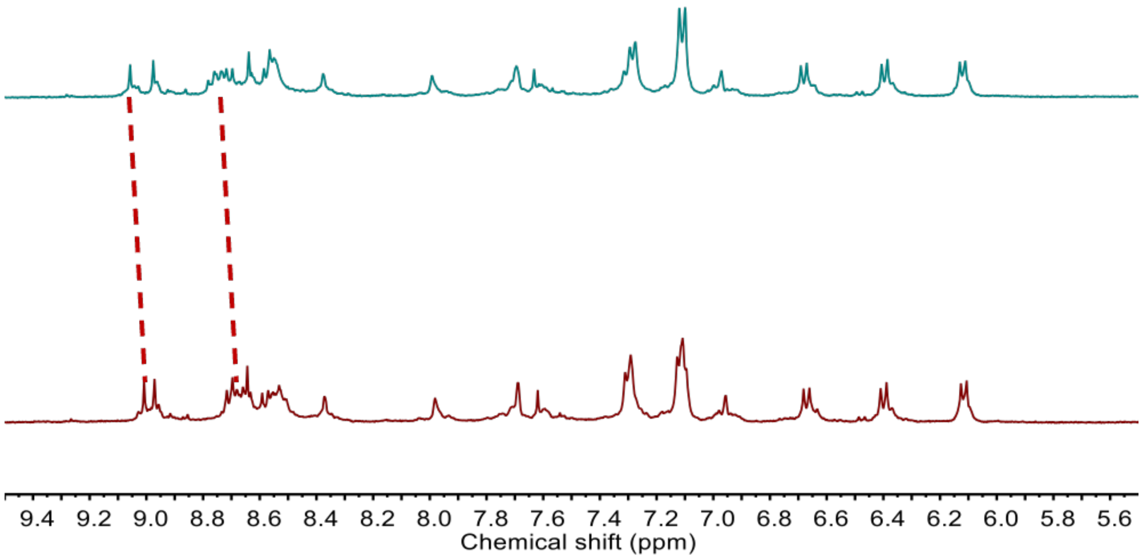

Figure S47. ^{1}H NMR (400 MHz, CD_3CN, 298 K) spectra of **G1⊂3** (top spectrum, green) and **3** (bottom spectrum, red).

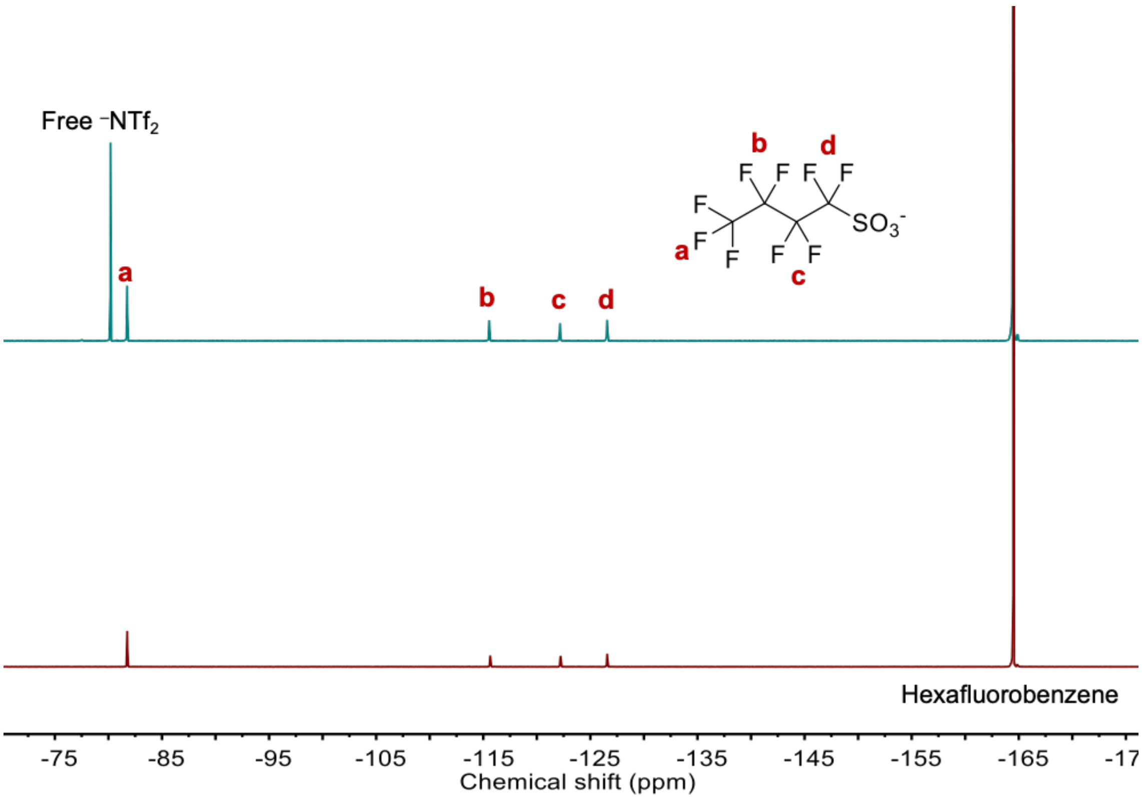

Figure S48. ^{19}F NMR (471 MHz, CD_3CN, 298 K) spectra of **G1⊂3** (up, green) and **G1** (below, red). 2 equivalents of **G1** was added to **3**. Hexafluorobenzene was added as the internal standard.

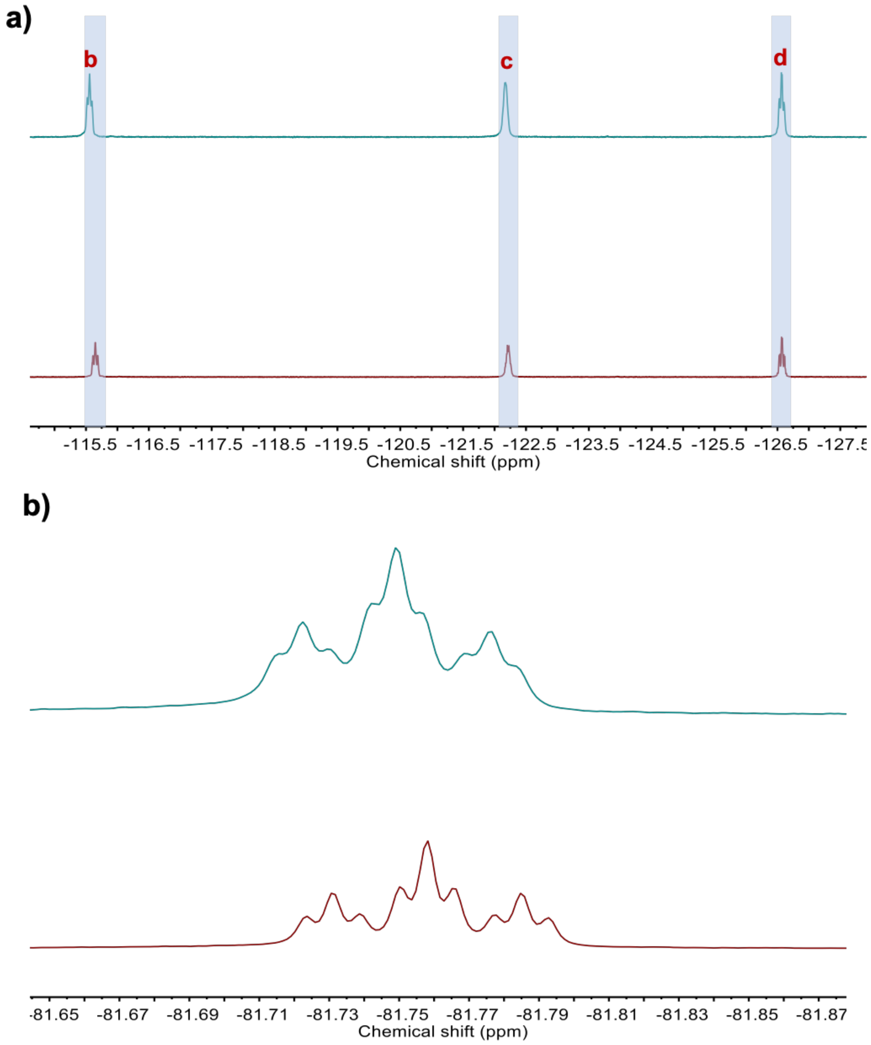

Figure S49. Partial ^{19}F NMR (471 MHz, CD_3CN, 298 K) spectra of **G1⊂3** (up, green) and **G1** (below, red) for **a)** fluorine b-d and **b)** fluorine b. Hexafluorobenzene was added as the internal standard.

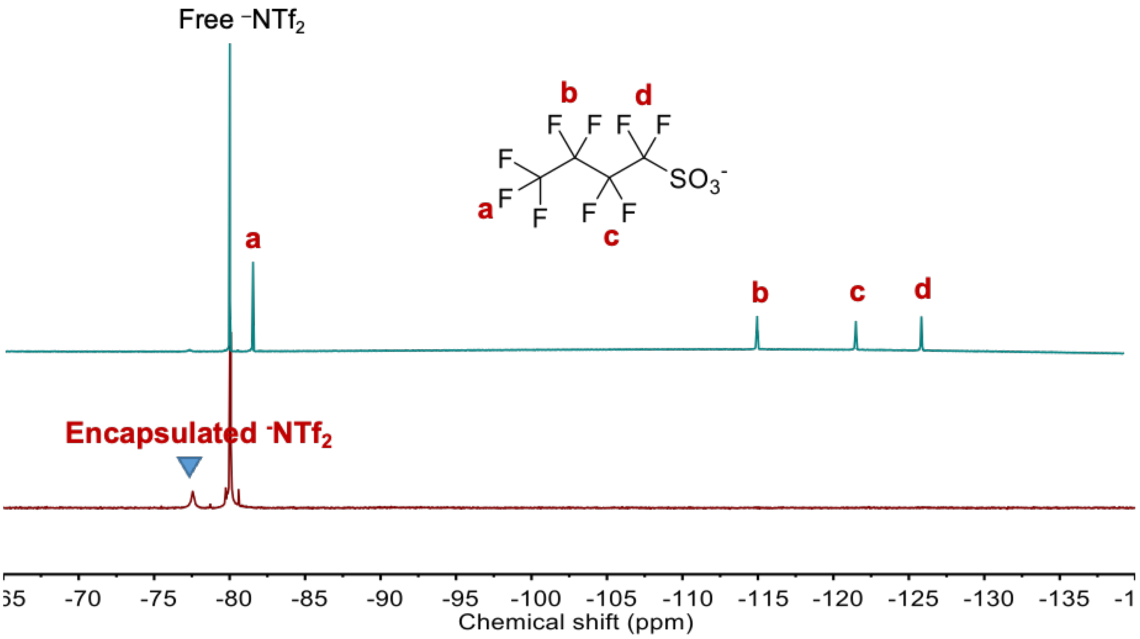

Figure S50. ^{19}F NMR (471 MHz, CD_3CN, 298 K) spectra of **G1⊂3** (top, green) and **3** (bottom, red). 2 equivalents of **G1** was added to **3**. The signal of encapsulated $^{-}NTf_2$ is significantly decreased after addition of **G1**.

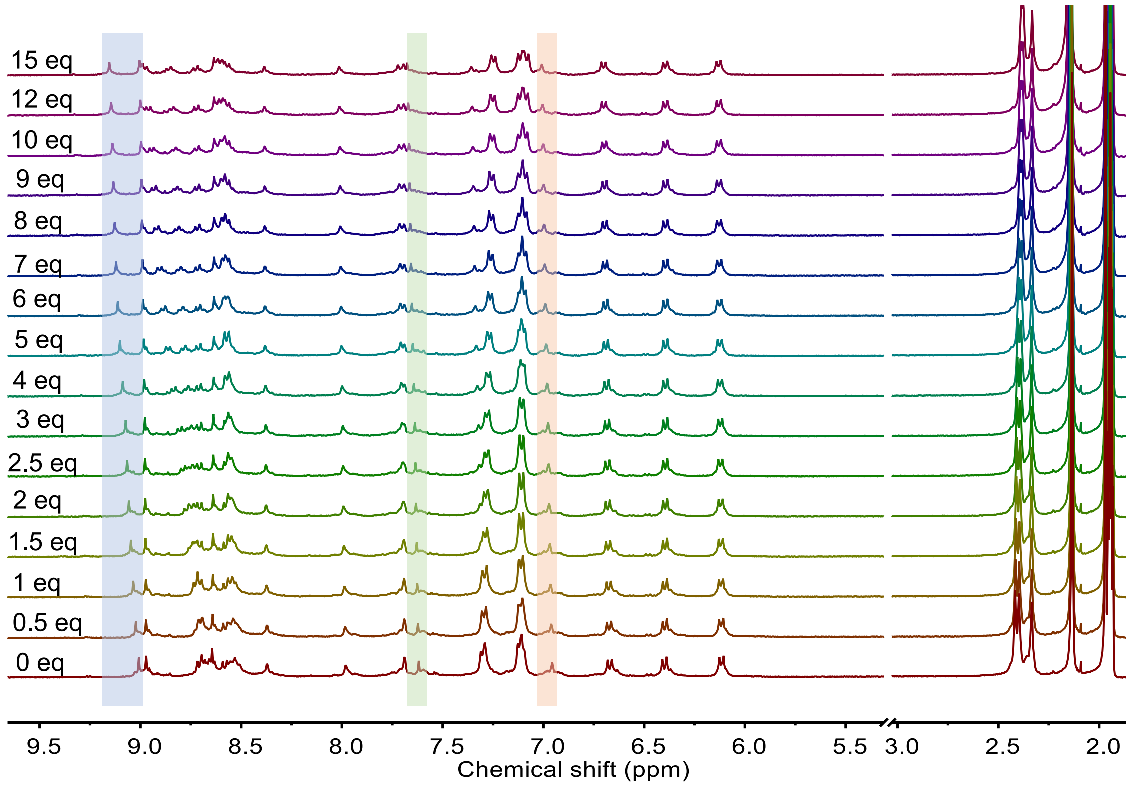

Figure S51. ^{1}H NMR Titration of cage **3** with **G1**.

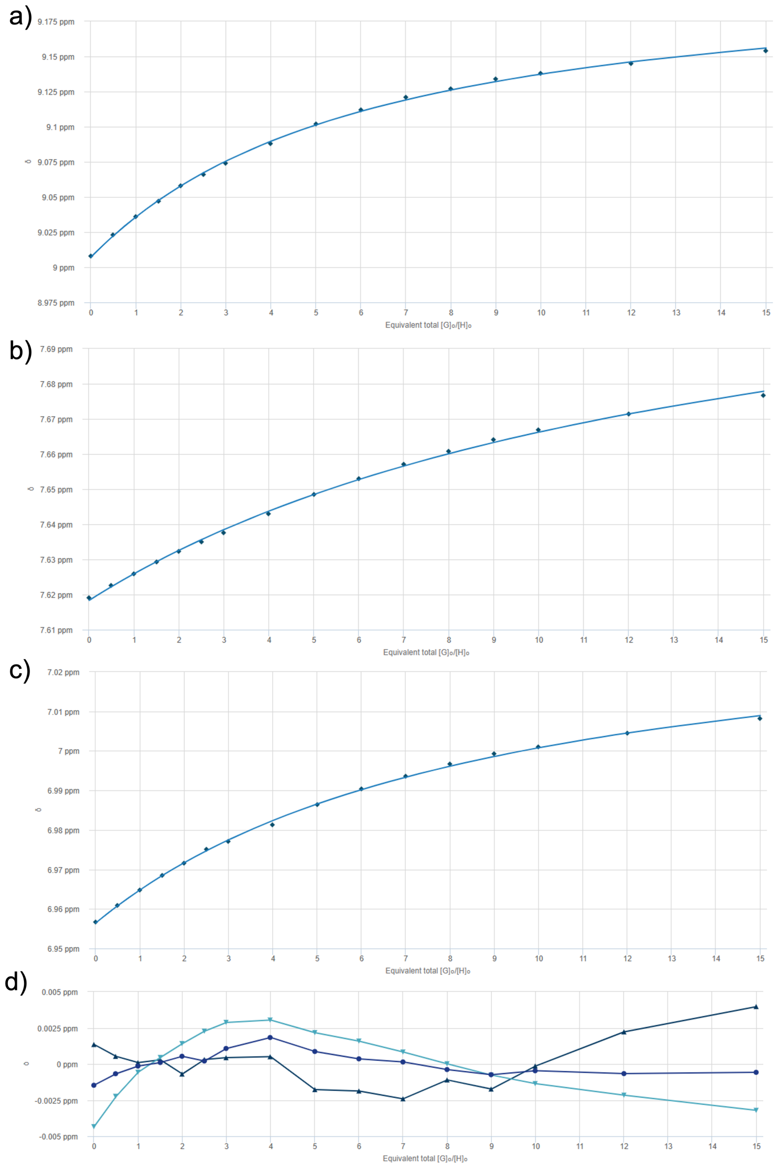

Details

Time to fit	0.1280 s
SSR	2.1159e-4
Fitted datapoints	48
Fitted params	4

Parameters

Parameter (bounds)	Optimised	Error	Initial
K (0 → ∞)	288.79 M⁻¹	± 2.1547 %	100.00 M⁻¹

Figure S52. Residual plots of ^{1}H NMR shift change of the **a)** peak in the blue box, **b)** peak in the green box and **c)** peak in the orange box in Figure S51. **d)** Binding isotherms for the binding of **G1** by **3** using BindFit[8] with a 1:1 binding mode, $K_a = 289 \pm 6$ M^{-1}.

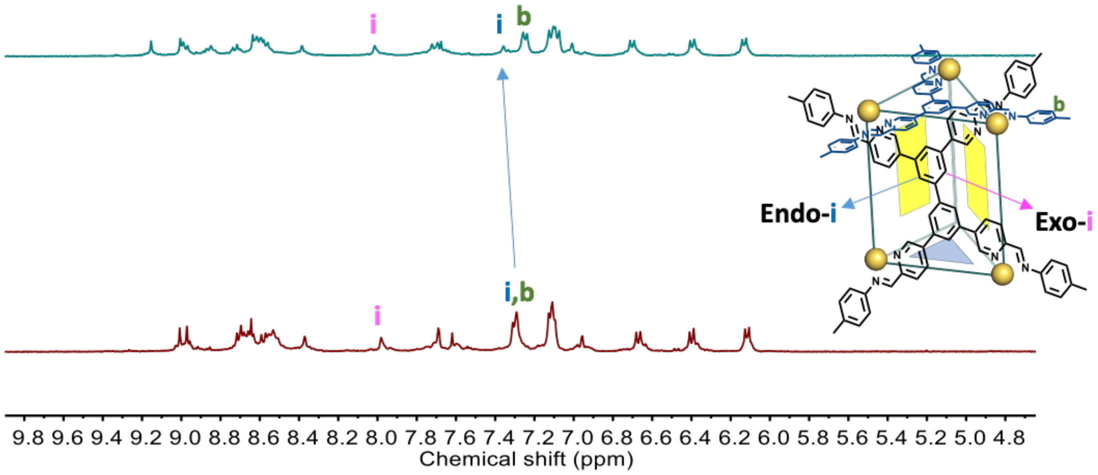

Figure S53. ^{1}H NMR (400 MHz, CD_3CN, 298 K) spectra of **G1⊂3** at the end (top spectrum, green) and beginning (bottom spectrum, red) of titration. The inward-pointing proton i was shifted, while the outward-pointing proton i exhibited minimal shift.

4.3 **G2** binding within cage **3**

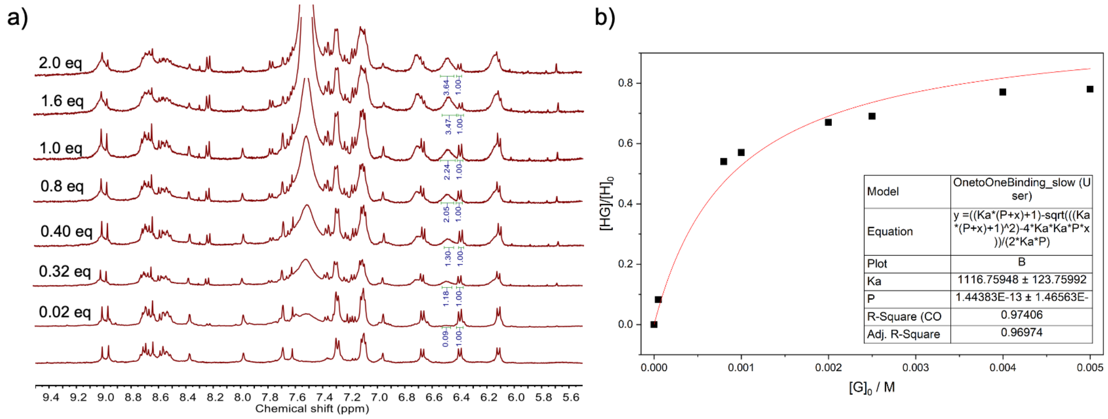

Figure S54. a) ^{1}H NMR stacked plot of the aromatic region during the titration of **G2** into a solution of cage **3** (400 MHz, 298 K, CD_3CN, [**3**] = 0.25 mM). b) Degree of encapsulation, $[HG]/[H]_0$, as a function of the total solution concentration of **G2** (black dots) and corresponding fit of the data to the 1:1 binding model (red curve), revealing a binding constant of 1117 ± 124 M^{-1} for the formation of complex [**G2**⊂**3**].

4.4 Non-binding prospective guests

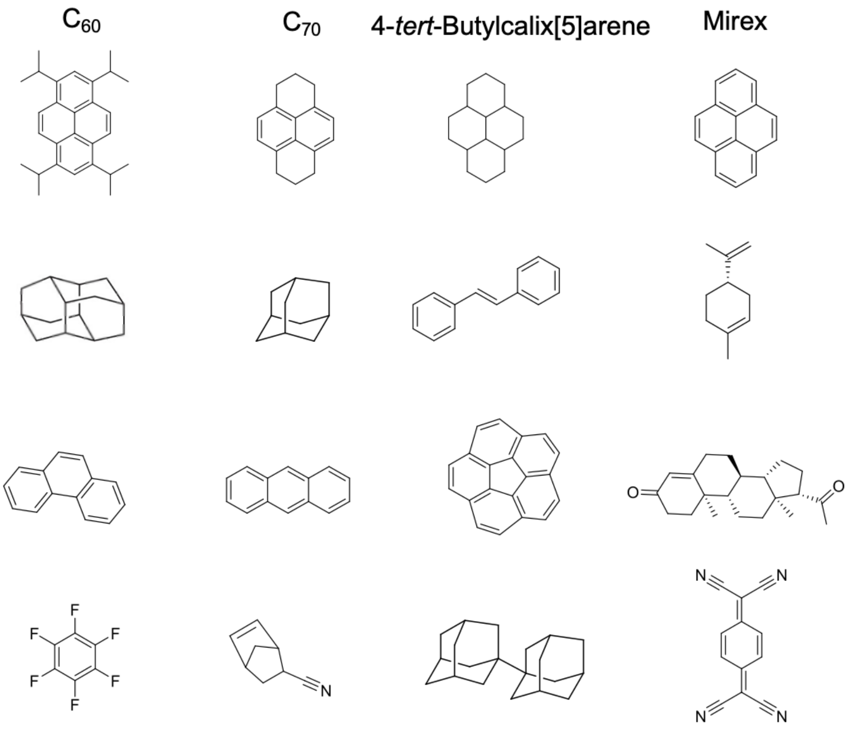

Figure S55. Non-binding prospective guests tested for cage **1**.

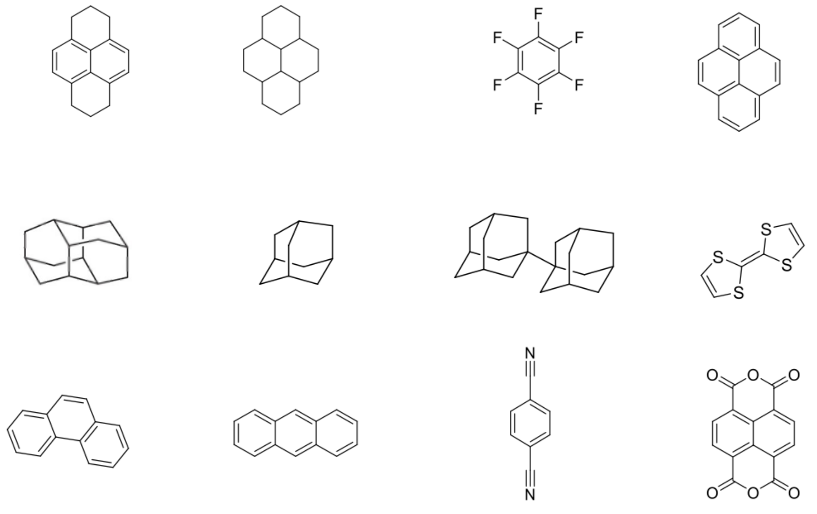

Figure S56. Non-binding prospective guests tested for cage **3**.

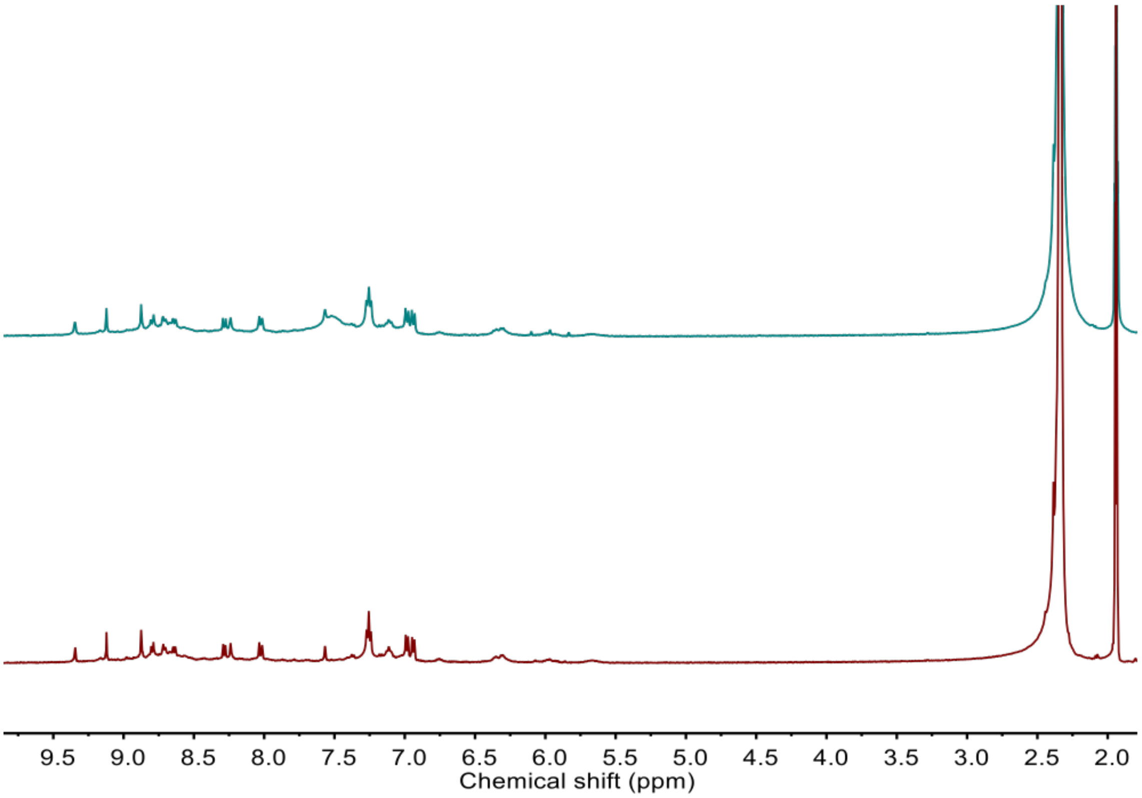

Figure S57. ^{1}H NMR (400 MHz, CD_3CN, 298 K) spectra of **2** following the addition of **G2** (top spectrum, green) and **2** alone (bottom spectrum, red). Negligible chemical shift changes were observed after the addition of **G2** to **2**.

5. X-ray Crystallography

Data were collected at Beamline I19 of Diamond Light Source employing silicon double crystal monochromated synchrotron radiation (0.6889 Å) with ω and ψ scans at 100(2)K.[9] Data integration and reduction were undertaken with Xia2.[10-12] Subsequent computations were carried out using the WinGX-32 graphical user interface.[13] Multiscan empirical absorption corrections were applied to the data using the AIMLESS[14] tool in the CCP4 suite.[15] The structures were solved by direct methods using SHELXT[16] then refined and extended with SHELXL.[17] In general, non-hydrogen atoms with occupancies greater than 0.5 were refined anisotropically. Carbon-bound hydrogen atoms were included in idealised positions and refined using a riding model. Disorder was modelled using standard crystallographic methods including constraints, restraints and rigid bodies where necessary. Crystallographic data along with specific details pertaining to the refinement follow. Crystallographic data have been deposited with the CCDC (2488117-2488120).

5.1 Crystal structure of cage **1**

[1]·16PF$_6$·5MeCN [+ solvent]

Formula $C_{394}H_{315}F_{96}N_{53}P_{16}Zn_8$, M 8634.45, Orthorhombic, space group F d d 2 (#43), a 56.5694(3), b 75.8830(3), c 23.4185(2) Å, V 100527.6(11) Å^3, D_c 1.141 g cm^{-3}, Z 8, crystal size 0.040 by 0.030 by 0.030 mm, colour pale yellow, habit block, temperature 100(2) Kelvin, λ(Synchrotron) 0.6889 Å, μ(Synchrotron) 0.468 mm^{-1}, T(Analytical)$_{min,max}$ 0.9177692132250895, 1.0, $2\theta_{max}$ 38.30, hkl range -53 53, -72 72, -22 22, N 76318, N_{ind} 22437(R_{merge} 0.0368), N_{obs} 12086(I > 2σ(I)), N_{var} 2152, residuals* $R1(F)$ 0.0711, $wR2(F^2)$ 0.1952, GoF(all) 0.852, $\Delta\rho_{min,max}$ -0.250, 0.389 e$^-$ Å^{-3}.

*$R1 = \Sigma||F_o| - |F_c||/\Sigma|F_o|$ for $F_o > 2\sigma(F_o)$; $wR2 = (\Sigma w(F_o^2 - F_c^2)^2/\Sigma(wF_c^2)^2)^{1/2}$ all reflections, $w=1/[\sigma^2(F_o^2)+(0.1369P)^2]$ where $P=(F_o^2+2F_c^2)/3$

Specific refinement details:

The crystals of [**1**]·16PF$_6$·5MeCN [+ solvent] were grown by diffusion of diethyl ether into an acetonitrile solution of [**1**]·16NTf$_2$ containing excess TBAPF$_6$. The crystals

employed immediately lost solvent after removal from the mother liquor and rapid handling prior to flash cooling in liquid nitrogen was required to collect data. Despite these measures and the use of synchrotron radiation few reflections at greater than 1.05 Å resolution were observed and the data were trimmed accordingly. The asymmetric unit was found to contain one half of a Zn_8L_6 assembly and associated counterions and solvent molecules. The structure was refined as a racemic twin with the Flack parameter refining to 0.12(2).

Due to the limited resolution bond lengths and angles within pairs of chemically identical organic ligands were restrained to be similar to each other. Additional DFIX and DANG restraints were applied to some areas of the structure displaying a high degree of thermal motion and some aromatic rings were modelled as rigid groups (AFIX 66). Thermal parameter restraints (SIMU, RIGU) were applied to all atoms except for zinc. However even with these restraints some thermal parameters remain higher than ideal, especially around the flexible toluidine groups.

The anions within the structure show evidence of substantial disorder. Only five lattice sites could be located for the hexafluorophosphate anions, all of which were modelled with partial occupancy. Additional lower occupancy disordered locations could not be resolved for these anions. Substantial bond length and thermal parameter restraints were applied to facilitate stable refinement of the anions and most low occupancy anions were modelled with isotropic thermal parameters. The occupancies of all located anions were allowed to freely refine and then fixed at the refined values which resulted in a discrepancy of 10.8 anions per **1** assembly (or 5.4 per asymmetric unit). The hydrogen atoms of several low occupancy acetonitrile molecule could not be located in the electron density map and were therefore not included in the model.

The remaining anions (included as hexafluorophosphate in the formula) and solvent within the lattice were significantly disordered and despite numerous attempts at modelling, including with rigid bodies no satisfactory model for the electron-density associated with them could be found. Consequently the SQUEEZE[18] function of PLATON[19] was employed to remove the contribution of the electron density associated with these remaining anions and further highly disordered solvent, which gave a potential

solvent accessible void of 36370 Å^3 per unit cell (a total of approximately 8531 electrons). The diffuse solvent molecules could not be assigned to acetonitrile or diethyl ether and were not included in the formula. Consequently, the molecular weight and density given above are underestimated.
CheckCIF gives one A and three B level alert. These alerts mostly result from the limited resolution of the data (low sine(theta_max)/wavelength, low bond precision, poor data / parameter ratio). One B level alert for missing reflections below Theta(Min) may arise from reflections effected by the beamstop or overloaded low angle data.

5.2 Crystal structure of cage **1'**

[1']·14NTf$_2$·2ClO$_4$·14MeCN [+ solvent]

Formula $C_{440}H_{342}Cl_2F_{84}N_{76}O_{88}S_{28}Zn_8$, *M* 11189.42, Trigonal, space group R -3 c (#167), *a* 59.08320(10), *b* 59.08320(10), *c* 85.7309(2) Å, γ 120°, *V* 259176.6(11) Å^3, D_c 1.290 g cm^{-3}, *Z* 18, crystal size 0.030 by 0.025 by 0.020 mm, colour pale yellow, habit block, temperature 100(2) Kelvin, λ(Synchrotron) 0.6889 Å, μ(Synchrotron) 0.490 mm^{-1}, T(Analytical)$_{min,max}$ 0.9114154911478537, 1.0, $2\theta_{max}$ 45.00, *hkl* range -65 65, -60 63, -94 90, *N* 360604, N_{ind} 40913(R_{merge} 0.0560), N_{obs} 24382(I > 2σ(I)), N_{var} 3333, residuals* $R1(F)$ 0.1285, $wR2(F^2)$ 0.4160, GoF(all) 1.033, $\Delta\rho_{min,max}$ -0.758, 1.175 e$^-$ Å^{-3}.

*$R1 = \Sigma||F_o| - |F_c||/\Sigma|F_o|$ for $F_o > 2\sigma(F_o)$; $wR2 = (\Sigma w(F_o^2 - F_c^2)^2/\Sigma(wF_c^2)^2)^{1/2}$ all reflections, $w=1/[\sigma^2(F_o^2)+(0.3313P)^2]$ where $P=(F_o^2+2F_c^2)/3$

Specific refinement details:

The crystals of [**1'**]·14NTf$_2$·2ClO$_4$·14MeCN [+ solvent] were grown by diffusion of diethyl ether into an acetonitrile solution of [**1'**]·16NTf$_2$ containing a small amount of TBAClO$_4$. The crystals employed immediately lost solvent after removal from the mother liquor and rapid handling prior to flash cooling in liquid nitrogen was required to collect data. Despite these measures and the use of synchrotron radiation few reflections at

greater than 0.9 Å resolution were observed and the data were trimmed accordingly. The asymmetric unit was found to contain one half of a Zn_8L_6 assembly and associated counterions and solvent molecules.

Due to the limited resolution bond lengths and angles within pairs of chemically identical organic ligands were restrained to be similar to each other. Additional DFIX and DANG restraints were applied to some areas of the structure displaying a high degree of thermal motion and some aromatic rings were modelled as rigid groups (AFIX 66). Thermal parameter restraints (SIMU, RIGU) were applied to all atoms except for zinc. Five of the outer anisidine groups were modelled as disordered over two locations. These groups were all modelled with isotropic thermal parameters. Some thermal parameters remain higher than ideal, especially around the remaining flexible anisidine groups indicating further unresolved disorder or thermal motion. There is one B level alert resulting from a close contact between hydrogen atoms of anisidine residues on neighboring molecules which might be further disordered. Furthermore the hydrogen atoms of the methyl group of the anisidine residues are poorly resolved and could only be modelled with calculated positions so may not be accurately located.

The anions within the structure show evidence of substantial disorder. Most of the located triflimide anions and the perchlorate anion were modelled as disordered over two or three locations and/or with partial occupancy. Additional lower occupancy disordered locations could not be resolved for these anions. Substantial bond length and thermal parameter restraints were applied to facilitate stable refinement of the anions and most low occupancy anions were modelled with isotropic thermal parameters. The occupancies of all located anions were allowed to freely refine which resulted in a discrepancy of 3.7 anions per Zn_8L_6 assembly (or 1.9 per asymmetric unit, included as triflimide in the formula). The hydrogen atoms of several low occupancy or disordered acetonitrile molecules could not be located in the electron density map and were therefore not included in the model. The very high levels of disorder within both the cationic and anionic parts of the structure and difficulties resolving this disorder due to the limited resolution of the data result in values of the R1, wR and wR2 factors that are larger than for typical small molecule structures.

The remaining anions and solvent within the lattice were significantly disordered and despite numerous attempts at modelling, including with rigid bodies no satisfactory model for the electron-density associated with them could be found. Consequently the SQUEEZE[18] function of PLATON[19] was employed to remove the contribution of the electron density associated with these remaining anions and further highly disordered solvent, which gave a potential solvent accessible void of 49033 Å^3 per unit cell (a total of approximately 5625 electrons). The diffuse solvent molecules could not be assigned to acetonitrile or diethyl ether and were also not included in the formula. Consequently, the molecular weight and density given above are underestimated.

CheckCIF gives one A and three B level alerts. The A level alert results from isotropic modelling of the disordered anisidine groups. The B level alerts result from the limited resolution of the data (low sine(theta_max)/wavelength), high amounts of disorder and thermal motion (high wR2) and the short contact between anisidine residues on neighbouring molecules as describe above.

5.3 Crystal structure of cage **3**

[3]·12NTf$_2$·2.25MeCN [+ solvent]

Formula $C_{310.50}H_{228.75}F_{72}N_{50.25}O_{48}S_{24}Zn_6$, *M* 7961.33, Trigonal, space group P -3 c 1 (#165), *a* 31.42410(10), *b* 31.42410(10), *c* 45.9361(2) Å, γ 120°, *V* 39283.5(3) Å^3, D_c 1.346 g cm^{-3}, *Z* 4, crystal size 0.080 by 0.060 by 0.020 mm, colour colourless, habit block, temperature 100(2) Kelvin, λ(Synchrotron) 0.6889 Å, μ(Synchrotron) 0.539 mm^{-1}, T(Analytical)$_{min,max}$ 0.9428136610884578, 1.0, $2\theta_{max}$ 33.36, *hkl* range -26 26, -26 26, -38 38, *N* 78385, N_{ind} 7951(R_{merge} 0.0476), N_{obs} 3702(I > 2σ(I)), N_{var} 1447, residuals* $R1(F)$ 0.1462, $wR2(F^2)$ 0.4195, GoF(all) 0.950, $\Delta\rho_{min,max}$ -0.465, 0.681 e$^-$ Å^{-3}.

$^*R1 = \Sigma||F_o| - |F_c||/\Sigma|F_o|$ for $F_o > 2\sigma(F_o)$; $wR2 = (\Sigma w(F_o^2 - F_c^2)^2/\Sigma(wF_c^2)^2)^{1/2}$ all reflections

$w=1/[\sigma^2(F_o^2)+(0.3440P)^2]$ where $P=(F_o^2+2F_c^2)/3$

Specific refinement details:

The crystals of [**3**]·12NTf$_2$·2.25MeCN [+ solvent] were grown by diffusion of diisopropyl ether into an acetonitrile solution of the complex. The crystals employed immediately lost solvent after removal from the mother liquor and rapid handling prior to flash cooling in liquid nitrogen was required to collect data. Despite these measures and the use of synchrotron radiation few reflections at greater than 1.2 Å resolution were observed and the data were trimmed accordingly. The diffraction was broad and the quality of the integration is less than ideal hence the values of the R1, wR and wR2 factors are larger than for typical small molecule structures. Nevertheless, the quality of the data is far more than sufficient to establish the connectivity of the structure. The asymmetric unit was found to contain one third of a $Zn_6L^A{}_3L^C{}_2$ assembly and associated counterions and solvent molecules.

Due to the limited resolution of the data all phenyl rings within the structure were refined as rigid groups (AFIX 66). Some additional DFIX and DANG restraints were applied to areas of the structure that displayed a high degree of thermal motion. Thermal parameter restraints (SIMU, RIGU) were also applied to all atoms except for zinc to facilitate anisotropic refinement. Even with these restraints the thermal parameters of both the main residue and the anions remain higher than ideal due to the large amount of thermal motion present throughout the structure.

The located triflimide counterions, one of which was disordered over a special position, were modelled with partial occupancy. Their occupancies were initially refined and then fixed close to the obtained values. Substantial bond length and thermal parameter restraints were applied to facilitate stable refinement of the anions. There is evidence of additional disorder of the counterions (high thermal parameters, residual electron density close to the modelled counterions) which could not be resolved due to the limited resolution of the data. The remaining anions (6.45 per **3** assembly or 2.15 per asymmetric unit, included as triflimide in the formula) and solvent within the lattice were significantly disordered and despite numerous attempts at modelling, including with rigid bodies no satisfactory model for the electron-density associated with them could be found.

Consequently the SQUEEZE[18] function of PLATON[19] was employed to remove the contribution of the electron density associated with these remaining anions and further highly disordered solvent, which gave a potential solvent accessible void of 9035 Å^3 per unit cell (a total of approximately 1836 electrons). The diffuse solvent molecules could not be assigned to acetonitrile or diisopropyl ether and were also not included in the formula. Consequently, the molecular weight and density given above are underestimated.

CheckCIF gives two A and four B level alerts. These alerts all result from the limited resolution of the data (low sine(theta_max)/wavelength, low bond precision, poor data/parameter ratio), poor diffraction properties of the crystals (high wR2 value, low bond precision) and unresolved disorder of the anions (large average Ueq values) as described above.

5.4 Crystal structure of **G2⊂3**

[G2 ⊂ 3]·12NTf$_2$·5.25MeCN [+ solvent]

Formula $C_{328.50}H_{241.75}F_{72}N_{57.25}O_{48}S_{24}Zn_6$, *M* 8288.69, Trigonal, space group P -3 c 1 (#165), *a* 31.3970(6), *b* 31.3970(6), *c* 45.8322(8) Å, γ 120°, *V* 39127.1(16) Å^3, D_c 1.407 g cm^{-3}, *Z* 4, crystal size 0.100 by 0.080 by 0.050 mm, colour dark green, habit block, temperature 100(2) Kelvin, λ(Synchrotron) 0.6889 Å, μ(Synchrotron) 0.544 mm^{-1}, T(Analytical)$_{min,max}$ 0.9281998534142097, 1.0, $2\theta_{max}$ 30.73, *hkl* range -24 24, -24 23, -35 35, *N* 57089, N_{ind} 6205(R_{merge} 0.0652), N_{obs} 2806(I > 2σ(I)), N_{var} 1273, residuals* $R1(F)$ 0.1580, $wR2(F^2)$ 0.4347, GoF(all) 1.513, $\Delta\rho_{min,max}$ -0.547, 0.886 e$^-$ Å^{-3}.

$^*R1 = \Sigma||F_o| - |F_c||/\Sigma|F_o|$ for $F_o > 2\sigma(F_o)$; $wR2 = (\Sigma w(F_o^2 - F_c^2)^2/\Sigma(wF_c^2)^2)^{1/2}$ all reflections, $w=1/[\sigma^2(F_o^2)+(0.2000P)^2]$ where $P=(F_o^2+2F_c^2)/3$

Specific refinement details:

The crystals [**G2** ⊂ **3**]·12NTf$_2$·5.25MeCN [+ solvent] were grown by diffusion of diethyl

ether into an acetonitrile solution the complex. The crystals employed immediately lost solvent after removal from the mother liquor and rapid handling prior to flash cooling in liquid nitrogen was required to collect data. Despite these measures and the use of synchrotron radiation few reflections at greater than 1.3 Å resolution were observed and the data were trimmed accordingly. The diffraction was broad and the quality of the integration is less than ideal hence the values of the R1, wR and wR2 factors are larger than for typical small molecule structures. Nevertheless, the quality of the data is far more than sufficient to establish the connectivity of the structure and encapsulation of TCNQ. The asymmetric unit was found to contain one third of a $Zn_6L^A{}_3L^C{}_2$ assembly, one third of the disordered guest **G2** and associated counterions and solvent molecules.

Due to the limited resolution of the data and high degree of thermal motion throughout the structure, bond length and angle restraints were required in order to obtain a reasonable model for the organic parts of the structure. The GRADE program[20] was thus employed, using the GRADE Web Server,[21] to generate a full set of bond distance and angle restraints (DFIX, DANG, FLAT) for both types of organic ligands. Thermal parameter restraints (SIMU, RIGU) were also applied to all atoms except for zinc to facilitate anisotropic refinement. Even with these restraints the thermal parameters of both the main residue and the anions remain higher than ideal.

The guest **G2** was modelled as disordered by symmetry around the 3-fold axis through the center of the trigonal prism with overall full occupancy. Extensive DFIX, DANG and FLAT restraints (also calculated using the GRADE Web Server[21]) were employed to enable the disordered guest to be modelled.

Only two triflimide counterion lattice sites could be located in the electron density map, one of which was modelled as disordered over two sites. Their occupancies were initially refined and then fixed close to the obtained values. Substantial bond length and thermal parameter restraints were applied to facilitate stable refinement of the anions. There is evidence of additional disorder of the counterions (high thermal parameters, residual electron density close to the modelled counterions) which could not be resolved due to the limited resolution of the data. The remaining anions (8.4 per cage **3** assembly or 2.8 per asymmetric unit, included as triflimide in the formula) and solvent within the lattice

were significantly disordered and despite numerous attempts at modelling, including with rigid bodies no satisfactory model for the electron-density associated with them could be found. Consequently the SQUEEZE[18] function of PLATON[19] was employed to remove the contribution of the electron density associated with these remaining anions and further highly disordered solvent, which gave a potential solvent accessible void of 11013 Å^3 per unit cell (a total of approximately 3519 electrons). The diffuse solvent molecules could not be assigned to acetonitrile or diethyl ether and were also not included in the formula. Consequently, the molecular weight and density given above are underestimated. The hydrogen atoms of the acetonitrile molecules could not be located in the electron density map and were therefore not included in the model.

CheckCIF gives two A and four B level alerts. These alerts all result from the limited resolution of the data (low sine(theta_max)/wavelength, low bond precision, poor data/parameter ratio), poor diffraction properties of the crystals (high R1 and wR2 values, low bond precision) and acetonitrile molecules for which hydrogen atoms were not modelled (apparent singly bonded carbon atoms).

5.5 Measurement of parameters in cage **1** and **1'**

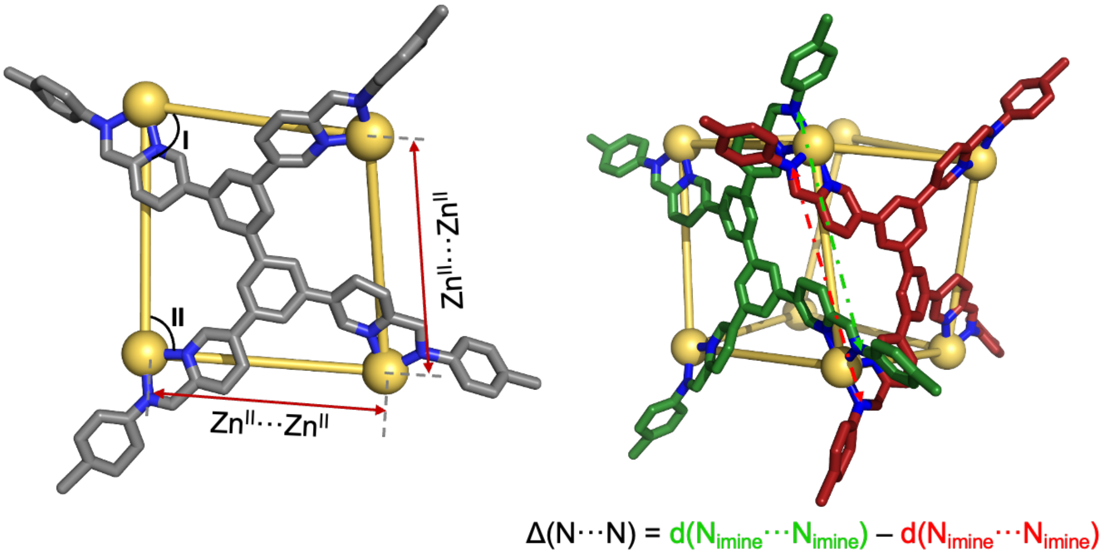

Figure S58. Illustration of $Zn^{II}\cdots Zn^{II}$ distances and $\Delta(N_{imine}\cdots N_{imine})$ distances in cages **1** and **1'**.

**Cage**	**Δ(N···N) (Å)**	**$Zn^{II}\cdots Zn^{II}$ Distance (Å)**
**1**	0.13±0.07	11.5±0.3
**1'**	0.1±0.02	11.4±0.3

Table S1. The average $Zn^{II}\cdots Zn^{II}$ distances and $\Delta(N_{imine}\cdots N_{imine})$ distances for different diastereomers of *pseudo*-cube **1**.

6. Volume Calculations

The volumes of the interior cavities of metal-organic cages **1, 1'** and **3** were calculated from the crystal structure of each architecture using MoloVol.[22] The "probe-occupied volume" (V_{occ}) was calculated in each case in "single-probe mode", using the following parameters:

Small probe radius: 1.2 Å

Grid resolution: 0.2 Å

Optimisation depth: 4 (default value)

Element radii. Zn: 2.39 Å, N: 1.66 Å, H: 1.20 Å, C: 1.77 Å

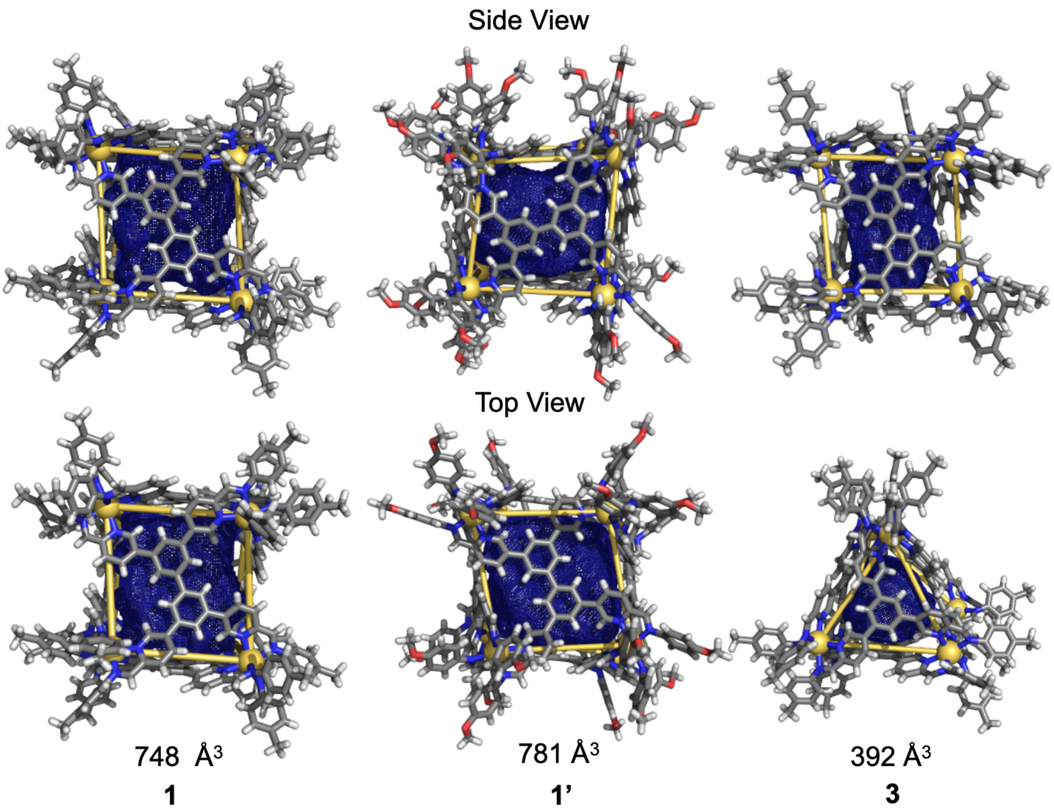

Figure S59. MoloVol[22] calculated void space (navy blue mesh) within the crystal structures of **1, 1'** and **3**.

7. Electrochemistry

Electrochemical experiments were performed with a SP-150 potentiostat manufactured by BioLogic. Measurements were conducted under N_2 atmosphere with a glassy carbon working electrode (diameter 3 mm) and a platinum auxiliary electrode at a scan rate of 60 mV s^{-1}. A silver wire was used as reference electrode. All three electrodes were immersed in a CH_3CN solution (5 mL) containing $TBANTf_2$ (approx. 0.1 M) as a supporting electrolyte and **G2** (1 mM) or cage **3** (approx. 0.5 mM). For **G2** ⊂ **3**, 1 equivalent of **G2** was added to the cage **3** solution. In all cases, ferrocene was used as an internal standard, and all reduction potentials are reported using Fc/Fc+ redox couple as reference. To minimize possible competition from the $^{-}NTf_2$ anions of the supporting electrolyte, CV measurements were performed immediately after the addition of $TBANTf_2$. No changes in the voltammograms were observed over successive scans, suggesting that the guest within cage **3** remained encapsulated under the measurement conditions.

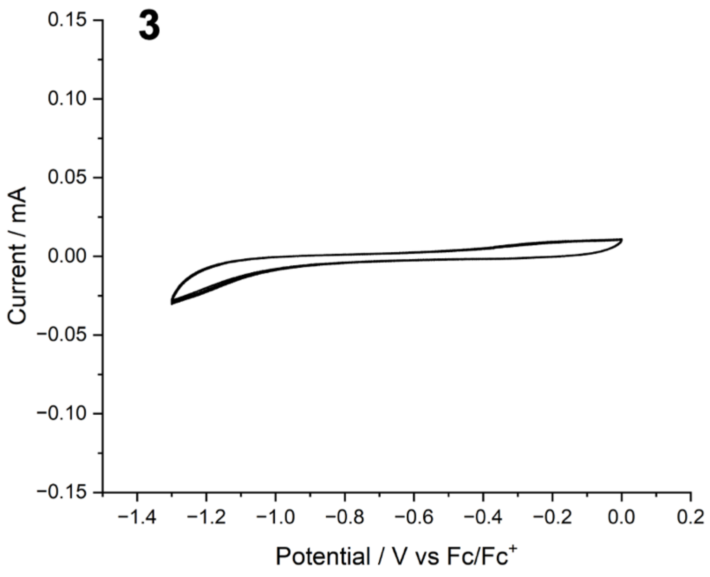

Figure S60. Cyclic voltammetry (three scans) of cage **3** in CH_3CN (0.1 M nBu$_4$N$^+$Tf$_2$N$^-$) at room temperature.

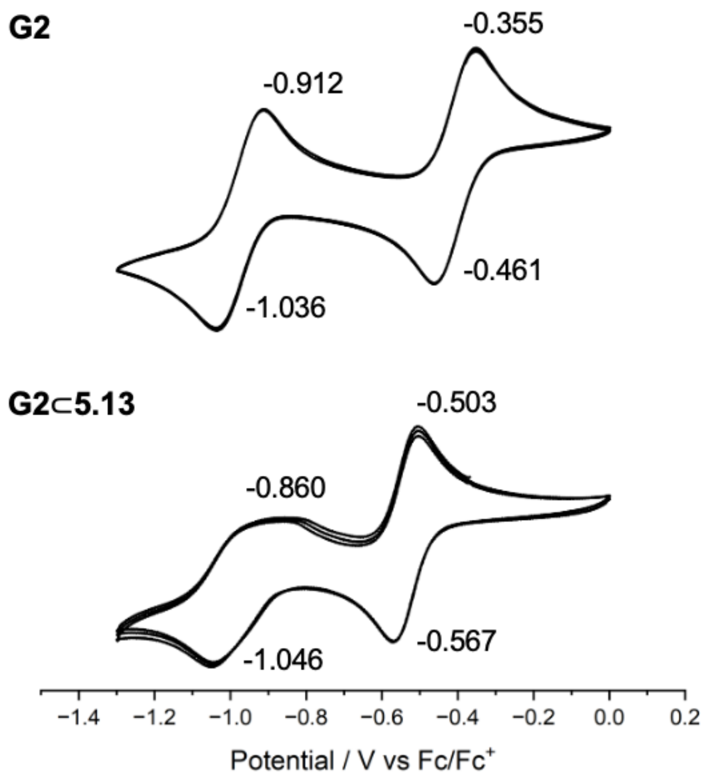

Figure S61. Cyclic voltammetry (three scans) of **G2** (up) and **G2** ⊂ **3** (low) in CH_3CN (0.1 M $n$$Bu_4N^+Tf_2N^-$) at room temperature.

8. References

[1] Castilla, A. M.; Ronson, T. K.; Nitschke, J. R. Sequence-Dependent Guest Release Triggered by Orthogonal Chemical Signals. *J. Am. Chem. Soc.* **2016**, *138*, 2342–2351.

[2] Plajer, A. J.; Percástegui, E. G.; Santella, M.; Rizzuto, F. J.; Gan, Q.; Laursen, B. W.; Nitschke, J. R. Fluorometric Recognition of Nucleotides within A Water-Soluble Tetrahedral Capsule. *Angew. Chem. Int. Ed.* **2019**, *58*, 4200–4204.

[3] Davies, J. A.; Tarzia, A.; Ronson, T. K.; Auras, F.; Jelfs, K. E.; Nitschke, J. R. Tetramine Aspect Ratio and Flexibility Determine Framework Symmetry for Zn_8L_6 Self-Assembled Structures. *Angew. Chem. Int. Ed.* **2023**, *135*, e202217987.

[4] Jiao, J.; Li, Z.; Qiao, Z.; Li, X.; Liu, Y.; Dong, J.; Jiang, J.; Cui, Y. Design and Self-Assembly of Hexahedral Coordination Cages for Cascade Reactions. *Nat. Comm.* **2018**, *9*, 1–8.

[5] Liu, Y.; Lin, Z.; He, C.; Zhao, L.; Duan, C. A Symmetry-Controlled and Face-Driven Approach for the Assembly of Cerium-Based Molecular Polyhedra. *Dalton Trans.* **2010**, *39*, 11122–11125.

[6] Yang, L.; Jing, X.; He, C.; Chang, Z.; Duan, C. Redox-Active M_8L_6 Cubic Hosts with Tetraphenylethylene Faces Encapsulate Organic Dyes for Light-Driven H_2 Production. *Chem. Eur. J.* **2016**, *22*, 18107–18114.

[7] Ramsay, W. J.; Szczypiński, F. T.; Weissman, H.; Ronson, T. K.; Smulders, M. M. J.; Rybtchinski, B.; Nitschke, J. R. Designed Enclosure Enables Guest Binding Within the 4200 Å3 Cavity of a Self-Assembled Cube. Angew. Chem. Int. Ed. 2015, 54, 5636–5640.

[8] Brynn Hibbert, D.; Thordarson, P. The Death of The Job Plot, Transparency, Open Science and Online Tools, Uncertainty Estimation Methods and Other Developments in Supramolecular Chemistry Data Analysis. *Chem. Commun.*, **2016**, *52*, 12792–12805.

[9] Allan, D. .; Nowell, H.; Barnett, S.; Warren, M.; Wilcox, A.; Christensen, J.; Saunders, L.; Peach, A.; Hooper, M.; Zaja, L.; Patel, S.; Cahill, L.; Marshall, R.; Trimnell, S.; Foster, A.; Bates, T.; Lay, S.; Williams, M.; Hathaway, P.; Winter, G.; Gerstel, M.; Wooley, R. A Novel Dual Air-Bearing Fixed-χ Diffractometer for Small-Molecule Single-Crystal X-Ray Diffraction on Beamline I19 at Diamond Light Source. *Crystals* **2017**, *7*, 336.

[10] Gildea, R. J.; Winter, G. Determination of Patterson Group Symmetry from Sparse Multi-Crystal Data Sets in the Presence of an Indexing Ambiguity. *Acta Crystallogr. Sect.*

D Struct. Biol. **2018**, *74*, 405–410.

[11] Evans, P. Scaling and Assessment of Data Quality. *Acta Crystallogr. Sect. D Biol. Crystallogr.* **2006**, *62*, 72–82.

[12] Winter, G. Xia2: An Expert System for Macromolecular Crystallography Data Reduction. *J. Appl. Crystallogr.* **2010**, *43*, 186–190.

[13] Farrugia, L. J. WinGX and ORTEP for Windows: An Update. *J. Appl. Crystallogr.* **2012**, *45*, 849–854.

[14] Evans, P. R.; Murshudov, G. N. How Good Are My Data and What Is the Resolution? *Acta Crystallogr. Sect. D Biol. Crystallogr.* **2013**, *69*, 1204–1214.

[15] Winn, M. D.; Ballard, C. C.; Cowtan, K. D.; Dodson, E. J.; Emsley, P.; Evans, P. R.; Keegan, R. M.; Krissinel, E. B.; Leslie, A. G. W.; McCoy, A.; McNicholas, S. J.; Murshudov, G. N.; Pannu, N. S.; Potterton, E. A.; Powell, H. R.; Read, R. J.; Vagin, A.; Wilson, K. S. Overview of the CCP 4 Suite and Current Developments. *Acta Crystallogr. Sect. D Biol. Crystallogr.* **2011**, *67*, 235–242.

[16] Sheldrick, G. M. SHELXT –Integrated Space-Group and Crystal-Structure Determination. *Acta Crystallogr. Sect. A Found. Adv.* **2015**, *A71*, 3–8.

[17] Sheldrick, G. M. Crystal Structure Refinement with SHELXL. *Acta Crystallogr. Sect. C Struct. Chem.* **2015**, *C71*, 3–8.

[18] van der Sluis, P.; Spek, A. L. BYPASS: An Effective Method for The Refinement of Crystal Structures Containing Disordered Solvent Regions. *Acta Cryst.* **1990**, *A46*, 194-201.

[19] Spek, A. L. *PLATON: A Multipurpose Crystallographic Tool*; Utrecht University, 2008.

[20] Bricogne, G.; Blanc, E.; Brandle, M.; Flensburg, C.; Keller, P.; Paciorek, W.; Roversi, P.; Sharff, A.; Smart, O. S.; Vonrhein, C.; et al. *BUSTER*; Global Phasing Ltd., 2011.

[21] Smart, O. S.; Womack, T. O. *Grade Web Server*; Global Phasing Ltd., 2014.

[22] Maglic, J. B.; Lavendomme, R. MoloVol: An Easy-to-Use Program for Analyzing Cavities, Volumes and Surface Areas of Chemical Structures. *J. Appl. Crystallogr.* **2022**, *55*, 1033–1044.
